# Dioscin alleviates the progression of osteoarthritis: an in vitro and in vivo study

**DOI:** 10.1186/s12950-023-00339-w

**Published:** 2023-04-13

**Authors:** Qing Ding, Ruizhuo Zhang, Gaohong Sheng, Tianqi Wang, Shaoze Jing, Tian Ma, Shanxi Wang, Hongqi Zhao, Hua Wu, Wenkai Li

**Affiliations:** 1 Department of Pediatric Surgery, Tongji Hospital, Tongji Medical College, Huazhong University of Science and Technology, Wuhan, China; 2 Department of Orthopedics, Tongji Hospital, Tongji Medical College, Huazhong University of Science and Technology, Wuhan, China; 3 Department of Orthopedics, Shanxi Bethune Hospital, Shanxi Medical University, Taiyuan, China

**Keywords:** Dioscin, Osteoarthritis, Chondrocyte, Inflammatory cytokines, Cartilage matrix

## Abstract

Osteoarthritis (OA) is a common joint disease and is the main cause of physical disability in the elderly. Currently, there is no adequate therapeutic strategy to reverse the progression of OA. Many natural plant extracts have received attention in the treatment of OA due to their potential anti-inflammatory properties, and reduced incidence of adverse events. Dioscin (Dio), a natural steroid saponin, has been demonstrated to inhibit the release of inflammatory cytokines in mouse and rat models of various diseases, and has a protective effect in chronic inflammatory diseases. However, whether Dio alleviates OA progression remains to be explored. In this research, our purposes were to investigate the therapeutic potential of Dio in OA. The results demonstrated that Dio exerted anti-inflammatory effects by repressing NO, PGE_2_, iNOS and COX-2. Moreover, the application of Dio could repress IL-1β-induced overexpression of matrix metalloproteinases (MMPs, including MMP1, MMP3, and MMP13) and ADAMTS-5, and improve the synthesis of collagen II and aggrecan, which contribute to the maintenance of chondrocyte matrix homeostasis. The underlying mechanism involved the inhibition of the MAPK and NF-κB signaling pathways by Dio. Furthermore, the treatment of Dio significantly improved the pain behaviors of rat OA models. The in vivo study revealed that Dio could ameliorate cartilage erosion and degradation. These results collectively indicate that Dio can be used as a promising and effective agent for the therapy of OA.

**Supplementary Information:**

The online version contains supplementary material available at 10.1186/s12950-023-00339-w.

## Introduction

Osteoarthritis (OA) is a common chronic degenerative disease in elderly individuals and is characterized by the proliferation of subchondral bone and progressive articular cartilage degeneration [1]. The arthritis usually affects weight-bearing joints and joints with a lot of activity (knee and hip joints), resulting in persistent pain, limited mobility and physical disability [2, 3]. It has been reported that millions of people worldwide are afflicted by this disease, and this imposes a high medical burden to society [4]. However, there is currently no valid pharmacology treatment to reverse the progression of OA, and many patients with advanced disease have to undergo arthroplasty [5]. Therefore, the discovery of new therapeutic treatment options for OA is of great clinical significance.

Although the underlying mechanism leading to OA is complex, inflammation and inflammatory mediators have been proved to play a critical role in its progression [3, 6]. Various inflammatory mediators are known to be excessively released in the joints of OA patients [6, 7]. Specifically, interleukin-1β (IL-1β), the most widely studied pro-inflammatory cytokine, has been detected in the joint of OA patients [7, 8]. As reported in previous studies, IL-1β could increase the production of matrix metalloproteinases (MMPs) and aggrecanase-2 (ADAMTS-5) and suppress the synthesis of cartilage matrix in chondrocytes, leading to cartilage metabolism disorder [9–11]. Additionally, many studies have demonstrated that IL-1β can stimulate chondrocytes to generate cyclooxygenase-2 (COX2) and inducible nitric oxide synthase (iNOS), further promoting the release of prostaglandin E2 (PGE2) and nitric oxide (NO) [9, 11]. Accordingly, inhibiting the production of IL-1β-induced pro-inflammatory mediators and catabolic enzymes may provide a promising approach for the treatment of OA.

In recent years, research investigating the biological activities of plant compounds has been steadily increasing. Dioscin (Dio), a natural steroid saponin, can be extracted from various edible vegetables and herbs [12]. Previous studies have indicated that Dio has multiple pharmacological activities, including anti-oxidative [13], anti-apoptosis [14, 15], and anti-inflammatory [16, 17]. The research results of Wang et al. [18] suggested that Dio could attenuate IL-1β-activated inflammation and catabolic activity in human nucleus pulposus cells. In addition, Dio has been reported to have a protective effect against monosodium urate-initiated gouty arthritis by repressing the release of inflammatory cytokines and the activation of the NF-κB signaling pathway [19]. However, the detailed role of Dio in OA has not been explored. Thus, we aimed to explore the protective effects of Dio on IL-1β-induced chondrocytes and surgically established OA models in this study. Moreover, the potential mechanism of action was investigated.

## Materials and methods

### Chemicals and reagents

Recombinant rat IL-1β (501-RL) was purchased from R&D Systems (Minneapolis, MN, USA). Dioscin (N1723) was supplied by ApexBio Technology (Houston, TX, USA). Dioscin was dissolved in dimethyl sulfoxide (DMSO) for in vitro experiments. Dulbecco’s modified Eagle’s medium (DMEM/F12) and fetal bovine serum (FBS) were procured from Gibco (Grand Island, NY, USA). Antibodies specific for iNOS (ab15323), aggrecan (ab36861), and collagen II (ab188570) were obtained from Abcam (Cambridge, UK). Antibodies specific for P65 (#8242), phosphorylated P65 (P-P65) (#3033), IκBα (#4814), phosphorylated-IκBα (P-IκBα) (#2859), IKKβ (#8943), phosphorylated-IKKα/β (P-IKKα/β) (#2697), P38 (#8690), phosphorylated-p38 (p-p38) (#4511), ERK (#4695), phosphorylated-ERK (P-ERK) (#4370), JNK (#9252), and phosphorylated-JNK (P-JNK) (#4668) were procured from Cell Signaling Technology (Danvers, MA, USA). Antibodies specific for MMP1 (10,371–2-AP), MMP3 (17,873–1-AP), and MMP13 (18,165–1-AP) were supplied by Proteintech Group (Wuhan, China). Antibodies against GAPDH (BM1623) and ADAMTS5 (A02802-1) were procured from Boster (Wuhan, China).

### Chondrocyte isolation and culture

Primary chondrocytes were obtained from the bilateral knee joints of Sprague–Dawley rats (7-day-old). In brief, the bilateral joints of rats were separated and the surrounding muscles and ligaments were removed. Then, the cartilages extracted from the rat joints were minced into small pieces. The cartilage fragments were washed with sterile phosphate buffered solution (PBS) and incubated with 0.25% trypsin at 37 °C for 30 min. Trypsin was replaced with 0.2% collagenase II and digestion was continued at 37 °C for 6 h. The suspended cells were centrifuged at 1500 r/min for 5 min. Subsequently, the isolated chondrocytes were resuspended and cultured in complete medium (DMEM/F12 containing 10% FBS and 1% penicillin/streptomycin solution) at 37 °C and 5% CO_2_. To prevent the loss of the chondrocyte phenotype, two to three passages of chondrocytes were utilized for subsequent experiments.

### Cell viability assay

The cell counting kit-8 (CCK-8) assay (Beyotime, Nanjing, China) was conducted to determine cell proliferation and viability. In brief, 1 × 10^4^ chondrocytes per well were inoculated into a 96-well plate. After they adhered to the surface, chondrocytes were incubated with different concentrations of Dio (200, 400, 800, and 1600 ng/mL) or incubated with IL-1β (10 ng/mL) in combination with different concentrations of Dio (200, 400, 800, and 1600 ng/mL) for 24 h. Subsequently, 100 µL of culture medium containing 10 μL of CCK-8 solution was added into each well. Then the plate was incubated at 37° in the dark for 1 h. A spectrophotometric microplate reader (Bio-Rad, Hercules, CA, USA) was utilized to detect the absorbance of the cells at 450 nm.

### Griess assay and ELISA

To detect the levels of NO and PGE2, chondrocytes were exposed to IL-1β (10 ng/ml) with or without different concentrations of Dio. Then, the culture supernatant was collected for subsequent measurements. The NO level was determined by estimating the nitrite concentration based on Griess reaction with a NO content assay kit (Beyotime, Nanjing, China). The PGE2 level was detected with an ELISA kit (R&D Systems, MN, USA). All procedures followed the instructions of the manufacturer and all assays were performed in triplicate.

### Quantitative real-time polymerase chain reaction (qRT-PCR)

Quantitative real-time polymerase chain reaction (qRT-PCR) was applied in the study. Total ribonucleic acid (RNA) of the chondrocytes was extracted with a RNA isolation kit purchased from Omega (Guangzhou, China) based on the manufacturer’s protocol. Subsequently, a spectrophotometric microplate reader (Bio-Rad) was applied to determine the concentration of RNA molecules, and the ratio of A260/A280 was calculated to verify the purity of the total RNA. Subsequently, a cDNA synthesis kit (TOYOBO, Osaka, Japan) was utilized to reverse transcribe equal amounts of RNA (1 μg) to stable cDNA. The qRT-PCR reaction was conducted at 95 °C for 1 min, followed by 39 cycles at 95 °C for 15 s and 60 °C for 15 s. Relative expression of each target gene was normalized to that of GAPDH and analyzed using the 2-ΔΔCq method.

### Western blot analysis

The chondrocytes were lysed on ice in RIPA lysis buffer (Boster, Wuhan, China) to extract whole‑cell proteins. Protein concentrations were then measured using a BCA assay kit obtained from Boster. The proteins (25 µg of each protein sample) were separated using gel electrophoresis and transferred to polyvinylidene fluoride (PVDF) membranes (Millipore, Billerica, MA, USA). Then, the membranes were blocked in 5% skimmed milk for 1 h and incubated with primary antibodies at 4 °C overnight. The next day, the membranes were incubated with the corresponding secondary antibodies (1:10,000) for 1 h. Subsequently, the proteins were detected using a western electrochemiluminescence substrate kit (Thermo Fisher Scientific, Grand Island, NY, USA) and photographed using a Bio-Rad scanner system. Protein band intensity was quantified using Image Lab 5.1 software (Bio-Rad).

### Immunofluorescence staining

The chondrocytes were initially seeded into a 24-well plate and cultured for 24 h. Following stimulation with 10 ng/mL IL-1β in presence or absence of 800 ng/mL of Dio, the cells were fixed in 4% paraformaldehyde for 30 min and permeabilized with 0.1% Triton X-100 for 5 min. Then, the cells were blocked with 5% normal goat serum for 1 h. The cells were then incubated with primary antibodies against collagen II (1:300), aggrecan (1:300), and P65 (1:400) at 4 °C overnight. The next day, the chondrocytes were washed three times with PBS and incubated with FITC-conjugated (green)-anti-rabbit IgG antibody (Abcam, Cambridge, UK) for 1 h. The cells were then stained with 4,6-diamidino-2-phenylindole (DAPI) in PBS for 5 min. Finally, the images of the cells were photographed using a fluorescence microscope (Evos Flauto, Life Technologies, Carlsbad, CA, USA).

### Rat OA model establishment and grouping

Twenty-four male Sprague–Dawley rats (weight, 250–300 g) were obtained from the Laboratory Animal Center of Tongji Hospital. The rats were reared under controlled conditions with a 12-h dark/light cycle. All rats were maintained on a standard diet and water was made freely available. The animals were randomly divided into three groups: sham group (*n* = 8), OA group (*n* = 8), and Dio treatment group (*n* = 8). The rat OA model was built using the open surgical method as mentioned previously [20]. Briefly, after the right knee joint of rat was shaved and sterilized, a 1–2 cm incision was performed on the inside of the knee joint to expose joint cavity. Then, the anterior cruciate ligament was transected and partial medial meniscus was resected. Subsequently, joint capsule and skin were sutured with 4–0 non-absorbable suture. In the sham group, the rats received only skin and capsule incisions. After surgery, the rats in the Dio treatment group were fed 80 mg/kg/day Dio until they were sacrificed. Rats in the sham and OA groups received the vehicle as a control. This animal experiment was approved by the Institutional Animal Care and Use Committee of Tongji Hospital (TJH-202103005).

### Pain behaviors

Pain related behaviors were tested using paw withdrawal threshold (PWT) and weight-bearing asymmetry as described in the previous literature [21, 22]. PWT was applied to assessment mechanical allodynia. Briefly, the rats to be tested were placed in individual mesh cages. Von Frey filaments were applied starting in ascending order to the mid-plantar surface of the hind paw through the mesh floor. The withdrawal of the claw caused by the stimulus was recorded as a response. The weight-bearing asymmetry was determined by measuring changes in the weight distribution of the hind limbs using a hind limb weight device. Readings of the left and right hind limbs were recorded. Then, the formula [right limb weight / (left limb weight + right limb weight) × 100] was used to calculate the weight asymmetry percentage of the right hind limb. All animals were examined on the two different measure systems once a week from pre-surgery to eight weeks after surgery. All data used in the calculations were obtained from two independent observers.

### Histological and immunohistochemistry analysis

The rats were sacrificed at the eighth week after the operation, and the right knee joint was separated. After removing the muscles around the joints, all samples were fixed with 4% paraformaldehyde. The fixed samples were decalcified with 10% ethylenediamine tetraacetic acid (EDTA) and embedded in paraffin. Subsequently, the specimens were cut into sections with a thickness of 5 µm. Then, some sections were subjected to hematoxylin and eosin (H&E) and Safranin-O-Fast green staining. In addition, antibodies specific for iNOS, MMP13, collagen II, and aggrecan were used for immunohistochemical staining. The Osteoarthritis Research Society International (OARSI) scores [23] were introduced to assess the changes of chondrocytes and cartilage matrix degeneration in the sections from each group.

### Statistical analysis

The data are presented as mean ± standard deviation (SD). All data were obtained through at least three independent repeated experiments. The GraphPad Prism V.9.2 software (GraphPad Software Inc., La Jolla, CA, USA) was used for statistical analyses in this work. The differences between the two groups were determined with unpaired t-test, and one-way analysis of variance (ANOVA) followed by Tukey’s post hoc was applied for multiple comparisons. Statistical significance was set at *P* < 0.05.

## Results

### Effects of Dio on cell viability

**Fig. 1:**
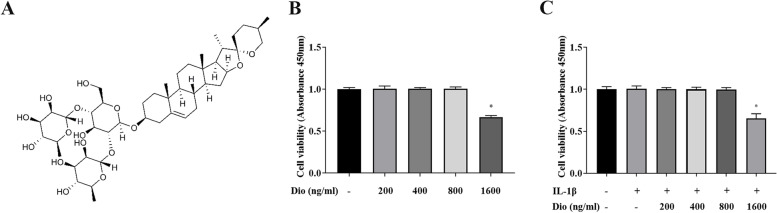
Effects of Dio on cell viability. **A** Chemical structure of Dio. **B** Rat chondrocytes were treated with different concentrations of Dio in the absence or (**C**) presence of IL-1β (10 ng/mL) for 24 h. The cell viability was evaluated using a CCK-8 kit..^*^P < 0.05 vs. control group; *n* = 3

The cytotoxic effect of Dio on chondrocytes was examined by CCK-8 assay. As presented in Fig. 1, treatment of chondrocytes with 200, 400, and 800 ng/mL of Dio, or incubation with IL-1β exhibited no cytotoxicity in primary chondrocytes. However, treatment with 1600 ng/mL of Dio decreased the viability of primary chondrocytes. Therefore, Dio was used at doses of 200, 400, and 800 ng/mL in subsequent studies.

### Dio attenuates IL-1β- stimulated production of NO, PGE_2_, COX-2 and iNOS

**Fig. 2:**
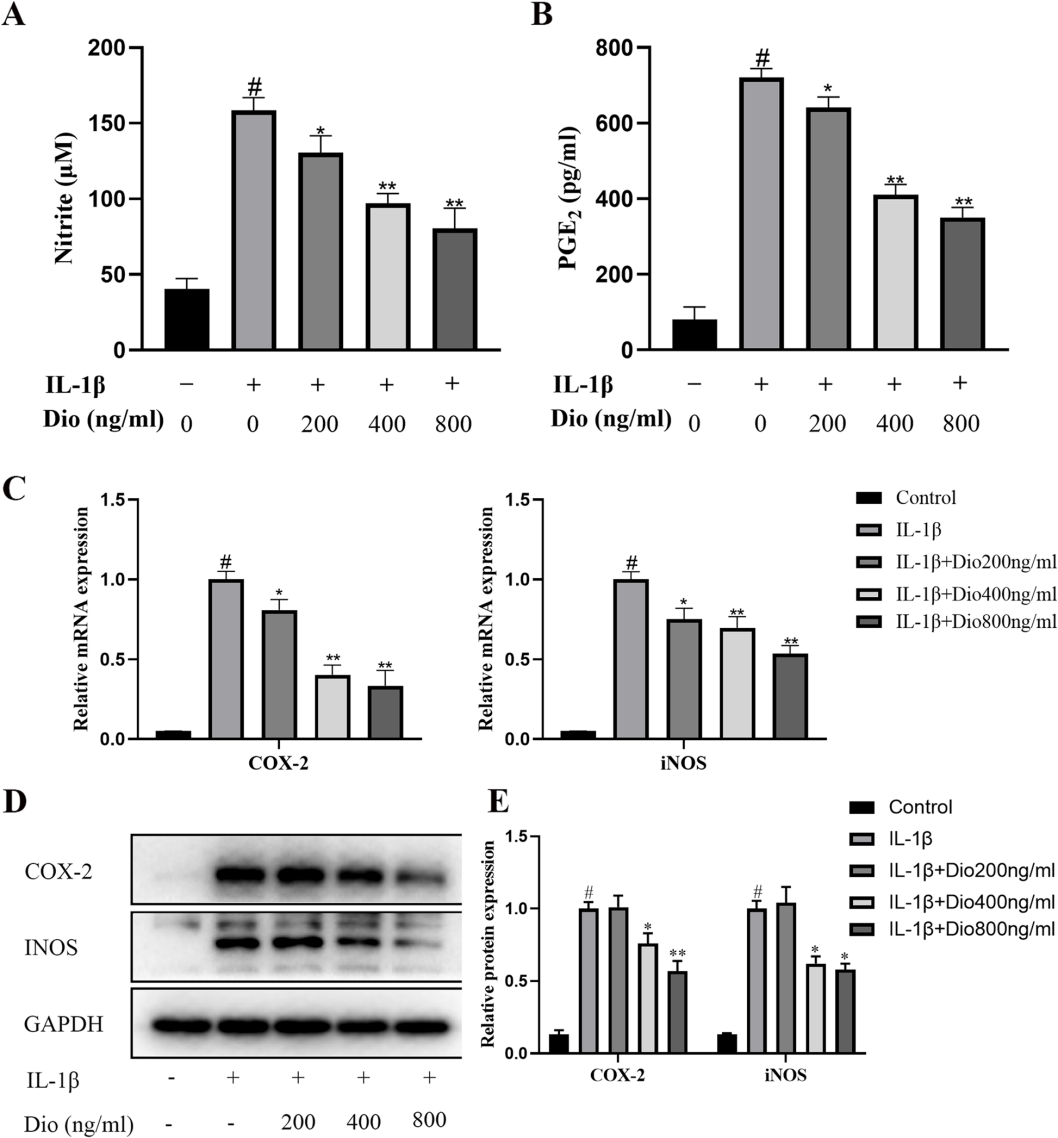
Dio suppresses IL-1β-induced inflammatory cytokines expression in chondrocytes. Chondrocytes were exposed to IL-1β (10 ng/mL) with or without Dio (200, 400, and 800 ng/mL) for 24 h. **A** Griess reaction was used to determine the NO concentration. **B** PGE2 level was detected by ELISA. **C** Gene expressions of COX-2 and iNOS were detected by qRT-PCR. **D** Representative western blots and (**E**) quantitative analysis of COX-2 and iNOS in each group. ^#^*P* < 0.05 vs. control group; ^*^P < 0.05 vs. IL-1β group; ^**^P < 0.01 vs. IL-1β group; *n* = 3

The release of excess inflammatory factors will promote the progression of OA. We used Griess reaction to measure the concentration of NO and ELISA kit to measure the PGE_2_ level in the supernatant of cell culture. Figure 2A and B indicate that treatment with IL-1β markedly upregulated NO and PGE_2_ production in chondrocytes. However, Dio alleviated these trends. Moreover, western blotting and qRT-PCR were used to detect whether Dio could inhibit the expression of COX-2 and iNOS. As exhibited in Fig. 2C-E, IL-1β markedly stimulated the production of iNOS and COX-2 in chondrocytes. However, Dio notably inhibited the release of iNOS and COX-2 at concentrations of 400 and 800 ng/mL. These changes were obvious at both the transcriptional and translational levels.

### Dio inhibits IL-1β-mediated upregulation of MMPs and ADAMTS-5

**Fig. 3:**
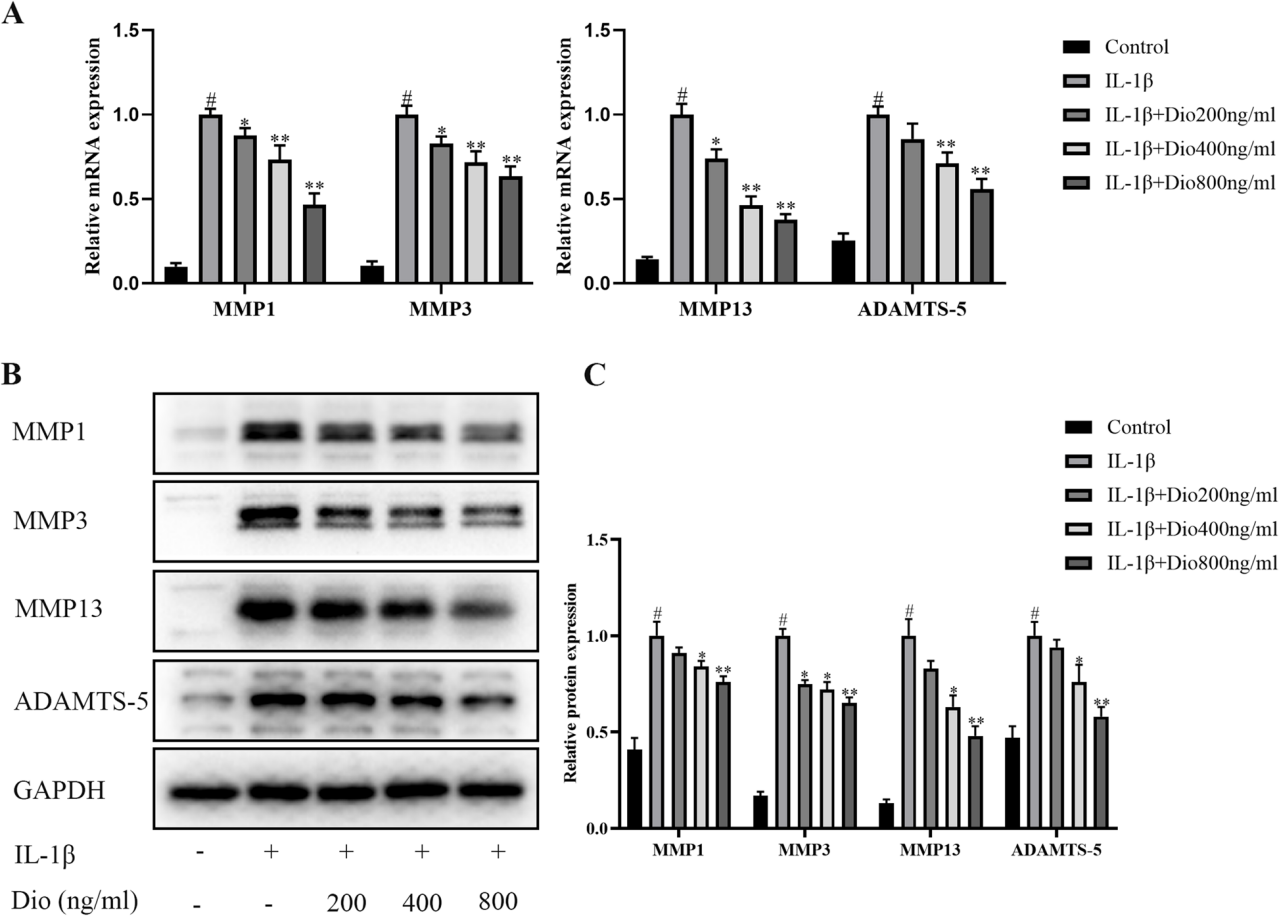
Dio inhibits IL-1β-induced upregulation of MMPs and ADAMTS-5. Chondrocytes were exposed to IL-1β (10 ng/mL) with or without Dio (200, 400, and 800 ng/mL) for 24 h. **A** Gene expressions of MMP1, MMP3, MMP13, and ADAMTS-5 were detected by qRT-PCR. **B** Representative western blots and (**C**) quantitative analysis of MMP1, MMP3, MMP13, and ADAMTS-5 in each group. ^#^*P* < 0.05 vs. control group; ^*^P < 0.05 vs. IL-1β group; ^**^P < 0.01 vs. IL-1β group; *n* = 3

The reduction of cartilage matrix promotes the progression of OA, which is caused by excessive cartilage matrix degrading enzymes, including MMPs (MMP1, MMP3, and MMP13) and ADAMTS5. As presented in Fig. 3, the mRNA and protein expression of MMP1, MMP3, MMP13, and ADAMTS-5 were notably increased in the IL-1β treatment group. However, Dio treatment significantly suppressed these changes.

### Dio ameliorates IL-1β-induced cartilage matrix degradation

**Fig. 4:**
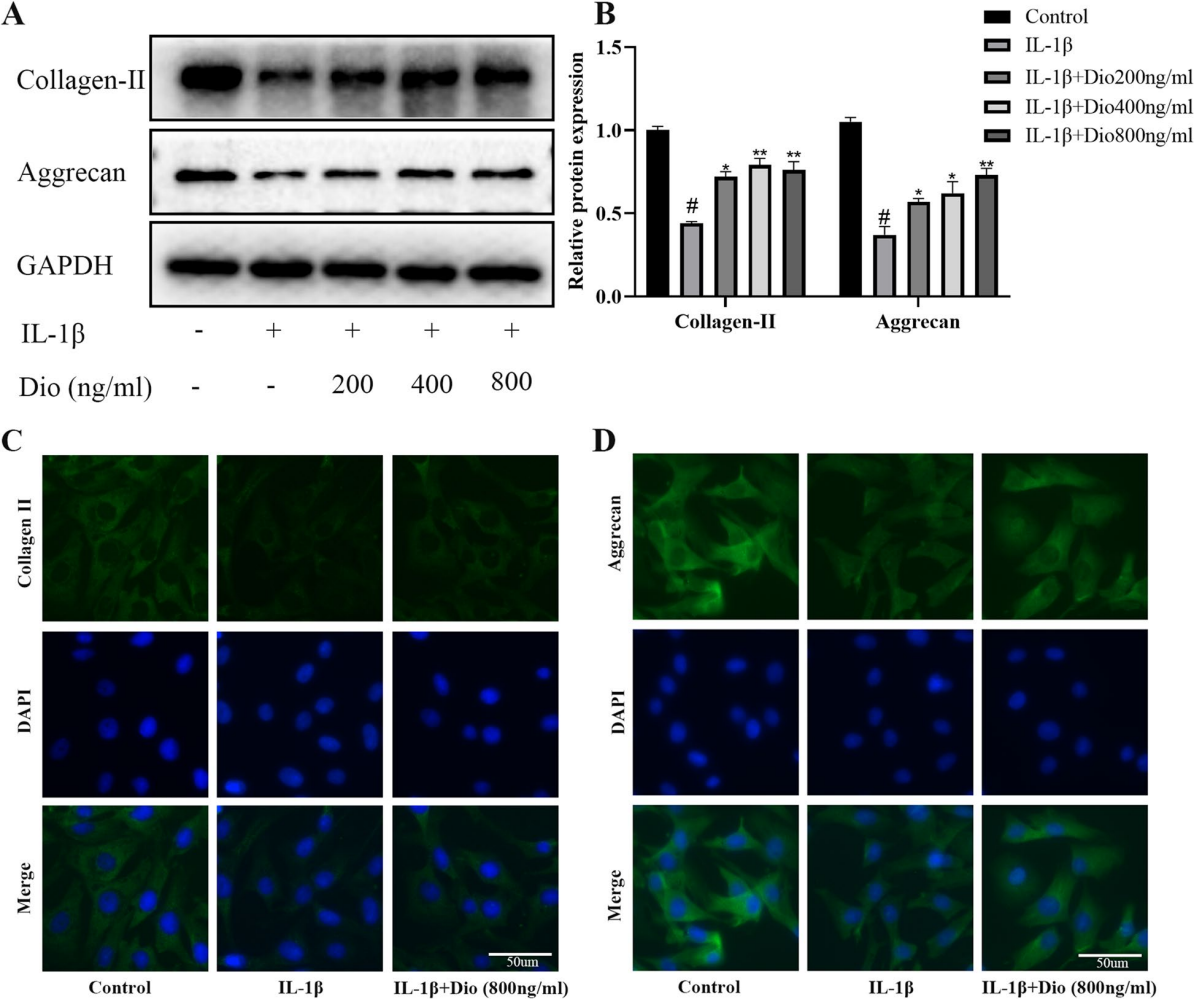
Dio ameliorates IL-1β-induced cartilage matrix degradation. Chondrocytes were exposed to IL-1β (10 ng/mL) with or without Dio (200, 400, and 800 ng/mL) for 24 h. **A** Representative western blots and (**B**) quantitative analysis of collagen II and aggrecan in each group. **C** Collagen II and (**D**) aggrecan were observed by immunofluorescence after the cells treated with IL-1β (10 ng/mL) in absence or presence of Dio (800 ng/mL). ^#^*P* < 0.05 vs. control group; ^*^P < 0.05 vs. IL-1β group;.^**^P < 0.01 vs. IL-1β group; *n* = 3

The collagen II and aggrecan are two vital cartilage matrix components synthesized by chondrocytes. To investigate whether Dio could alleviate the degradation of extracellular matrix in chondrocytes induced by IL-1β, western blotting and immunofluorescence staining were conducted. As exhibited in Fig. 4A and B, IL-1β significantly inhibited the synthesis of collagen II and aggrecan in chondrocytes. Dio reversed this change, and dramatically enhanced the production of collagen II and aggrecan. Moreover, immunofluorescence staining also showed that Dio (800 ng/ml) attenuated the downregulation of collagen II and aggrecan (Fig. 4C and D).

### Effects of Dio on MAPK and NF-κB signaling pathway activation

**Fig. 5:**
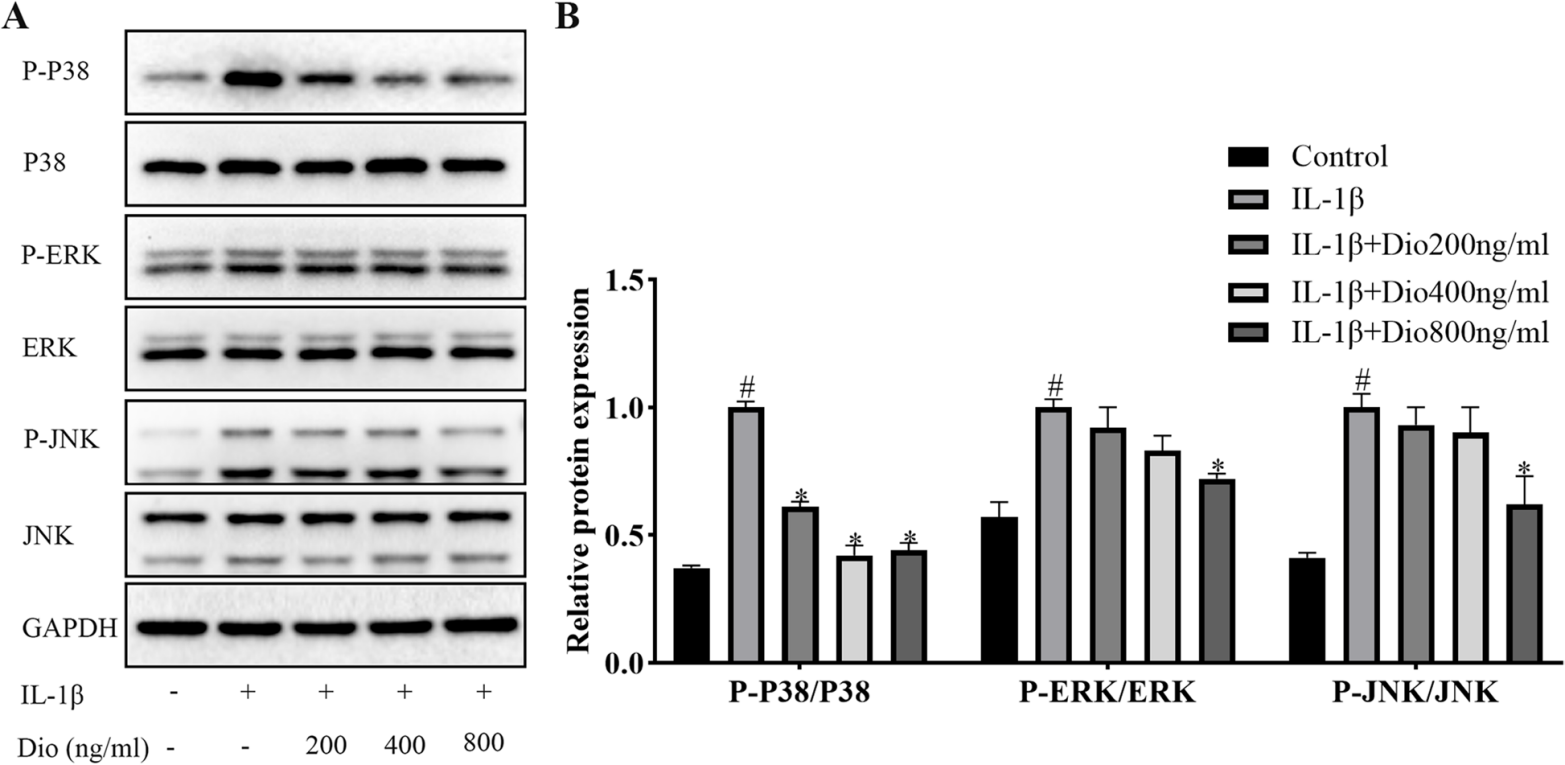
Dio blocks IL-1β-induced MAPK signaling pathway activation. Chondrocytes were exposed to IL-1β (10 ng/mL) with or without Dio (200, 400, and 800 ng/mL) for 30 min. **A** Representative western blots and (**B**) quantitative analysis of P38, P-P38, ERK, P-ERK, JNK, and P-JNK in each group. ^#^*P* < 0.05 vs. control group; ^*^P < 0.05 vs. IL-1β group; ^**^P < 0.01 vs. IL-1β group; *n* = 3

**Fig. 6:**
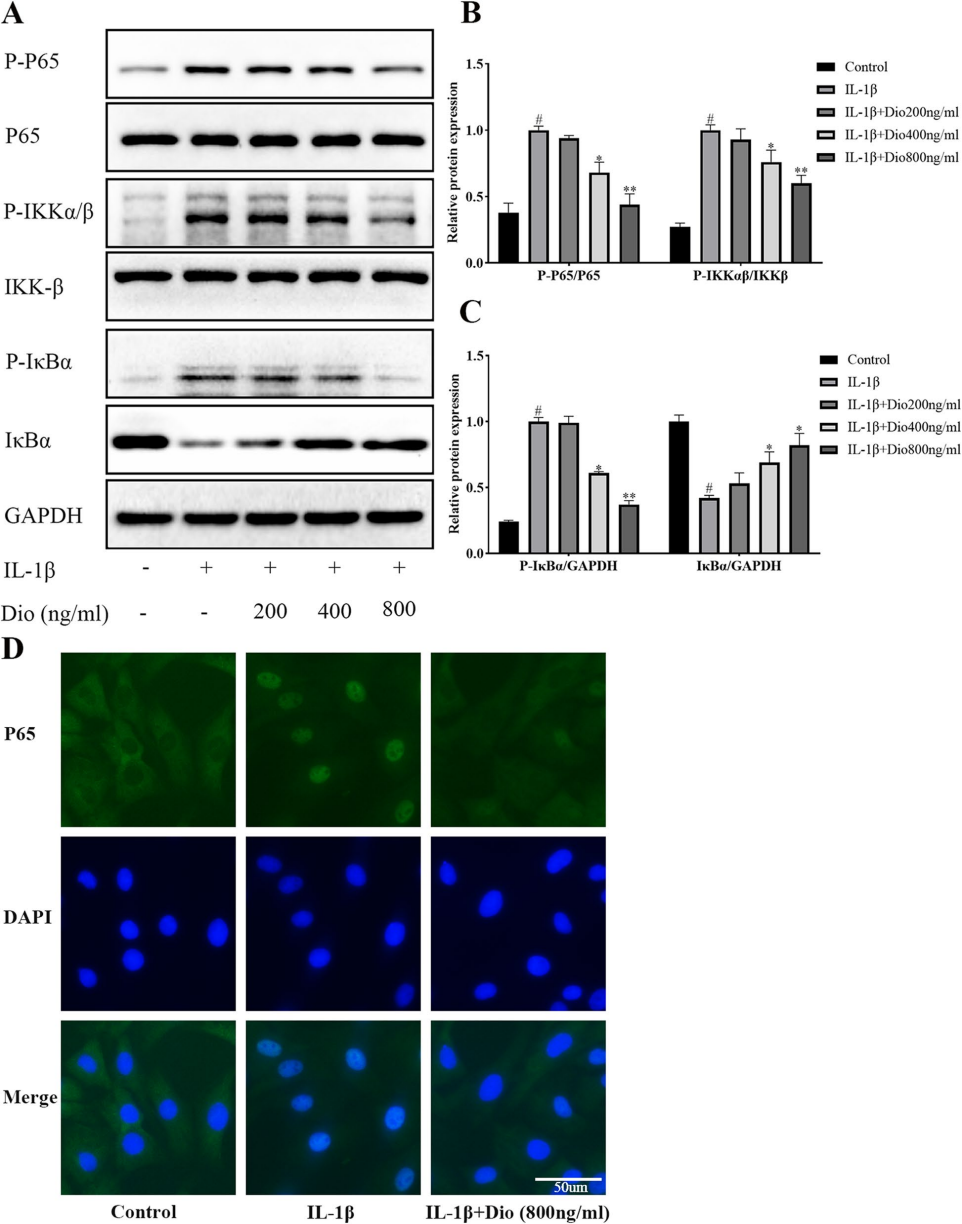
Dio inhibits IL-1β-mediated activation of NF-κB signaling pathway. Chondrocytes were exposed to IL-1β (10 ng/mL) with or without Dio (200, 400, and 800 ng/mL) for 30 min. **A** Representative western blots and (**B** and** C**) quantitative analysis of P65, P-P65, IKK-β, P- IKKα/β, IκBα and P- IκBα in each group. **D** The nuclear translocation of P65 was observed by immunofluorescence after the cells treated with IL-1β (10 ng/mL) in absence or presence of Dio (800 ng/mL). ^#^*P* < 0.05 vs. control group; ^*^P < 0.05 vs. IL-1β group; ^**^P < 0.01 vs. IL-1β group; *n* = 3

To explore the underlying molecular mechanism of the protective effects of Dio, we tested the MAPK and NF-κB signaling pathways after rat chondrocytes were stimulated with IL-1β in presence or absence of various concentrations of Dio. As presented in Fig. 5, IL-1β notably stimulated the expression of P-P38, P-ERK, and P-JNK, which indicated the activation of MAPK signaling pathway. However, the high concentration (800 ng/ml) of Dio reduced the phosphorylation levels of the three MAPK signaling molecules induced by IL-1β. Low concentrations of Dio (200 and 400 ng/ml) mitigated the expression of these signaling molecules, but there was only a statistical difference for P-P38. Similarly, the results shown in Fig. 6A and B suggested that Dio significantly decreased the phosphorylation levels of signaling molecules (P65, IKKα/β, and IκBα) in NF-κB pathway. Moreover, immunofluorescence staining showed that Dio blocked IL-1β-induced translocation of P65. As exhibited in Fig. 6C, P65 was distributed in the cytoplasm of untreated chondrocytes. After IL-1β stimulation, P65 obviously accumulated in the chondrocyte nucleus. However, Dio was found to suppress this process.

### Effects of Dio on the rat pain behaviors

**Fig. 7:**
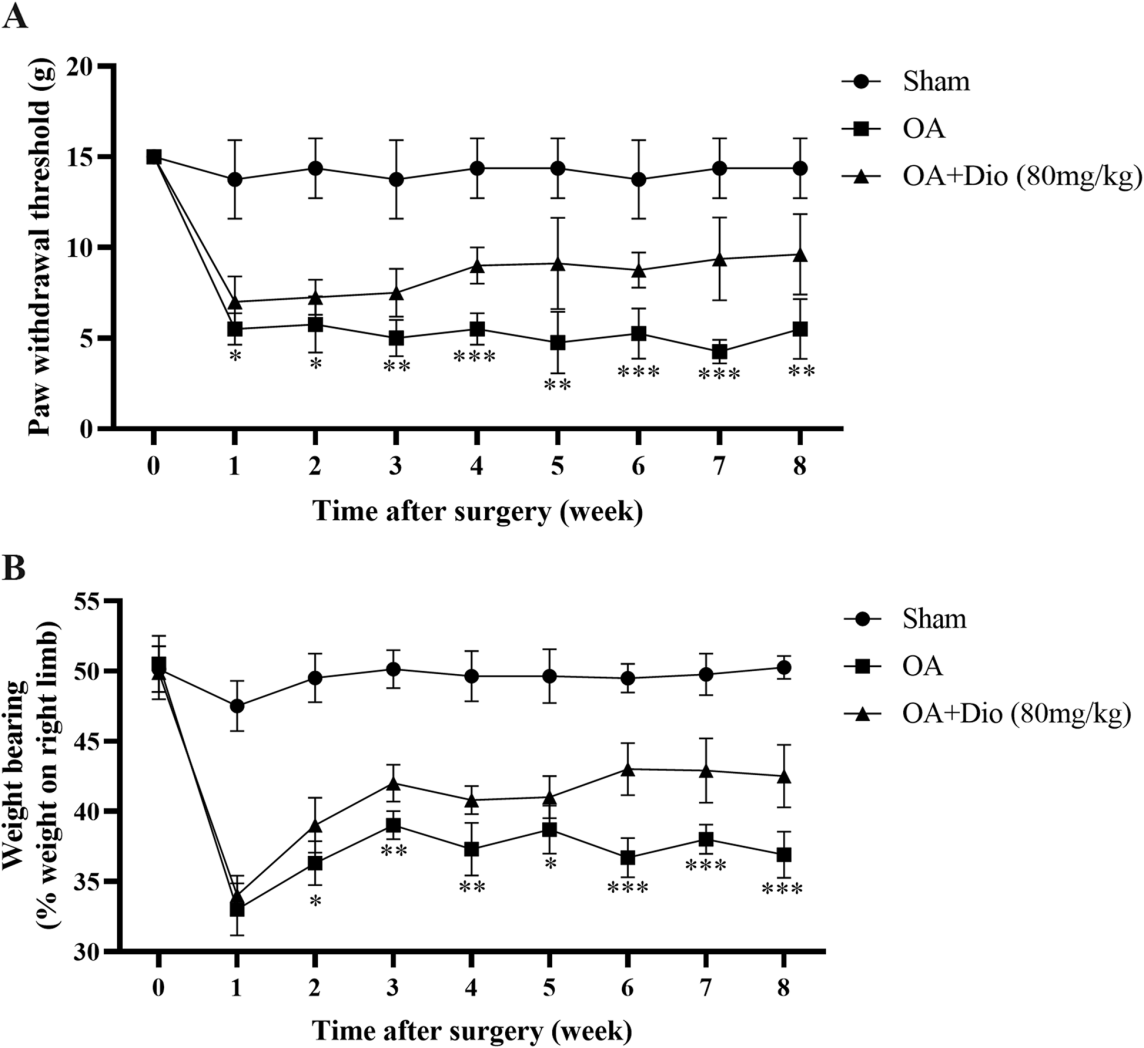
Effects of Dio on pain-related behaviors in rat OA models. **A** Evolution of paw withdrawal threshold (PWT) over time in all groups (*n* = 8 rats/group) from Week 0 (before surgery) to Week 8. **B** Evolution of Weight-bearing asymmetry over time in all groups (*n* = 8 rats/group) from Week 0 (before surgery) to Week 8. ^*^P < 0.05 vs. OA group; ^**^P < 0.01 vs. OA group; ^***^P < 0.001 vs. OA group

Joint pain is the most common clinical symptom and primary therapeutic goal for OA patients. Therefore, we analyzed the impacts of Dio on OA-induced joint pain behaviors. As shown in Fig. 7A, the PWT of surgically induced rat OA models was significantly decreased beginning at week 1 and persisting till to week 8 compared with the rats in the sham group. Conversely, no significant mechanical allodynia was observed in sham group during the study period. However, mechanical allodynia was remarkably upregulated by administration of Dio. As illustrated in Fig. 7B, we observed that weight-bearing on the right hind limb was significantly decreased at week 1 in all groups. The weight-bearing on the right hind limb of Dio treated rats was significantly increased beginning at week 2 compared with OA rat models. These results indicated that treatment with Dio successfully alleviate pain-related behaviors in rat OA models.

### Dio ameliorated the cartilage damage and inhibited inflammation in rat OA models

**Fig. 8:**
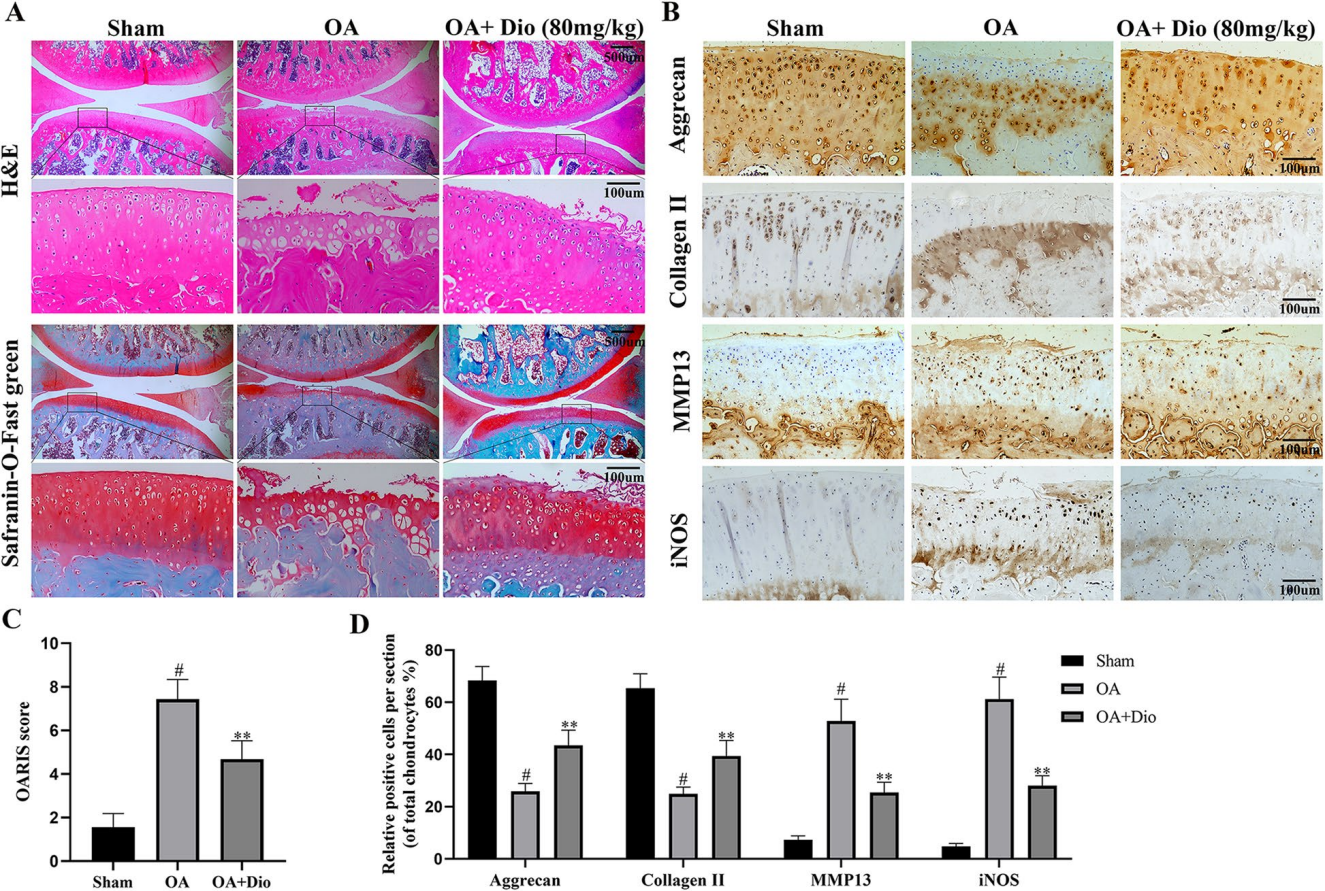
Dio alleviated osteoarthritis progression in rat OA models. **A** The H&E and Safranin-O-Fast green staining demonstrated that the OA group showed obvious damage to the cartilage compared with the sham group. However, the administration of Dio significantly ameliorated the cartilage damage compared with the OA group. **B** The immunohistochemical staining of collagen II, aggrecan, MMP13 and iNOS also confirmed the positive effects of Dio on cartilage. **C** The OARSI scores of each group. **D** Quantitative of collagen II, aggrecan, MMP13, and iNOS in the cartilage samples from each group. ^#^*P* < 0.05 vs. sham group; ^**^P < 0.01 vs. OA group

The morphological differences in the cartilage of the specimens were observed by H&E and Safranin-O-fast green staining. As present in Fig. 8A, the OA group showed apparent cartilage surface erosion and chondrocyte diminution, in comparison with the smooth cartilage surface and normal arrangement of chondrocytes in the sham group. However, the administration of Dio significantly ameliorated cartilage damage, in comparison to the OA group. The immunohistochemical staining results of collagen II, aggrecan, MMP13, and iNOS also suggested that Dio has a positive effect on cartilage (Fig. 8B). Further, OARSI scores were obtained for all three groups. As exhibited in Fig. 8C, the Dio treatment group showed lower OARSI scores than the OA group, which indicated that Dio could alleviate the progression of OA. In addition, the quantitative analysis results indicated that cartilage matrix components including collagen II and aggrecan significantly decreased in OA group, but these were alleviated in Dio treatment group. Compared with the sham group, the number of MMP13 and iNOS positive cells in the OA group increased, while the number of MMP13 and iNOS positive cells decreased after Dio treatment (Fig. 8D).

## Discussion

Osteoarthritis is a common joint disease and seriously affects the quality of life in the elderly. At present, some therapeutic options, including nonsteroidal anti-inflammatory drugs (NSAIDs), glucocorticoids, and hyaluronic acid are available for the treatment of OA [24]; however, they are unable to inhibit the progression of OA and may cause cardiovascular, renal, and gastrointestinal side effects [25]. Recently, many studies have focused on the application of natural plant extracts in the treatment of OA because of their potential anti-inflammatory properties and reduced risk of adverse events [26]. Dio has been demonstrated to inhibit the release of inflammatory cytokines in mouse and rat models of various diseases, such as dimethylnitrosamine-induced acute liver injury [27], intestinal ischemia/reperfusion injury [28], and lipopolysaccharide-induced acute lung injury [16]. In addition, Dio also showed a protective effect in chronic inflammatory diseases including gouty arthritis[19], silicosis [29], and atherosclerosis [14]. In this study, the protective effects of Dio were demonstrated in vitro using primary chondrocytes, and further confirmed in a rat OA model.

IL-1β is a major pro-inflammatory cytokine that participates in the pathological process of OA by inducing inflammation. There is increasing evidence that the production of iNOS and COX-2 is upregulated by IL-1β during OA progression [11, 30]. Moreover, iNOS and COX-2 promote the release of NO and PGE2, which are well-known inflammatory mediators [31]. In this study, our results indicated that the production of NO, PGE_2_, iNOS and COX-2 was significantly increased after incubation with IL-1β. However, Dio reduced the IL-1β-upregulated expression levels of NO, PGE_2_, iNOS and COX-2. This was consistent with the findings of a previous study which indicated that Dio could markedly inhibit the production of iNOS and COX-2 in lipopolysaccharide-induced mouse mammary epithelial cells [32]. Furthermore, Dio has been reported to inhibit the expression of iNOS and COX-2 in IL-1β-induced human osteoarthritis chondrocytes [33]. Therefore, Dio may exert its anti-inflammatory effects by repressing the synthesis of iNOS and COX-2.

Under normal circumstances, the anabolism and catabolism of cartilage are dynamic. Dysfunction in cartilage metabolism is key to the development of OA. Collagen II and aggrecan are the two main constituents of the extracellular matrix in cartilage, while MMPs and ADAMTS5 are the two catabolic enzymes responsible for the degradation of collagen II and Aggrecan, respectively [34, 35]. Excessive release of IL-1β in joints can disrupt this balance. In the study, our findings indicated that IL-1β dramatically increased the expression of the catabolic enzymes and downregulated the synthesis of collagen II and aggrecan in chondrocytes. However, the application of Dio repressed the overexpression of MMPs (MMP1, MMP3, and MMP13) and ADAMTS5 induced by IL-1β and improved the production of collagen II and aggrecan, which contribute to the maintenance of chondrocyte matrix homeostasis. Thus, the results revealed that Dio could alleviate OA progression through an anti-catabolic mechanism.

**Fig. 9:**
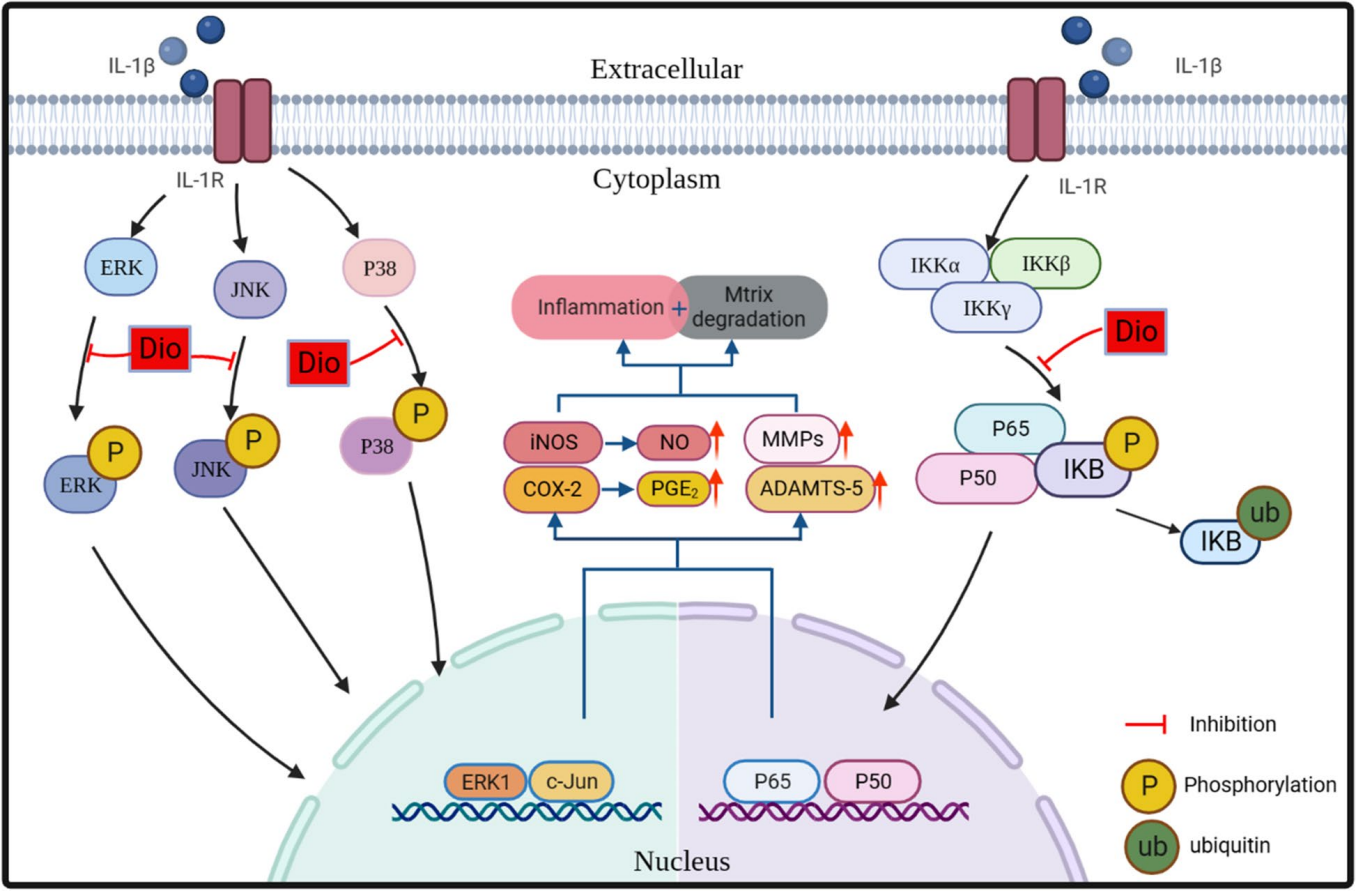
Schematic diagram of the protective effects of Dio on OA. IL-1β is an inflammatory cytokine which is excessive released in the joints of OA patients. The cytokine can induce the expression of pro-inflammatory factors (iNOS, COX2, MMPs and ADAMTS5) and downregulate the synthesis of Collagen II and Aggrecan in chondrocytes. Moreover, IL-1β functions by activating the MAPK and NF-κB signaling pathways. Dio can block IL-1β-induced MAPK and NF-κB signaling pathway activation, resulting in the inhibition of IL-1β-induced overexpression of MMPs and ADAMTS-5 and the improvement of the production of Collagen II and Aggrecan, which contribute to the maintenance of chondrocyte matrix homeostasis

It has been previously demonstrated that MAPK and NF-κB signaling pathways are involved in the progression of OA [36, 37]. The decisive role of MAPK and NF-κB signaling pathways in OA onset and progression provides evidence that intervention of these signaling pathways might have beneficial therapeutic effects. Drugs that have been clinically applied in the treatment of OA, including NSAIDs and glucocorticoids, are pharmacologically active compounds that can block the NF-κB signaling pathway [38]. Therefore, in this research, we focused on examining these pathways. IL-1β has been shown to activate all three MAPK signaling molecules (P38, ERK, JNK) and NF-κB signaling pathways, and induces the production of catabolic factors to further promote the destruction of the extracellular matrix [39]. Our results corroborated the activity of IL-1β described previously and demonstrated that Dio could partly inhibit IL-1β-induced activation of MAPK and NF-κB signaling pathways. This was consistent with the anti-inflammatory mechanism of Dio described in previous studies [18, 19, 28, 32]. Therefore, the protective effects of Dio on OA may be related to the inhibition of the MAPK and NF-κB pathways (Fig. 9).

To further demonstrate the protective effect of Dio against OA in vivo, we established an OA rat model. Clinically, ECM degeneration leads to progressive damage to the articular cartilage and is usually accompanied by pain. We used PWT and weight-bearing asymmetry to assess pain behaviors in rat OA models. The findings demonstrated that Dio could significantly alleviate pain-related behaviors in rat OA models. Moreover, the in vivo results indicated that Dio could alleviate joint degeneration in OA rat models. The images of H&E and Safranin-O-Fast green staining showed that characteristics of OA, such as cartilage surface erosion, chondrocyte diminution, and cartilage matrix loss, were significantly reversed by Dio. Similarly, Lu et al. [40] demonstrated that Dio has a protective effect on sodium iodoacetate induced cartilage destruction in rats. Moreover, immunohistochemical staining analysis further confirmed the anti-inflammatory and anti-catabolic effects of Dio, which are consistent with the trends observed in the in vitro studies. In addition, the OARSI score in rat OA models was significantly reduced after Dio treatment. Thus, taken together, our results strongly suggest that Dio can ameliorate the progression and reduce pain of OA.

This research is not without limitation. Firstly, the most effective dose of Dio in vivo application is unclear and needs further evaluation. However, a 90-day subchronic toxicological evaluation in Sprague–Dawley rats indicated that Dio was safe for potential clinical application [41]. Moreover, the dioscin-containing DA-9801 completed its Phase II clinical trial in the United States for the treatment of diabetic neuropathy [42], indicating that Dio is potentially safe for therapeutic use. In addition, the results demonstrate that Dio has an anti-inflammatory effect and reduce pain in vivo. The specific target and mechanism in detail of Dio action in vivo need to be further elucidated in the future experiments.

## Conclusions

In summary, our research provides new insights into the protective effects of Dio against OA. Dio exerts anti-inflammatory and anti-catabolic effects in the progression of OA. Moreover, the underlying mechanism of Dio action includes the inhibition of the MAPK and NF-κB signaling pathways. In addition, an in vivo study revealed that Dio could ameliorate cartilage degradation and alleviate pain. These findings indicate that Dio is an effective therapeutic agent for the treatment of OA.

## Supplementary Information

**Additional file 1. **

## Data Availability

All data included in this study are available from the corresponding author on reasonable request.

## References

[1] Silverwood V, Blagojevic-Bucknall M, Jinks C, Jordan JL, Protheroe J, Jordan KP (2015). Current evidence on risk factors for knee osteoarthritis in older adults: a systematic review and meta-analysis. Osteoarthritis Cartilage.

[2] Aicher WK, Rolauffs B (2014). The spatial organisation of joint surface chondrocytes: review of its potential roles in tissue functioning, disease and early, preclinical diagnosis of osteoarthritis. Ann Rheum Dis.

[3] Abramoff B, Caldera FE (2020). Osteoarthritis: Pathology, Diagnosis, and Treatment Options. Med Clin North Am.

[4] Samvelyan HJ, Hughes D, Stevens C, Staines KA (2021). Models of Osteoarthritis: Relevance and New Insights. Calcif Tissue Int.

[5] Latourte A, Kloppenburg M, Richette P (2020). Emerging pharmaceutical therapies for osteoarthritis. Nat Rev Rheumatol.

[6] Molnar V, Matisic V, Kodvanj I, Bjelica R, Jelec Z, Hudetz D, Rod E, Cukelj F, Vrdoljak T, Vidovic D, Staresinic M, Sabalic S, Dobricic B, Petrovic T, Anticevic D, Boric I, Kosir R, Zmrzljak UP and Primorac D. Cytokines and Chemokines Involved in Osteoarthritis Pathogenesis. Int J Mol Sci 22. 2021. 10.3390/ijms22179208. 10.3390/ijms22179208 PMC8431625 34502117

[7] Poulet B, Staines KA (2016). New developments in osteoarthritis and cartilage biology. Curr Opin Pharmacol.

[8] de Lange-Brokaar BJ, Ioan-Facsinay A, van Osch GJ, Zuurmond AM, Schoones J, Toes RE, Huizinga TW, Kloppenburg M (2012). Synovial inflammation, immune cells and their cytokines in osteoarthritis: a review. Osteoarthritis Cartilage.

[9] Tu C, Ma Y, Song M, Yan J, Xiao Y, Wu H (2019). Liquiritigenin inhibits IL-1beta-induced inflammation and cartilage matrix degradation in rat chondrocytes. Eur J Pharmacol..

[10] Verma P, Dalal K (2011). ADAMTS-4 and ADAMTS-5: key enzymes in osteoarthritis. J Cell Biochem.

[11] Kapoor M, Martel-Pelletier J, Lajeunesse D, Pelletier JP, Fahmi H (2011). Role of proinflammatory cytokines in the pathophysiology of osteoarthritis. Nat Rev Rheumatol.

[12] Yang L, Ren S, Xu F, Ma Z, Liu X, Wang L (2019). Recent Advances in the Pharmacological Activities of Dioscin. Biomed Res Int.

[13] Zhao L, Tao X, Qi Y, Xu L, Yin L, Peng J (2018). Protective effect of dioscin against doxorubicin-induced cardiotoxicity via adjusting microRNA-140-5p-mediated myocardial oxidative stress. Redox Biol.

[14] Yang Q, Wang C, Jin Y, Ma X, Xie T, Wang J, Liu K, Sun H (2019). Disocin prevents postmenopausal atherosclerosis in ovariectomized LDLR-/- mice through a PGC-1alpha/ERalpha pathway leading to promotion of autophagy and inhibition of oxidative stress, inflammation and apoptosis. Pharmacol Res.

[15] Yao H, Xu Y, Yin L, Tao X, Xu L, Qi Y, Han X, Sun P, Liu K, Peng J (2017). Dioscin Protects ANIT-Induced Intrahepatic Cholestasis Through Regulating Transporters. Apoptosis Oxidative Stress Front Pharmacol.

[16] Yao H, Sun Y, Song S, Qi Y, Tao X, Xu L, Yin L, Han X, Xu Y, Li H, Sun H, Peng J (2017). Protective Effects of Dioscin against Lipopolysaccharide-Induced Acute Lung Injury through Inhibition of Oxidative Stress and Inflammation. Front Pharmacol.

[17] Yang R, Chen W, Lu Y, Li Y, Du H, Gao S, Dong X, Yuan H (2017). Dioscin relieves endotoxemia induced acute neuro-inflammation and protect neurogenesis via improving 5-HT metabolism. Sci Rep.

[18] Wang L, Gu Y, Zhao H, Chen R, Chen W, Qi H, Gao W (2020). Dioscin Attenuates Interleukin 1beta (IL-1beta)-Induced Catabolism and Apoptosis via Modulating the Toll-Like Receptor 4 (TLR4)/Nuclear Factor kappa B (NF-kappaB) Signaling in Human Nucleus Pulposus Cells. Med Sci Monit.

[19] Han J, Shi G, Li W, Xie Y, Li F, Jiang D (2021). Preventive effect of dioscin against monosodium urate-mediated gouty arthritis through inhibiting inflammasome NLRP3 and TLR4/NF-kappaB signaling pathway activation: an in vivo and in vitro study. J Nat Med.

[20] Appleton CT, McErlain DD, Pitelka V, Schwartz N, Bernier SM, Henry JL, Holdsworth DW, Beier F (2007). Forced mobilization accelerates pathogenesis: characterization of a preclinical surgical model of osteoarthritis. Arthritis Res Ther.

[21] Gao SJ, Li DY, Liu DQ, Sun J, Zhang LQ, Wu JY, Song FH, Zhou YQ, Mei W (2022). Dimethyl Fumarate Attenuates Pain Behaviors in Osteoarthritis Rats via Induction of Nrf2-Mediated Mitochondrial Biogenesis. Mol Pain.

[22] Piel MJ, Kroin JS, van Wijnen AJ, Kc R, Im HJ (2014). Pain assessment in animal models of osteoarthritis. Gene.

[23] Pritzker KP, Gay S, Jimenez SA, Ostergaard K, Pelletier JP, Revell PA, Salter D, van den Berg WB (2006). Osteoarthritis cartilage histopathology: grading and staging. Osteoarthritis Cartilage.

[24] Richards MM, Maxwell JS, Weng L, Angelos MG, Golzarian J (2016). Intra-articular treatment of knee osteoarthritis: from anti-inflammatories to products of regenerative medicine. Phys Sportsmed.

[25] Katz JA (2013). COX-2 inhibition: what we learned–a controversial update on safety data. Pain Med.

[26] Dragos D, Gilca M, Gaman L, Vlad A, Iosif L, Stoian I and Lupescu O. Phytomedicine in Joint Disorders. Nutrients. 2017;9. 10.3390/nu9010070. 10.3390/nu9010070 PMC5295114 28275210

[27] Zhang W, Yin L, Tao X, Xu L, Zheng L, Han X, Xu Y, Wang C, Peng J (2016). Dioscin alleviates dimethylnitrosamine-induced acute liver injury through regulating apoptosis, oxidative stress and inflammation. Environ Toxicol Pharmacol.

[28] Zheng L, Han X, Hu Y, Zhao X, Yin L, Xu L, Qi Y, Xu Y, Han X, Liu K, Peng J (2019). Dioscin ameliorates intestinal ischemia/reperfusion injury via adjusting miR-351-5p/MAPK13-mediated inflammation and apoptosis. Pharmacol Res.

[29] Du S, Li C, Lu Y, Lei X, Zhang Y, Li S, Liu F, Chen Y, Weng D, Chen J (2019). Dioscin Alleviates Crystalline Silica-Induced Pulmonary Inflammation and Fibrosis through Promoting Alveolar Macrophage Autophagy. Theranostics.

[30] Lei M, Wang JG, Xiao DM, Fan M, Wang DP, Xiong JY, Chen Y, Ding Y, Liu SL (2012). Resveratrol inhibits interleukin 1beta-mediated inducible nitric oxide synthase expression in articular chondrocytes by activating SIRT1 and thereby suppressing nuclear factor-kappaB activity. Eur J Pharmacol.

[31] Dumond H, Presle N, Pottie P, Pacquelet S, Terlain B, Netter P, Gepstein A, Livne E, Jouzeau JY (2004). Site specific changes in gene expression and cartilage metabolism during early experimental osteoarthritis. Osteoarthritis Cartilage.

[32] Ran X, Yan Z, Yang Y, Hu G, Liu J, Hou S, Guo W, Kan X, Fu S (2020). Dioscin Improves Pyroptosis in LPS-Induced Mice Mastitis by Activating AMPK/Nrf2 and Inhibiting the NF-kappaB Signaling Pathway. Oxid Med Cell Longev.

[33] Wang H, Zhu H, Yang X (2020). Dioscin exhibits anti-inflammatory effects in IL-1beta-stimulated human osteoarthritis chondrocytes by activating LXRalpha. Immunopharmacol Immunotoxicol.

[34] Stanton H, Rogerson FM, East CJ, Golub SB, Lawlor KE, Meeker CT, Little CB, Last K, Farmer PJ, Campbell IK, Fourie AM, Fosang AJ (2005). ADAMTS5 is the major aggrecanase in mouse cartilage in vivo and in vitro. Nature.

[35] Sondergaard BC, Henriksen K, Wulf H, Oestergaard S, Schurigt U, Brauer R, Danielsen I, Christiansen C, Qvist P, Karsdal MA (2006). Relative contribution of matrix metalloprotease and cysteine protease activities to cytokine-stimulated articular cartilage degradation. Osteoarthritis Cartilage.

[36] Rigoglou S, Papavassiliou AG (2013). The NF-kappaB signalling pathway in osteoarthritis. Int J Biochem Cell Biol.

[37] Saklatvala J (2007). Inflammatory signaling in cartilage: MAPK and NF-kappaB pathways in chondrocytes and the use of inhibitors for research into pathogenesis and therapy of osteoarthritis. Curr Drug Targets.

[38] Niederberger E, Geisslinger G (2008). The IKK-NF-kappaB pathway: a source for novel molecular drug targets in pain therapy?. FASEB J.

[39] Jenei-Lanzl Z, Meurer A, Zaucke F (2019). Interleukin-1beta signaling in osteoarthritis - chondrocytes in focus. Cell Signal.

[40] Lu J, Zhang T, Sun H, Wang S, Liu M (2018). Protective effects of dioscin against cartilage destruction in a monosodium iodoacetate (MIA)-indcued osteoarthritis rat model. Biomed Pharmacother.

[41] Xu T, Zhang S, Zheng L, Yin L, Xu L, Peng J (2012). A 90-day subchronic toxicological assessment of dioscin, a natural steroid saponin, in Sprague-Dawley rats. Food Chem Toxicol.

[42] Kang KB, Ryu J, Cho Y, Choi SZ, Son M, Sung SH (2017). Combined Application of UHPLC-QTOF/MS, HPLC-ELSD and (1) H-NMR Spectroscopy for Quality Assessment of DA-9801, A Standardised Dioscorea Extract. Phytochem Anal.

