# Supplementary material for: Dioscin alleviates the progression of osteoarthritis: an in vitro and in vivo study
Source: J Inflamm (Lond). 2023 Apr 13;20:14. doi: 10.1186/s12950-023-00339-w (PMC10100120; doi:10.1186/s12950-023-00339-w)
Supplement: Supplementary file 1 — Additional file 1. [file 12950_2023_339_MOESM1_ESM.docx]

**Figure 2**


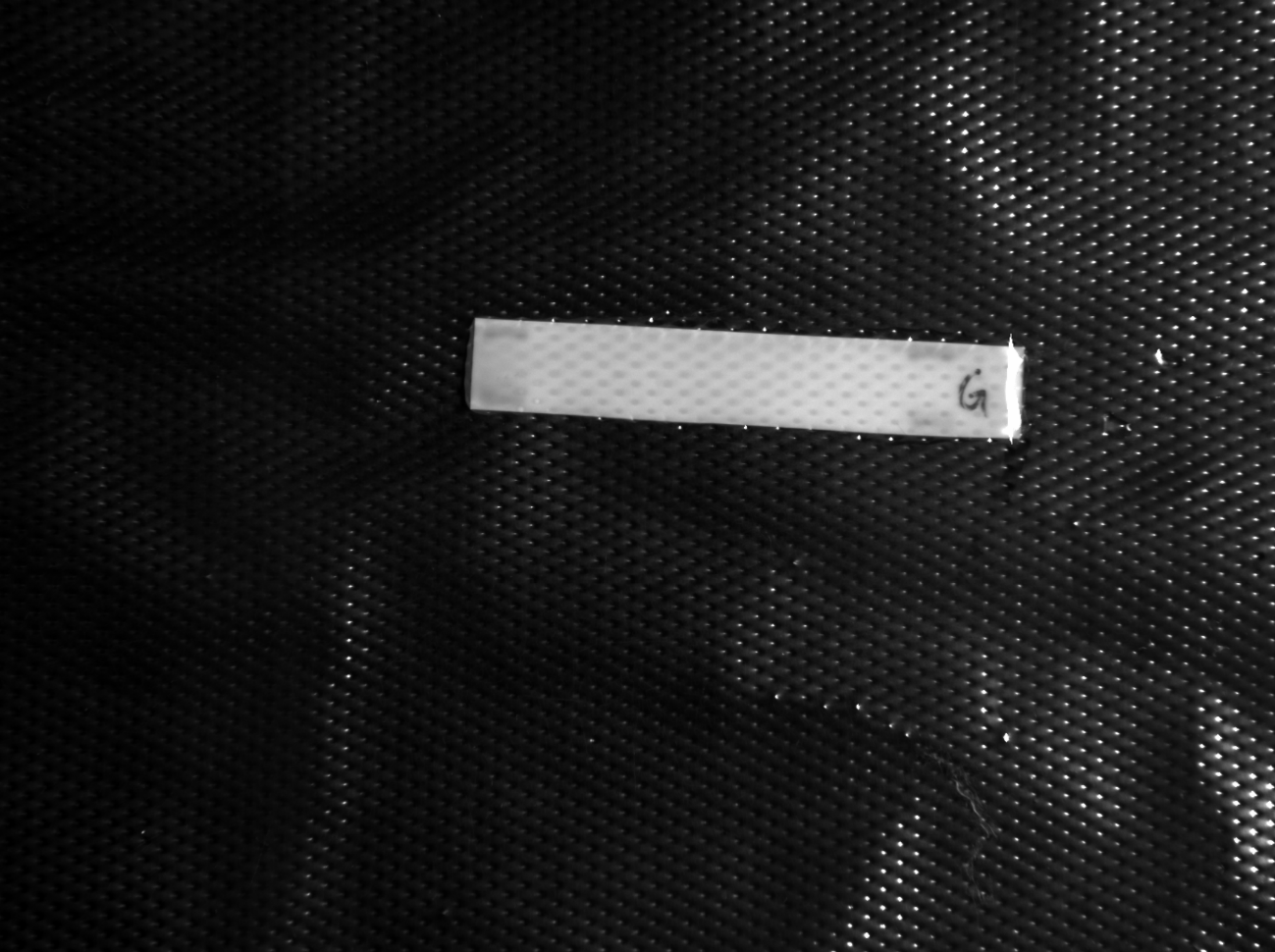


GAPDH

(Colorimetric)


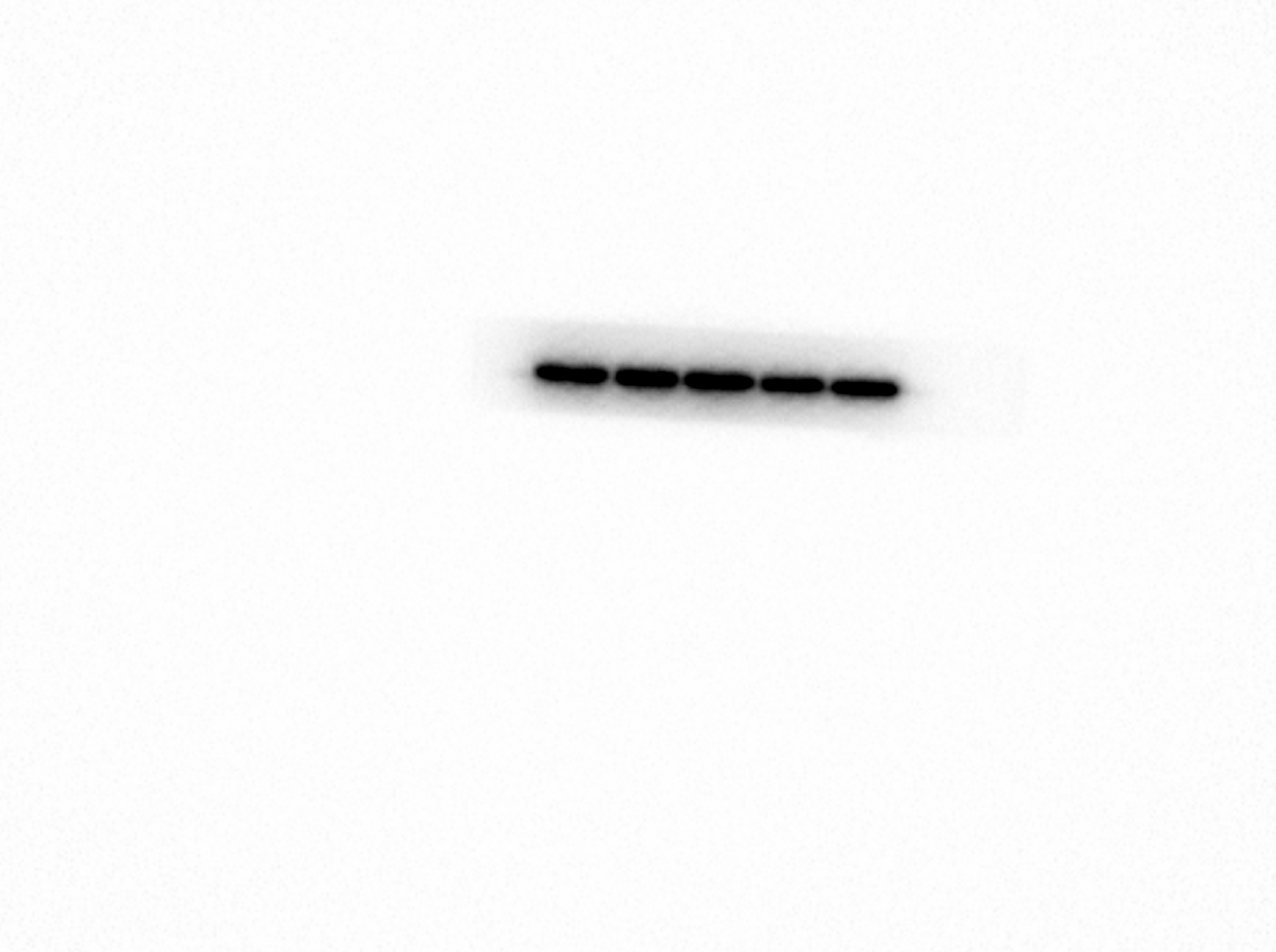


GAPDH

(Chemi)


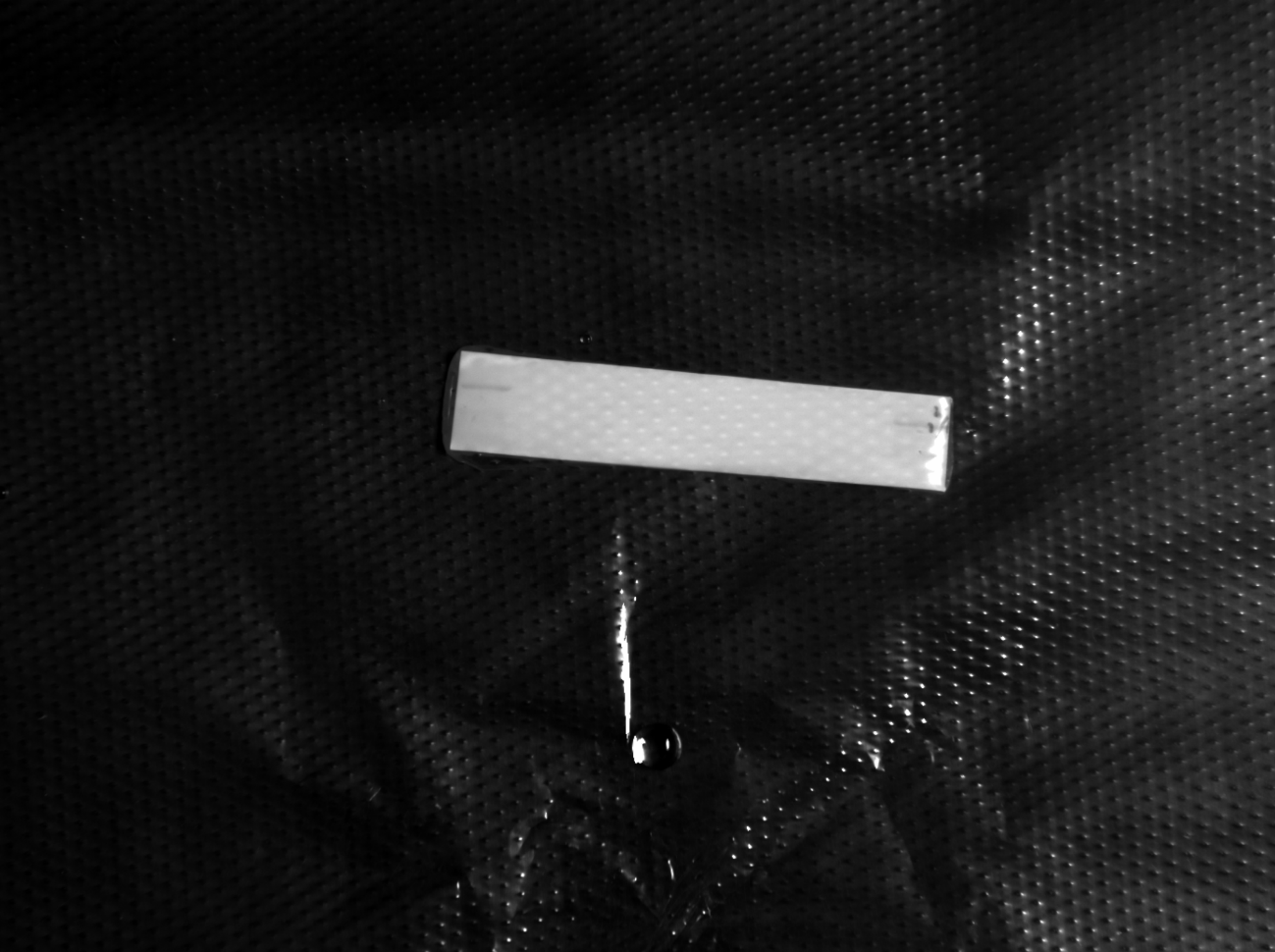


iNOS

(Colorimetric)


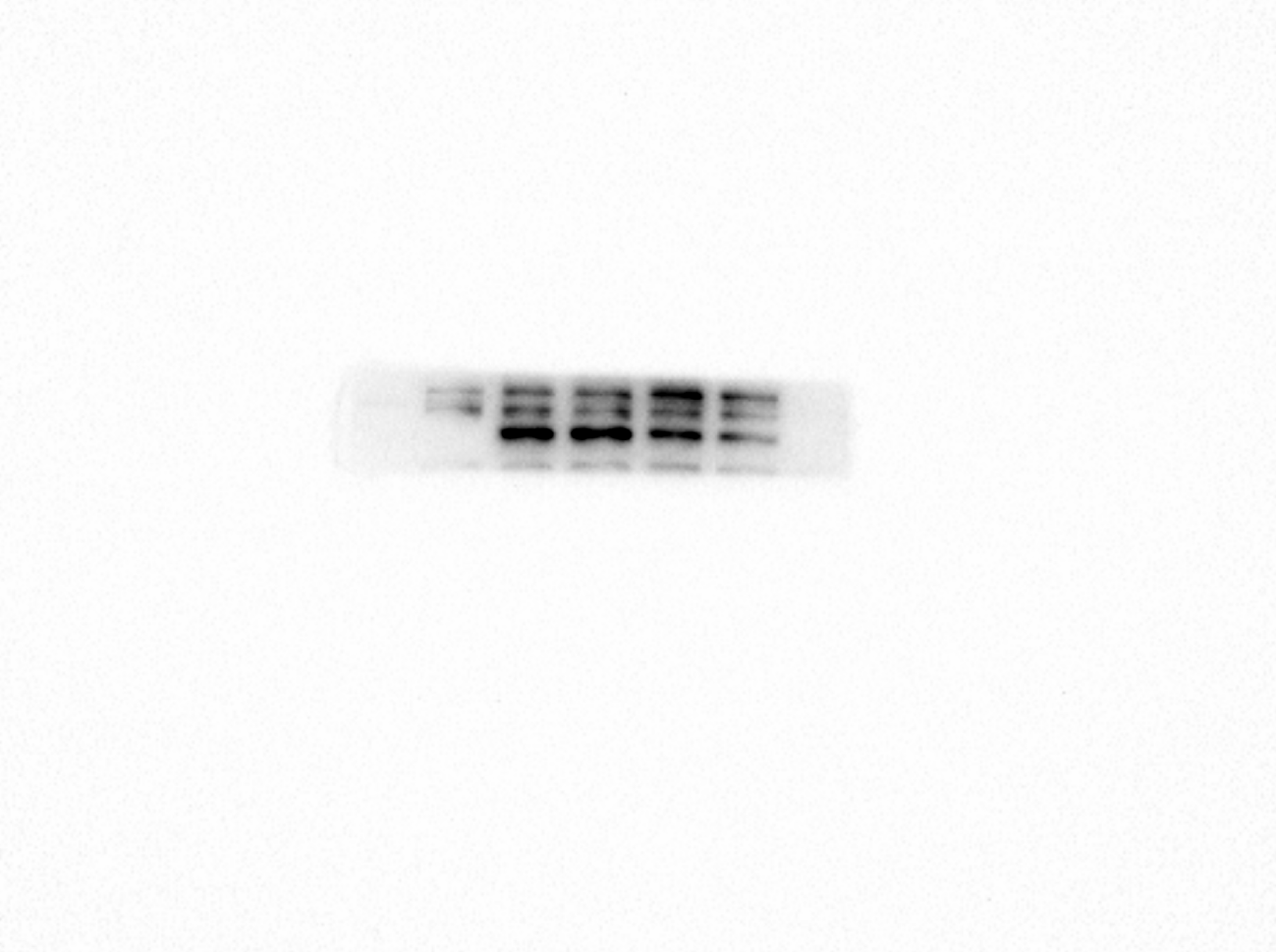


iNOS

(Chemi)


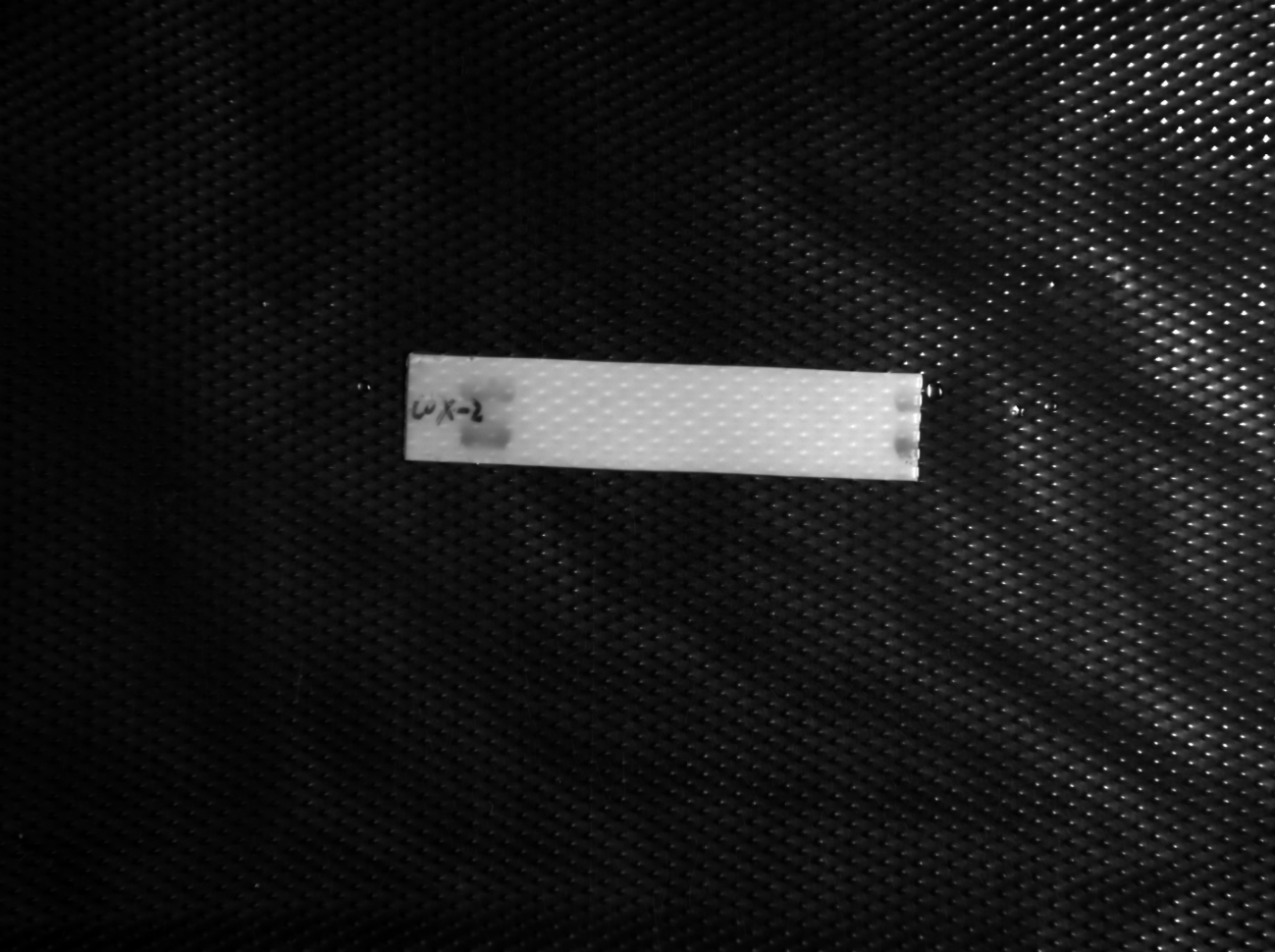


COX-2

(Colorimetric)


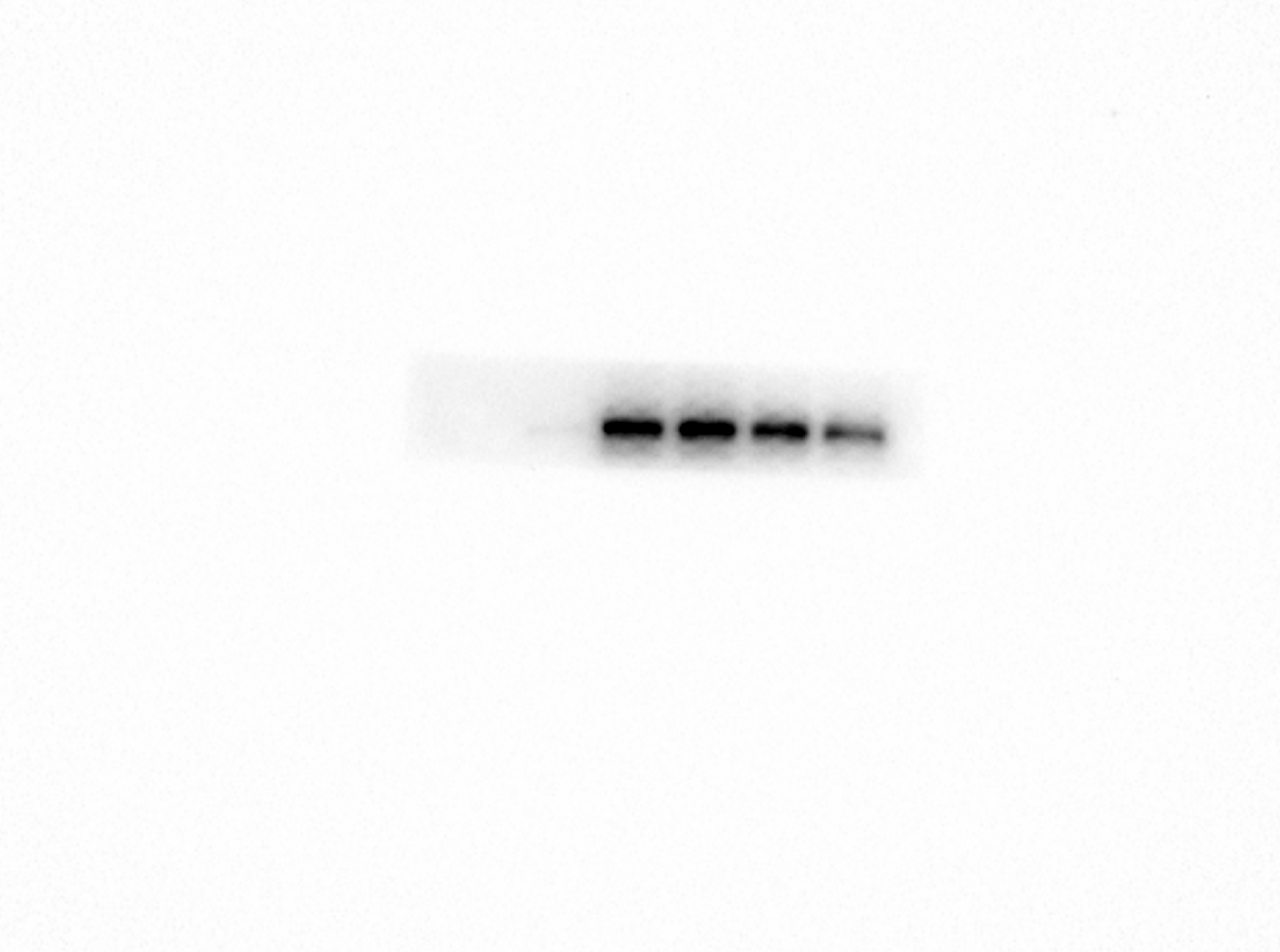


COX-2

(Chemi)


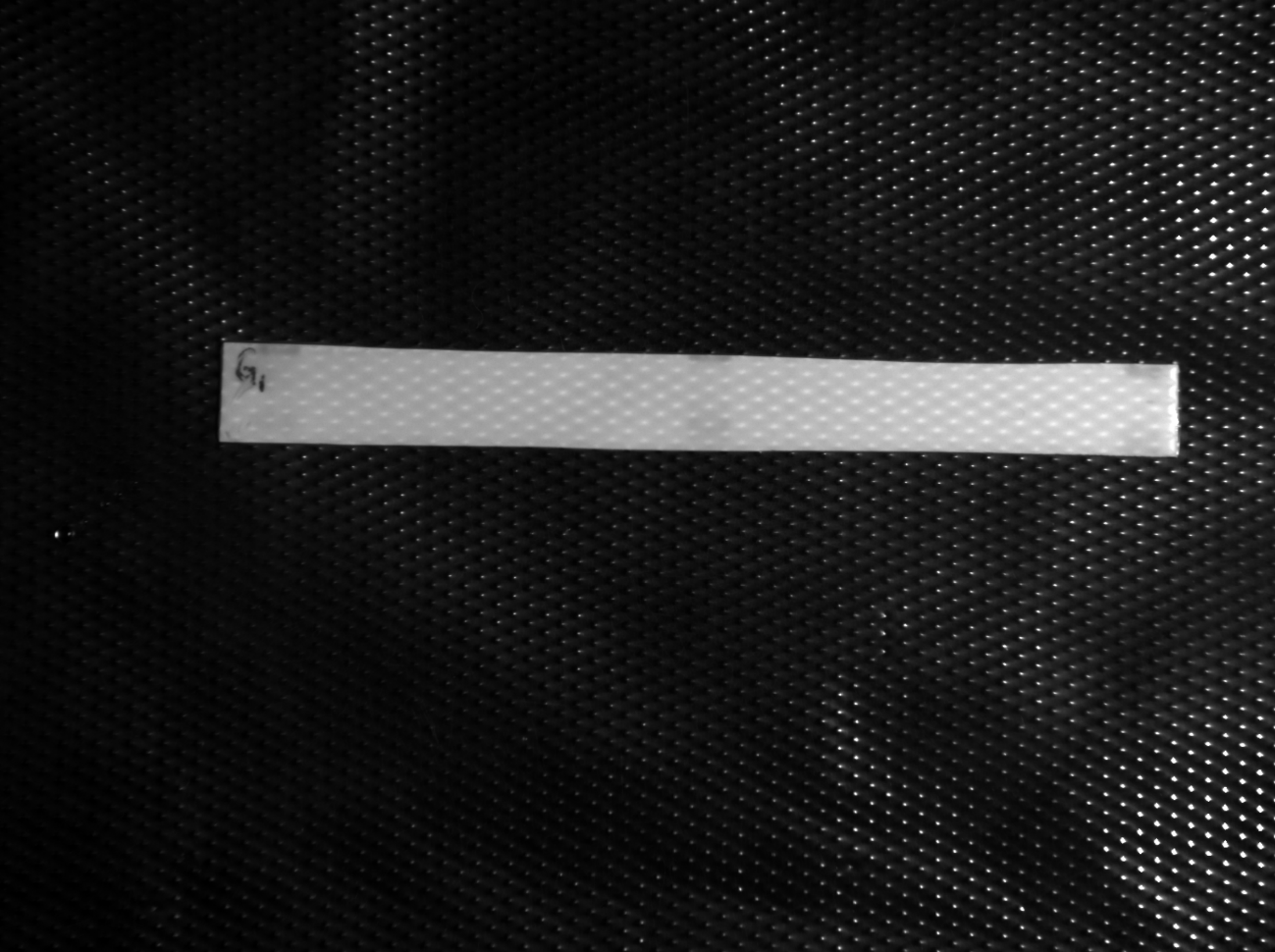
**Figure 3**

GAPDH -left

(Colorimetric)


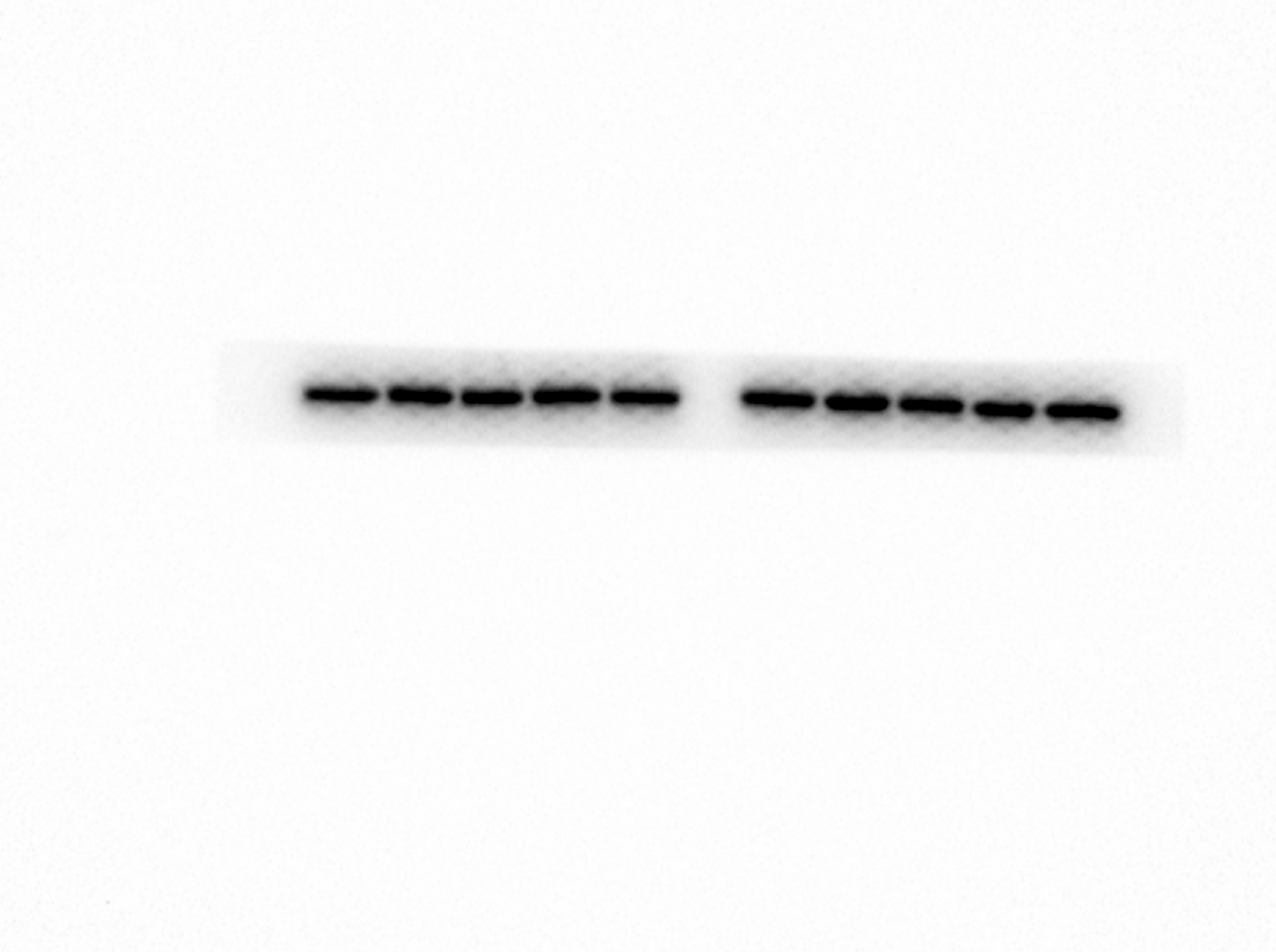


GAPDH -left

(Chemi)


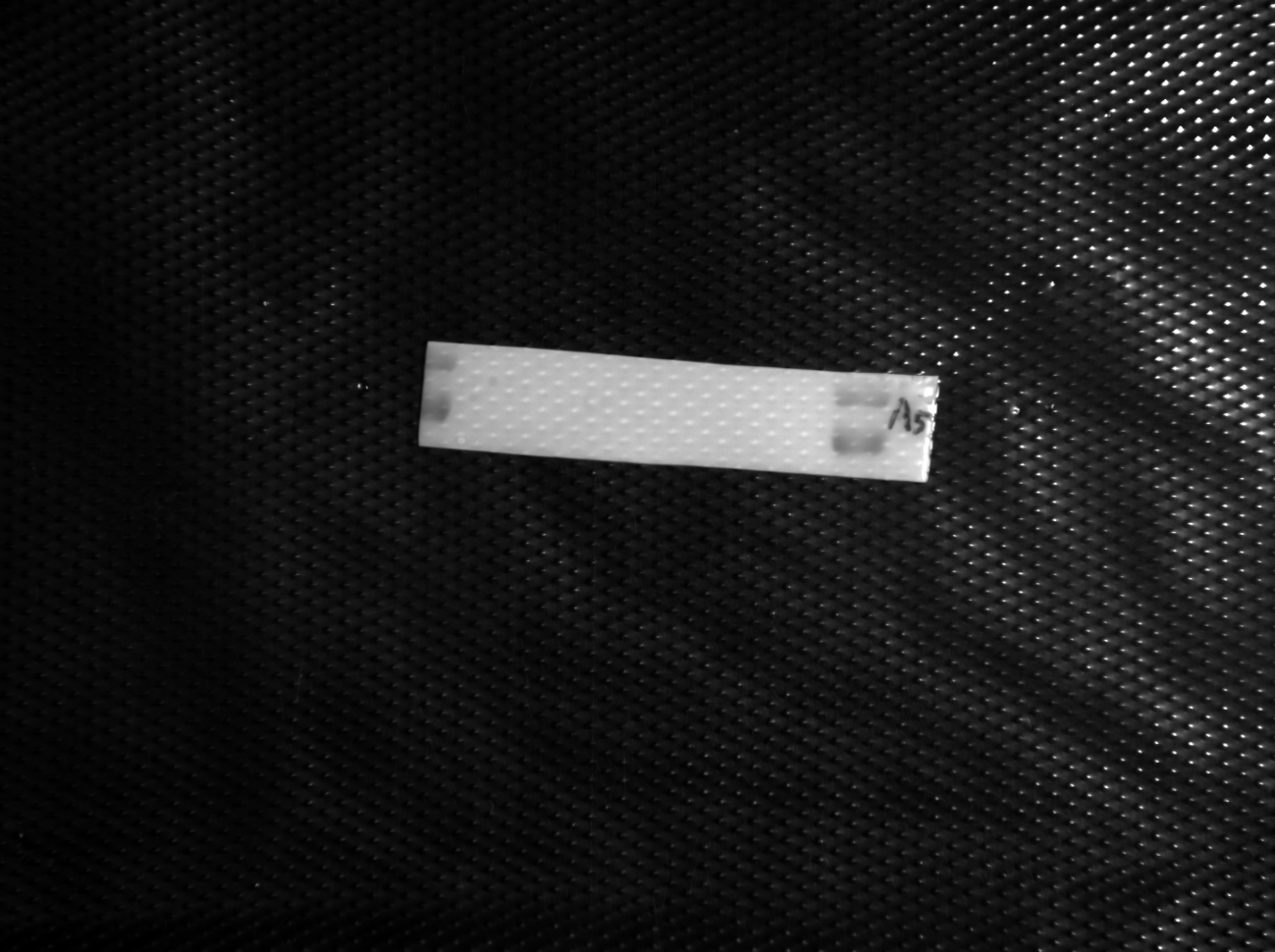


ADAMTS-5

(Colorimetric)


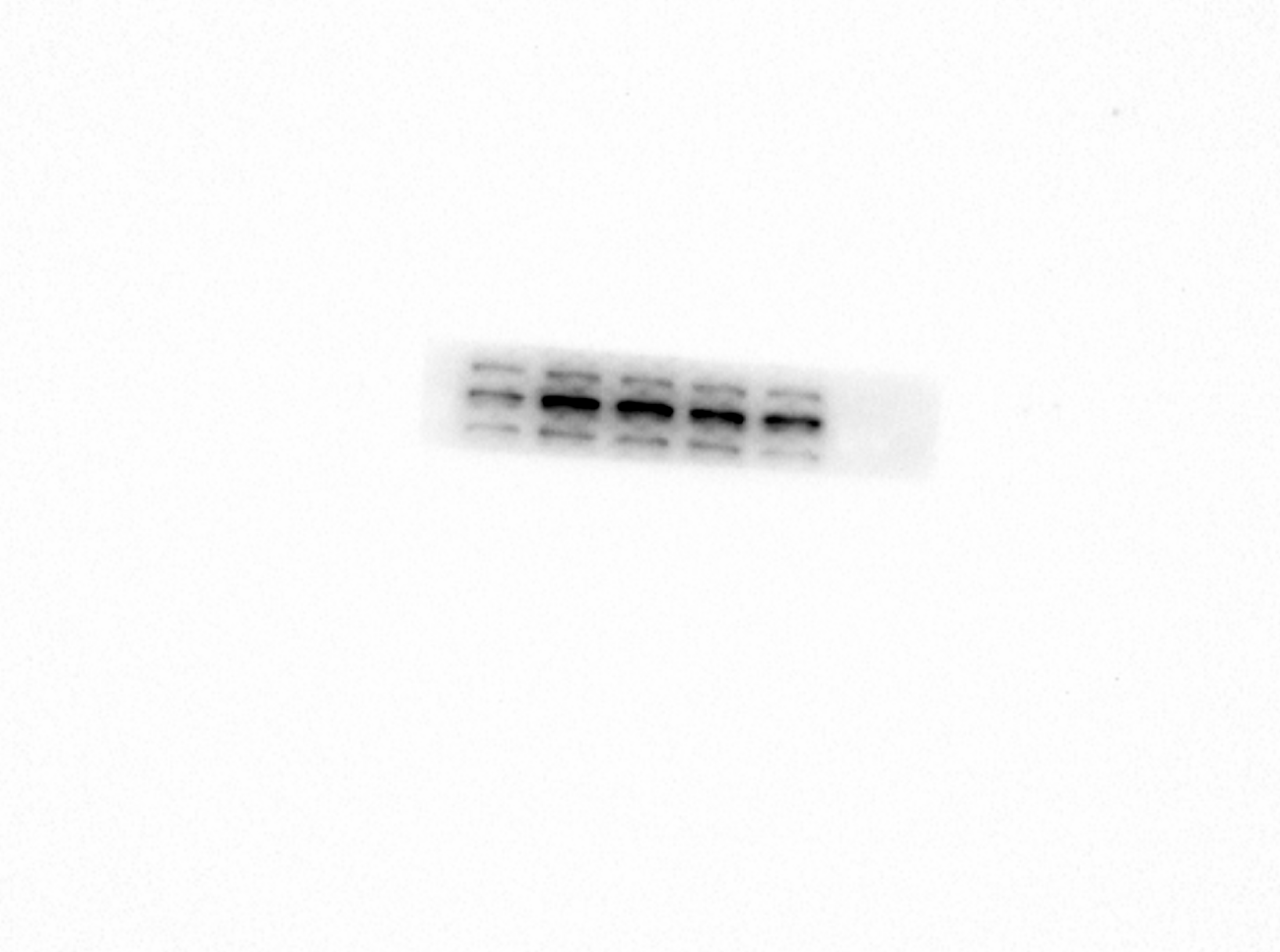


ADAMTS-5

(Chemi)


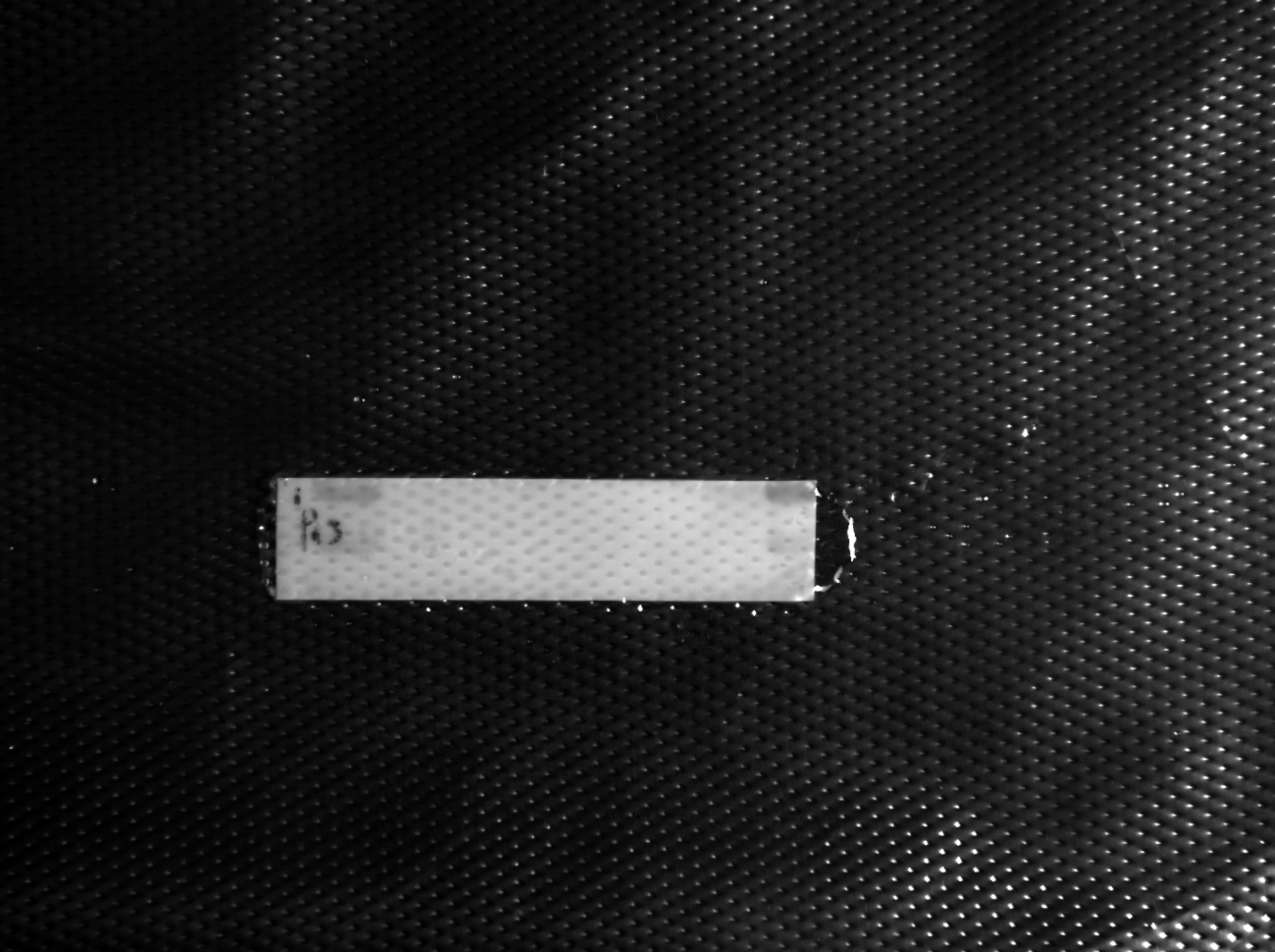


MMP13

(Colorimetric)


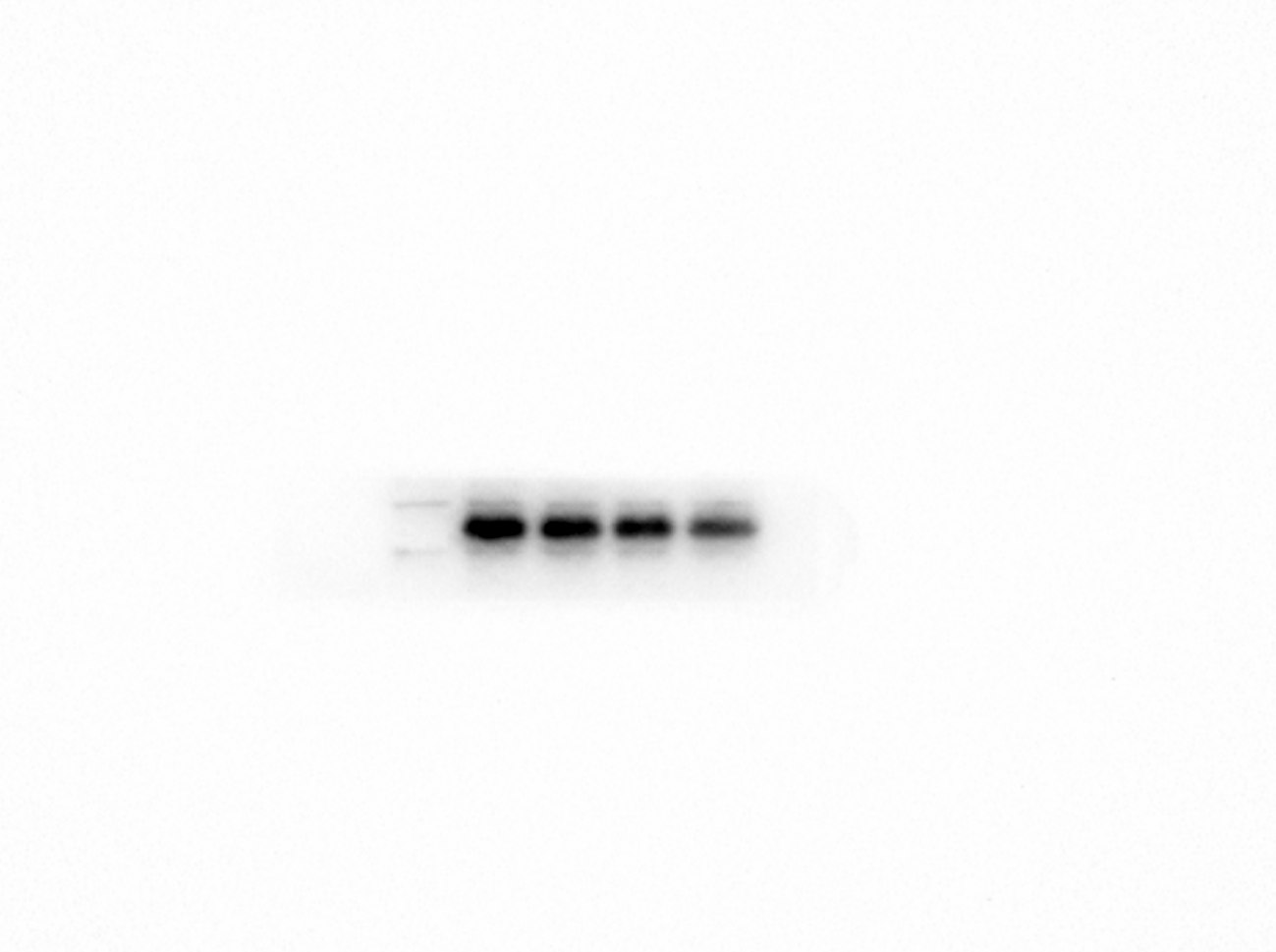


MMP13

(Chemi)


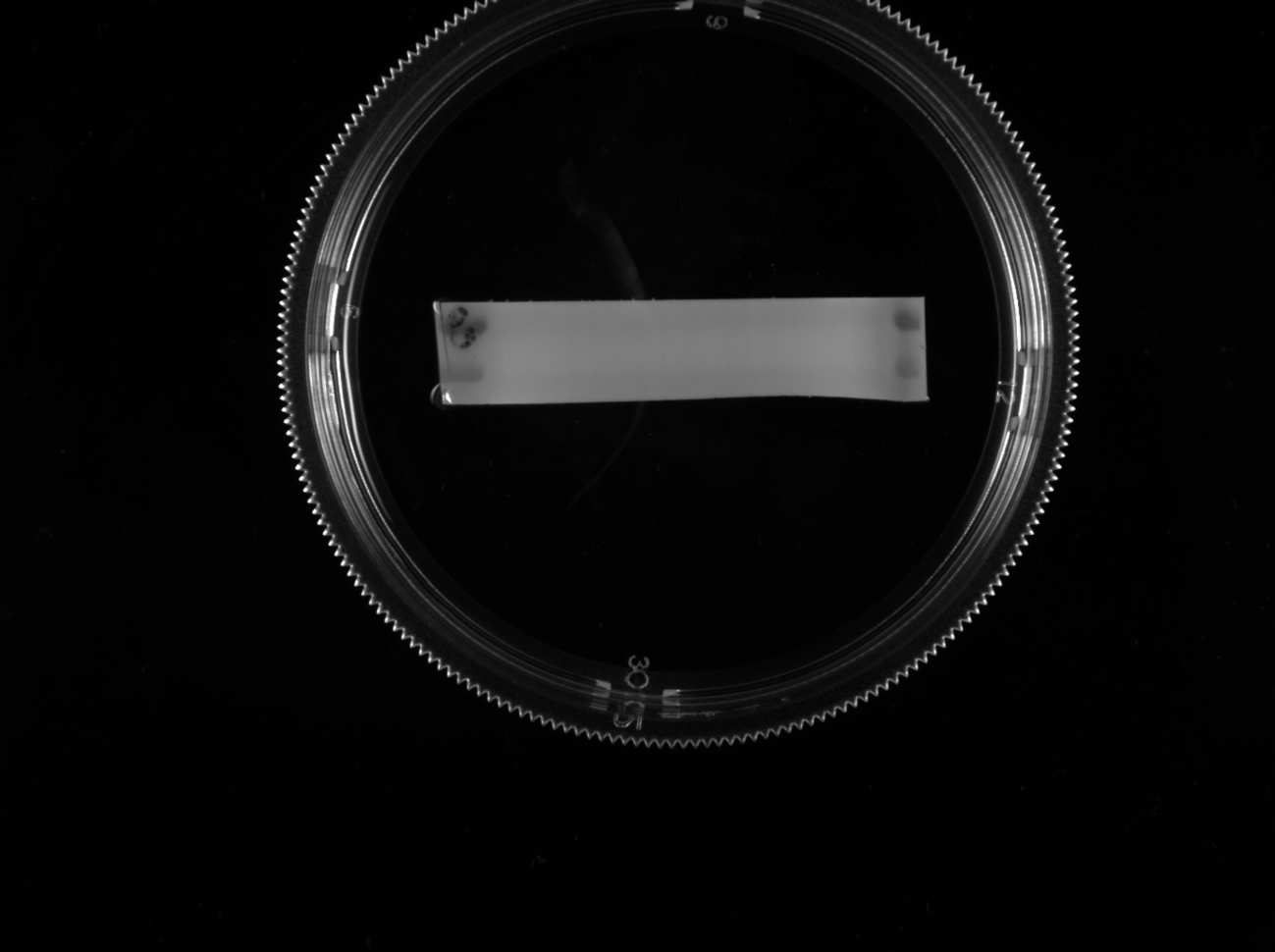


MMP3

(Colorimetric)


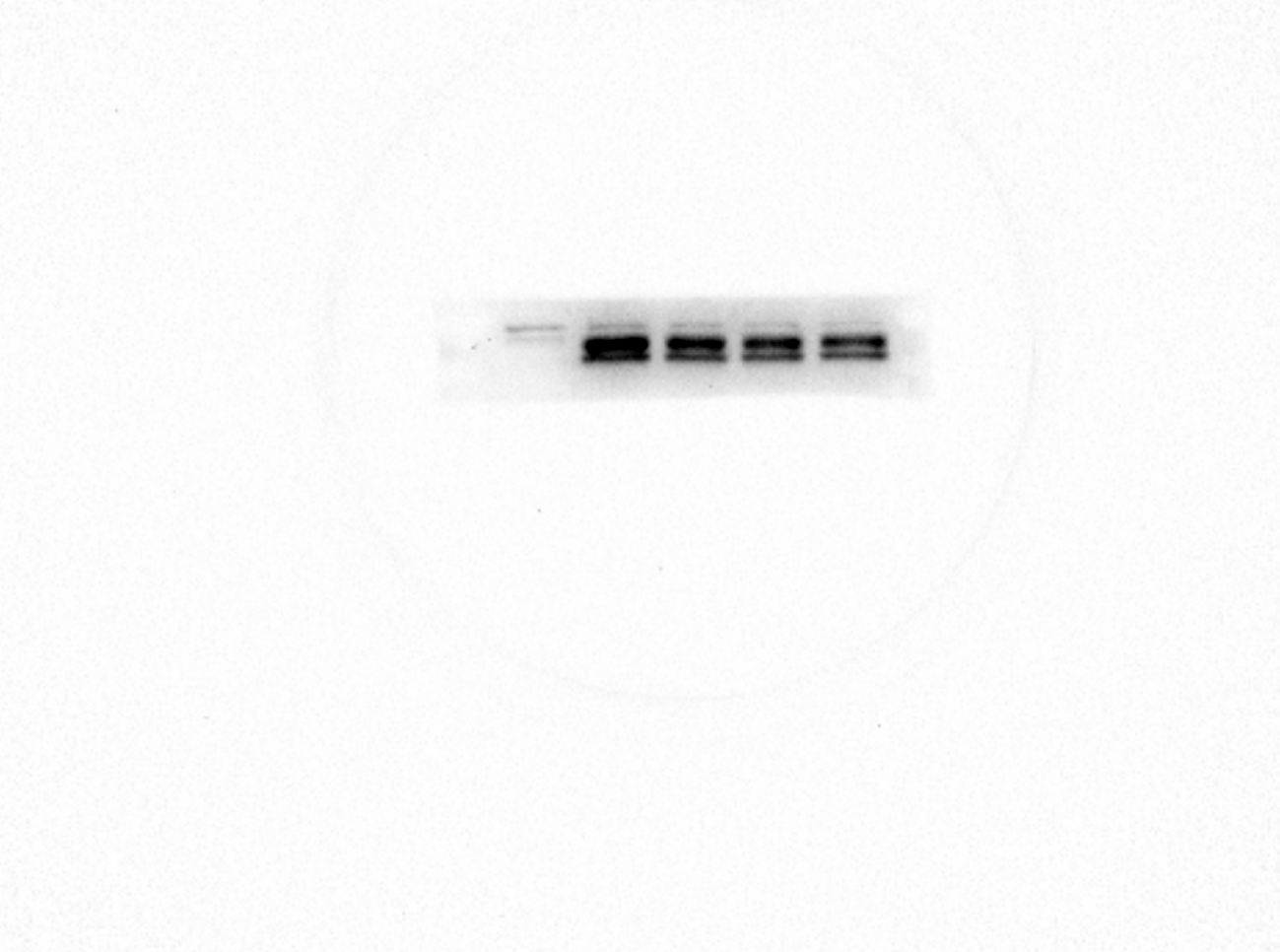


MMP3

(Chemi)


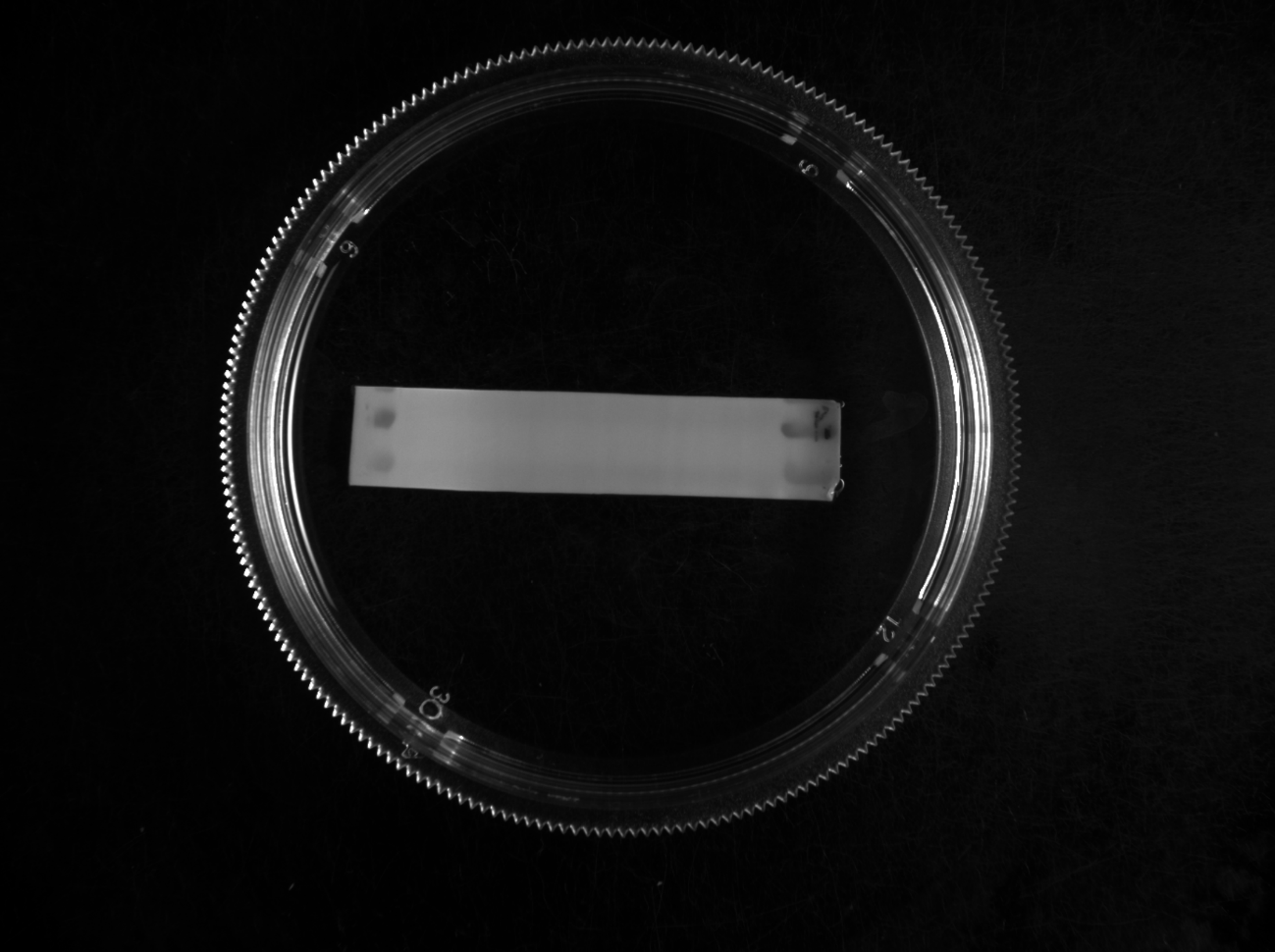


MMP1

(Colorimetric)


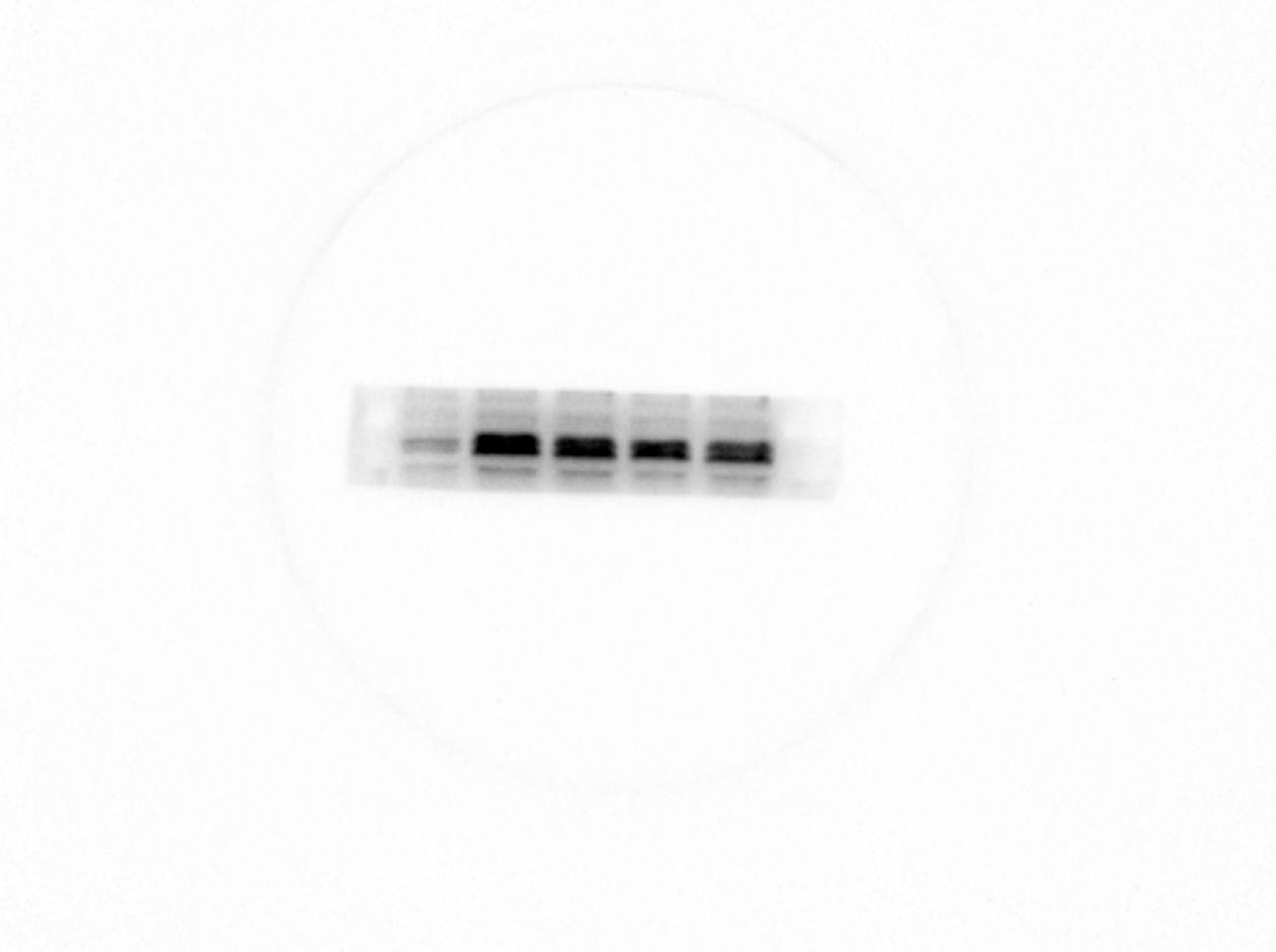


MMP1

(Chemi)

**Figure 4**
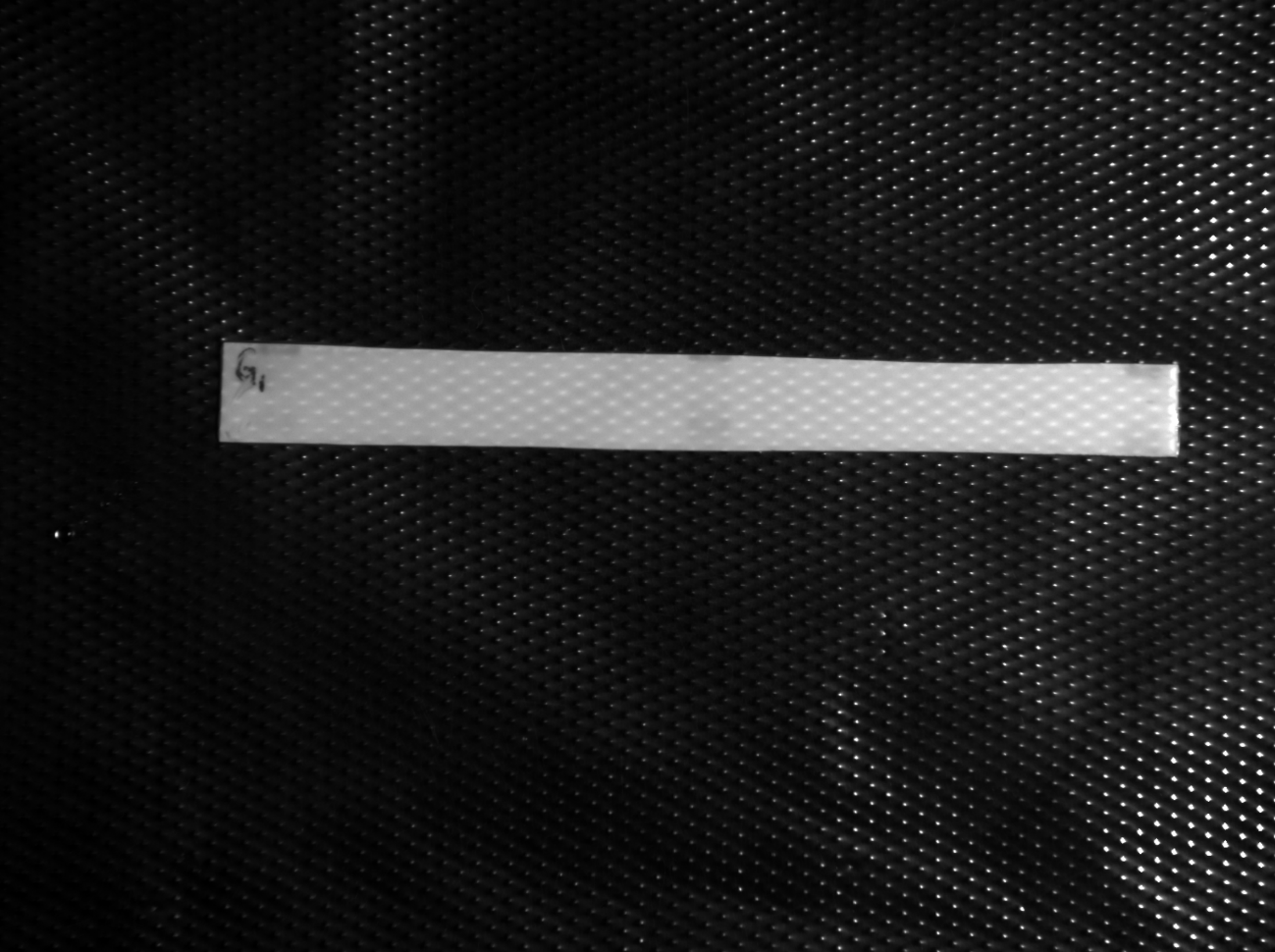


GAPDH -right

(Colorimetric)


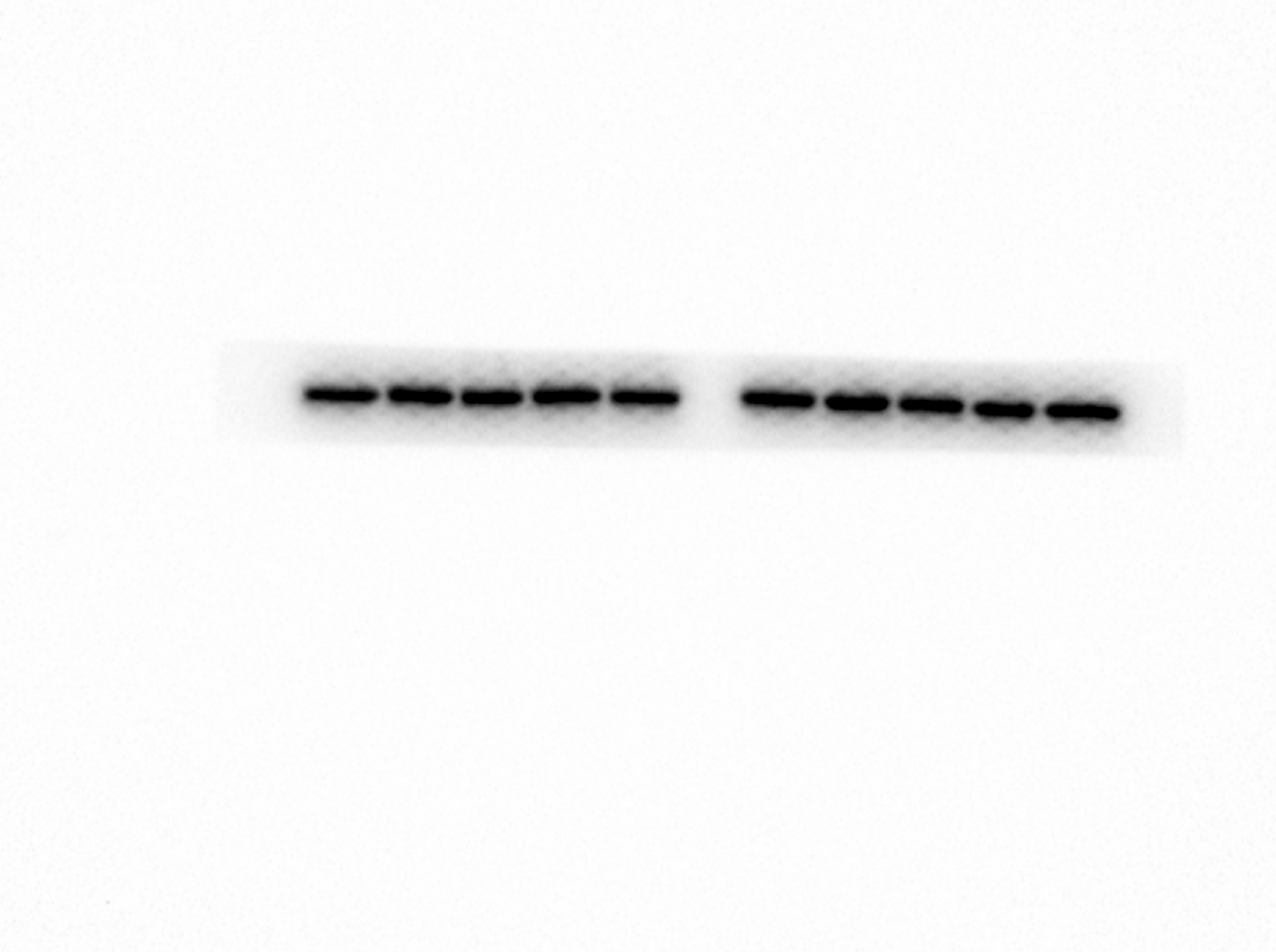


GAPDH - right

(Chemi)


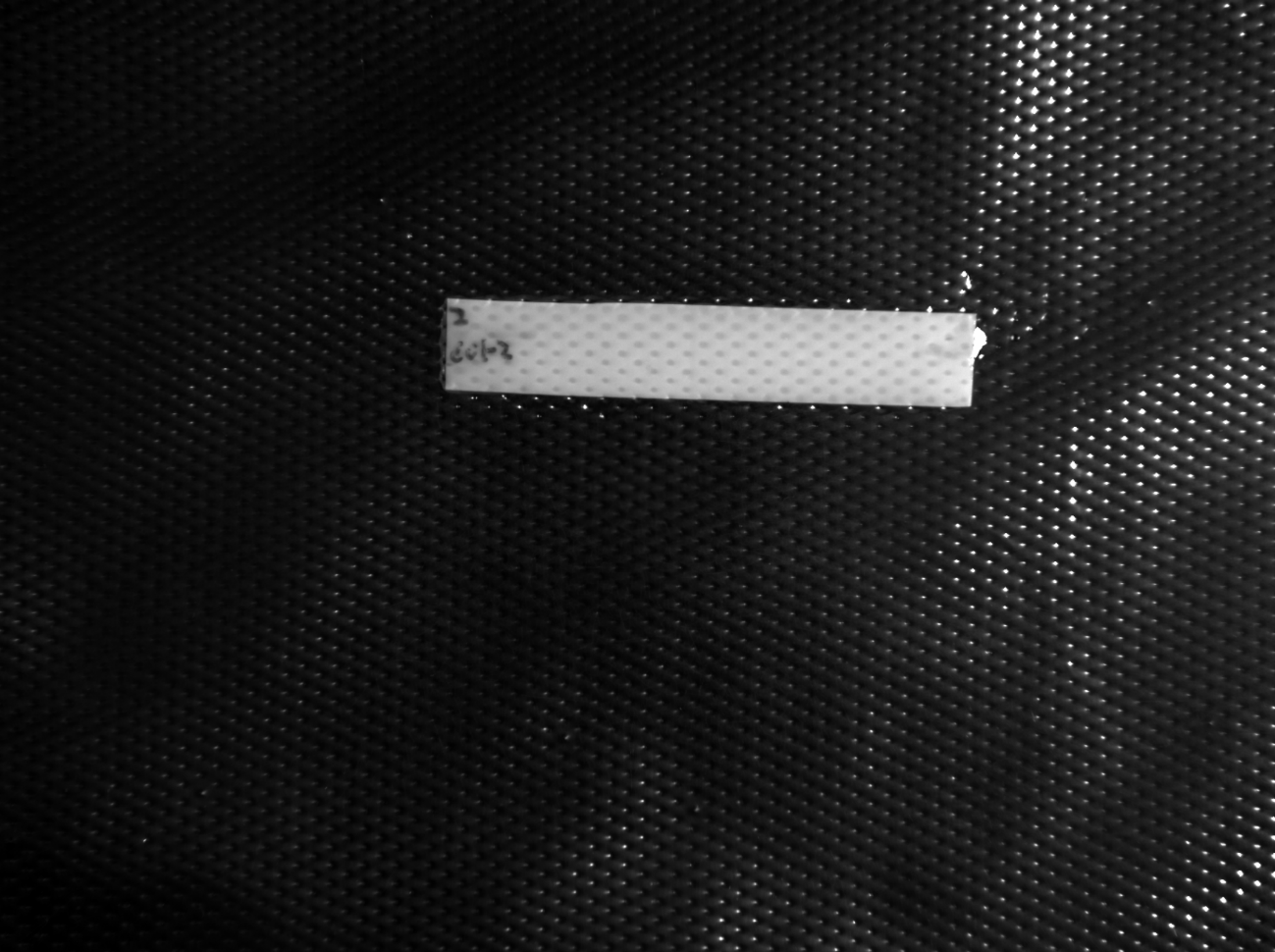


Collagen Ⅱ

(Colorimetric)


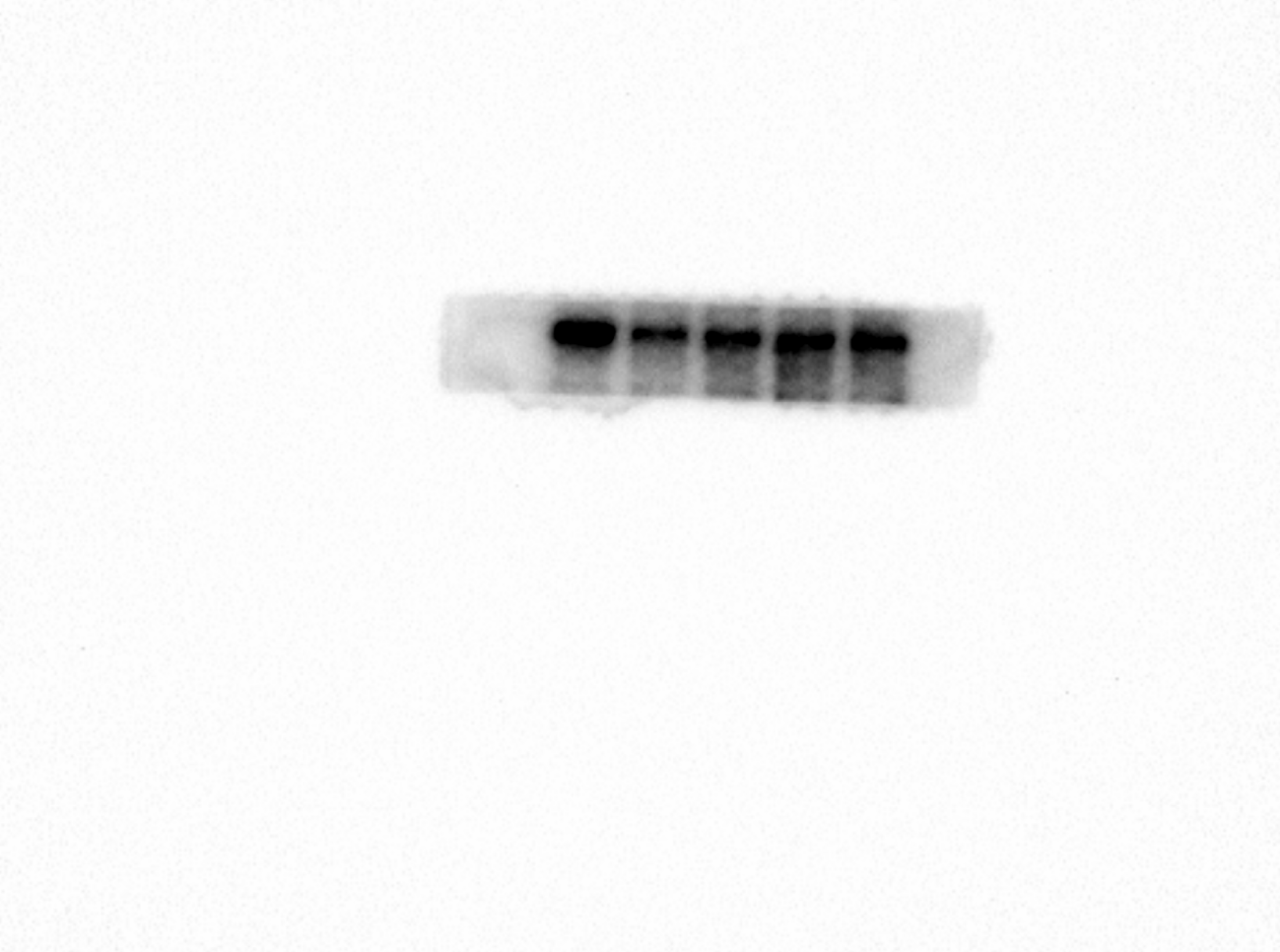


Collagen Ⅱ

(Chemi)


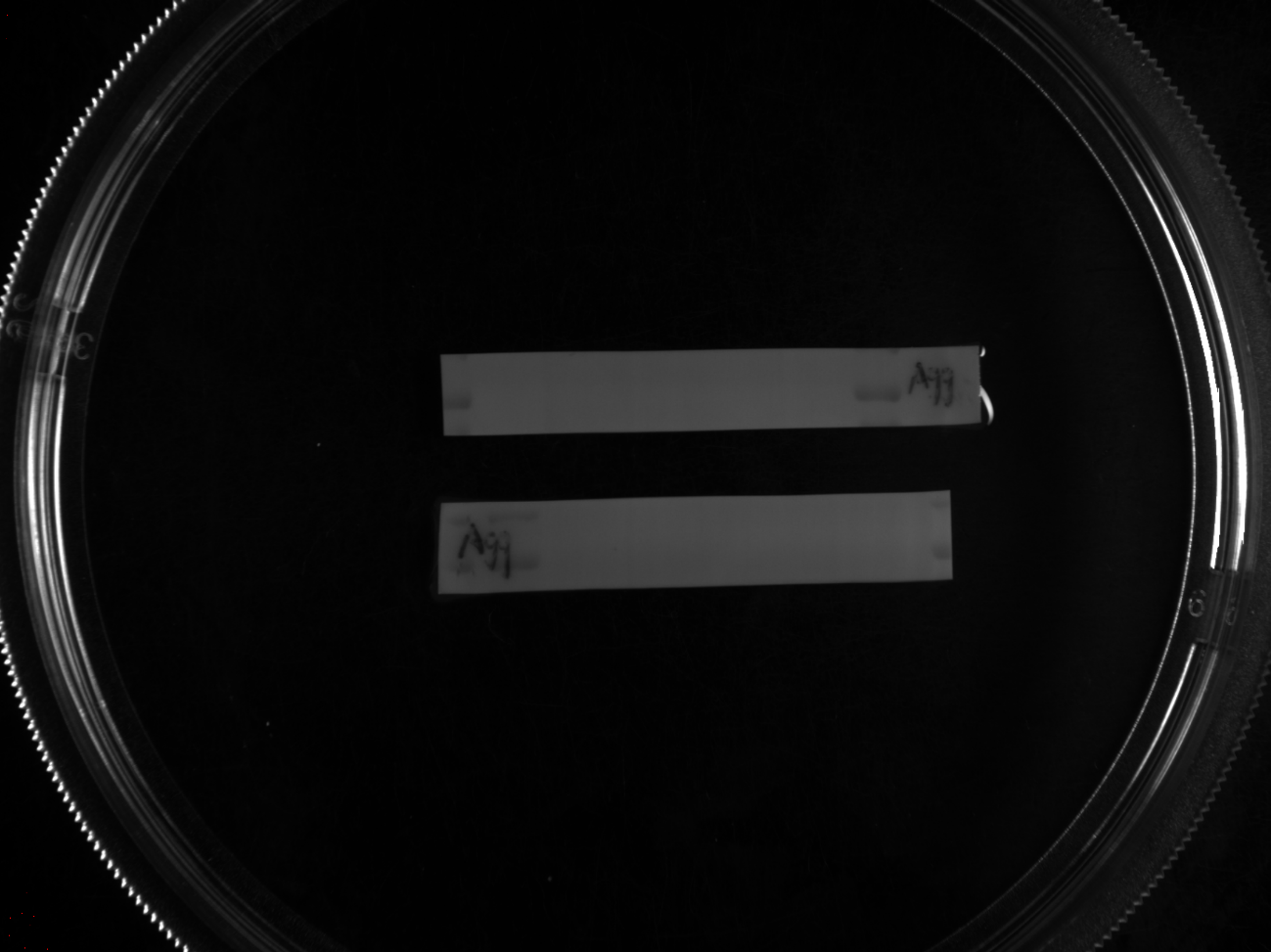


Aggrecan

(Colorimetric)


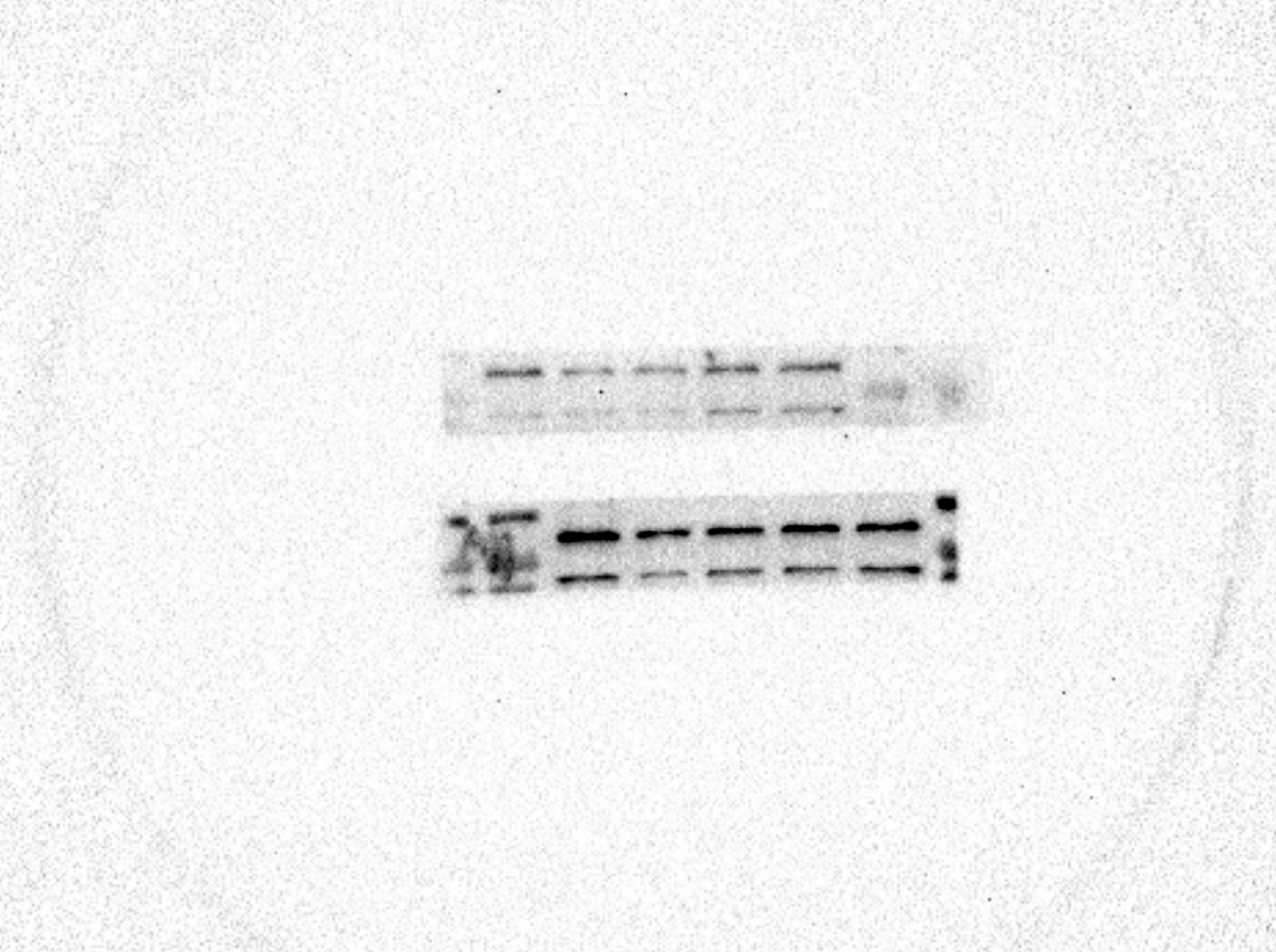


Aggrecan

(Chemi)

**Figure 5**


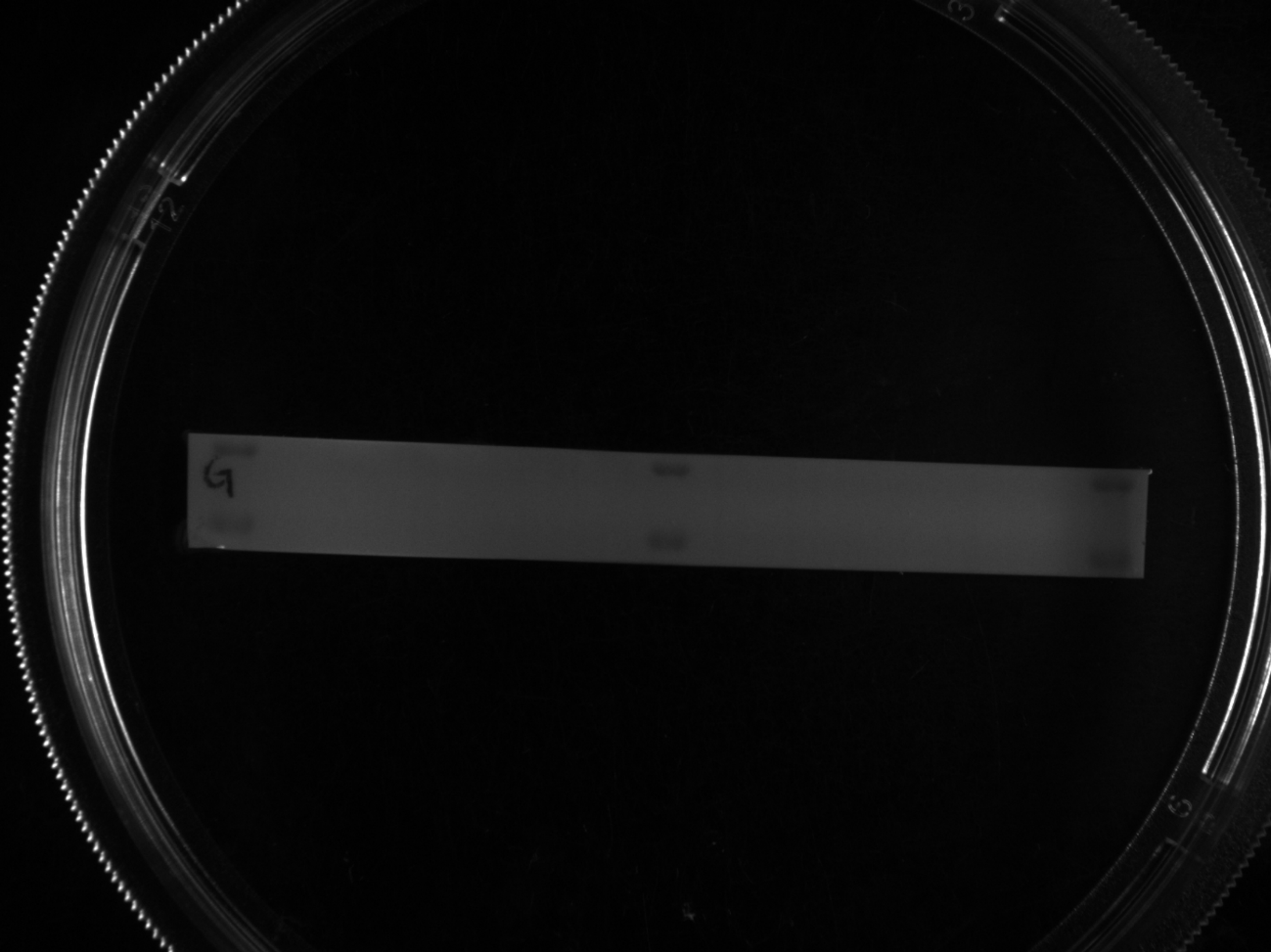


GAPDH-right

(Colorimetric)


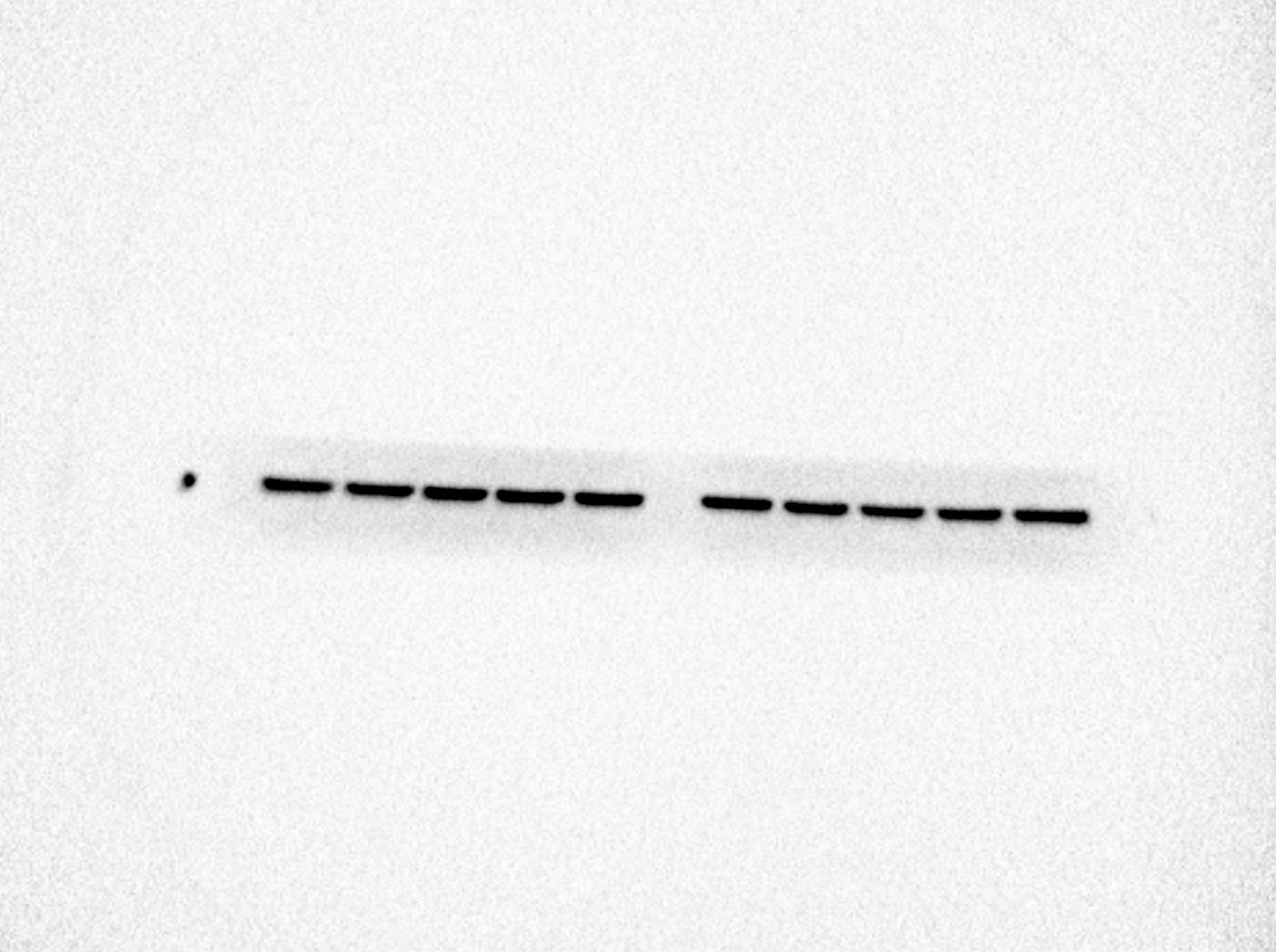


GAPDH-right

(Chemi)


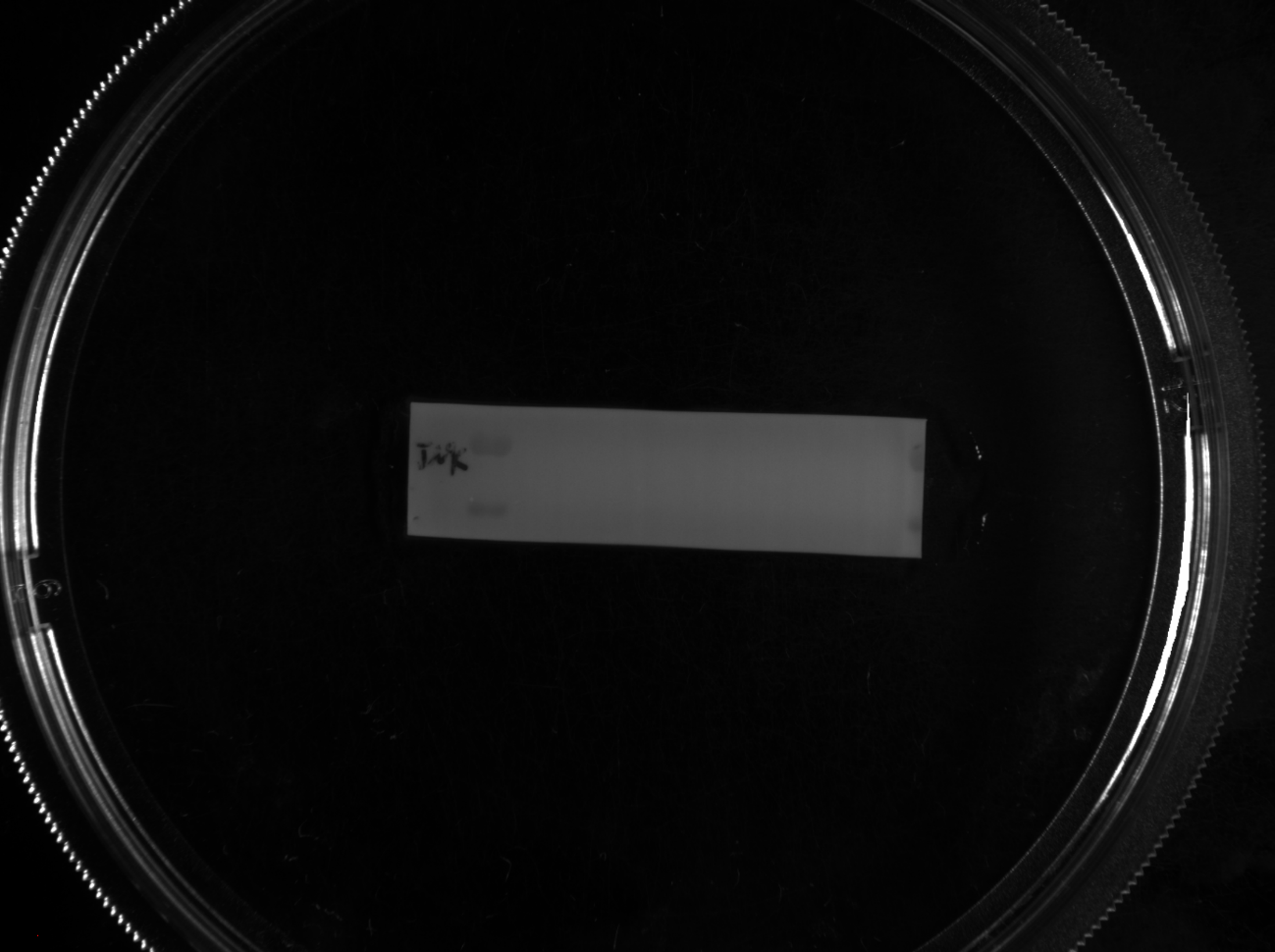


JNK

(Colorimetric)


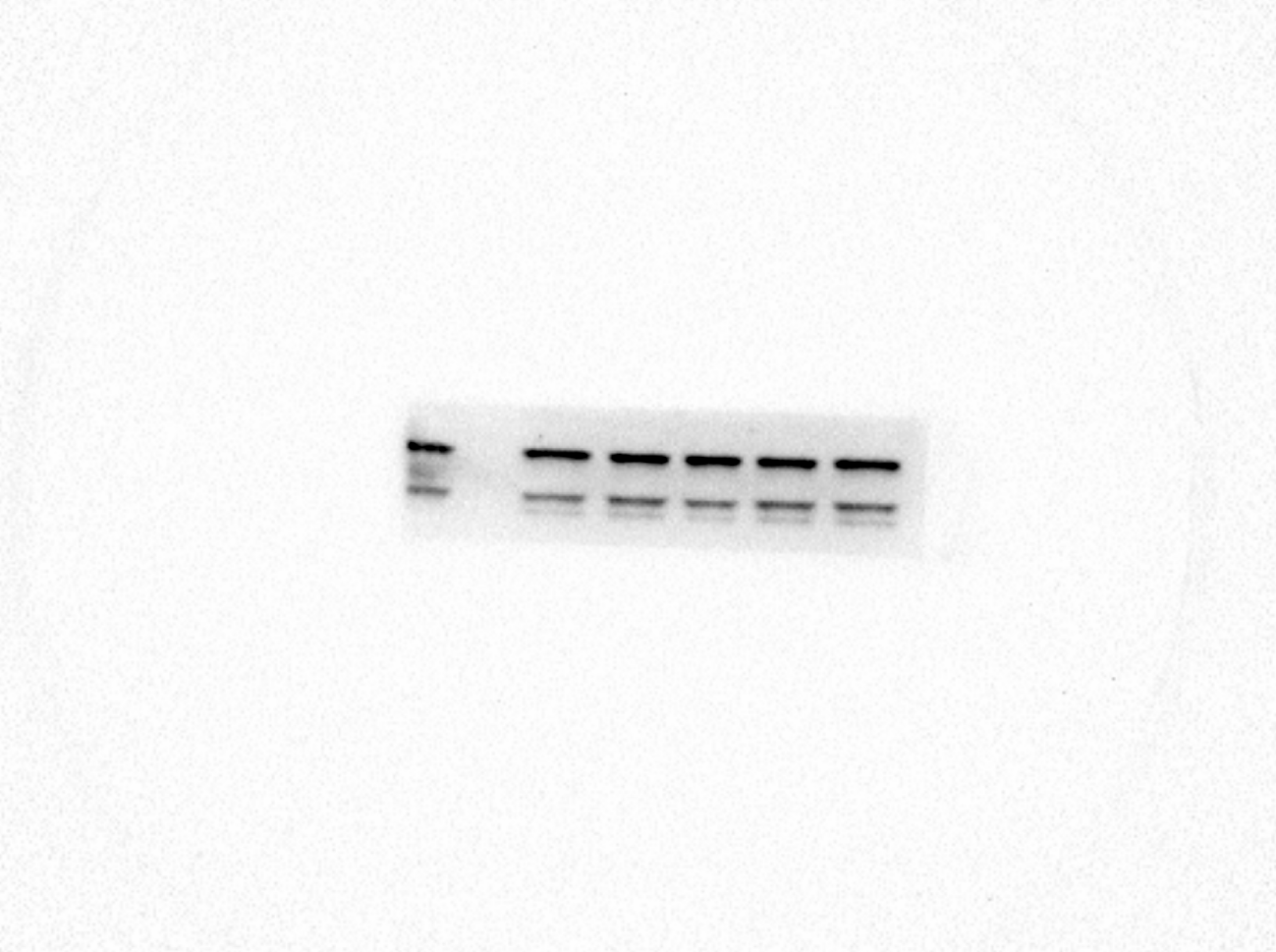


JNK

(Chemi)


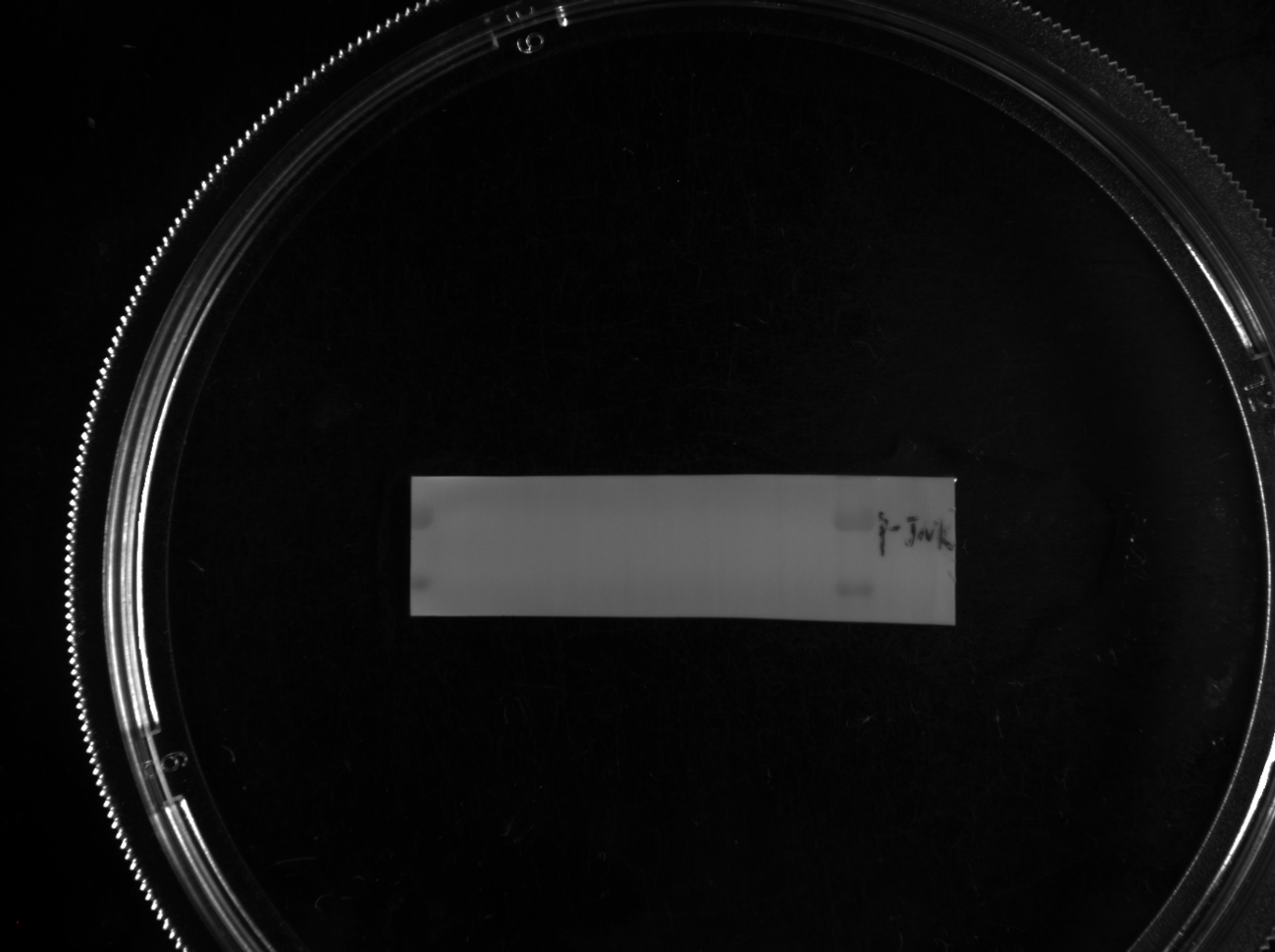


P-JNK

(Colorimetric)


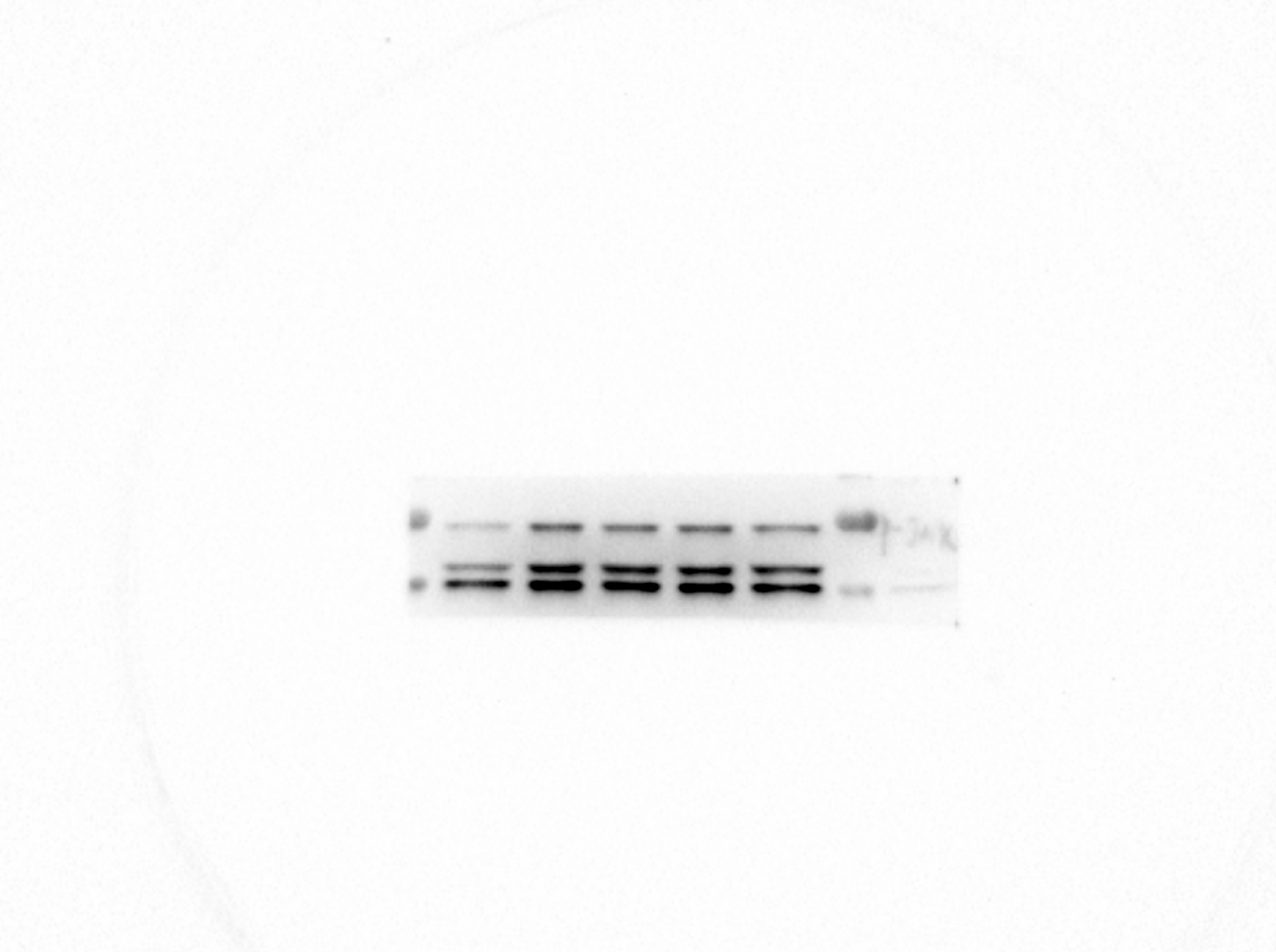


P-JNK

(Chemi)


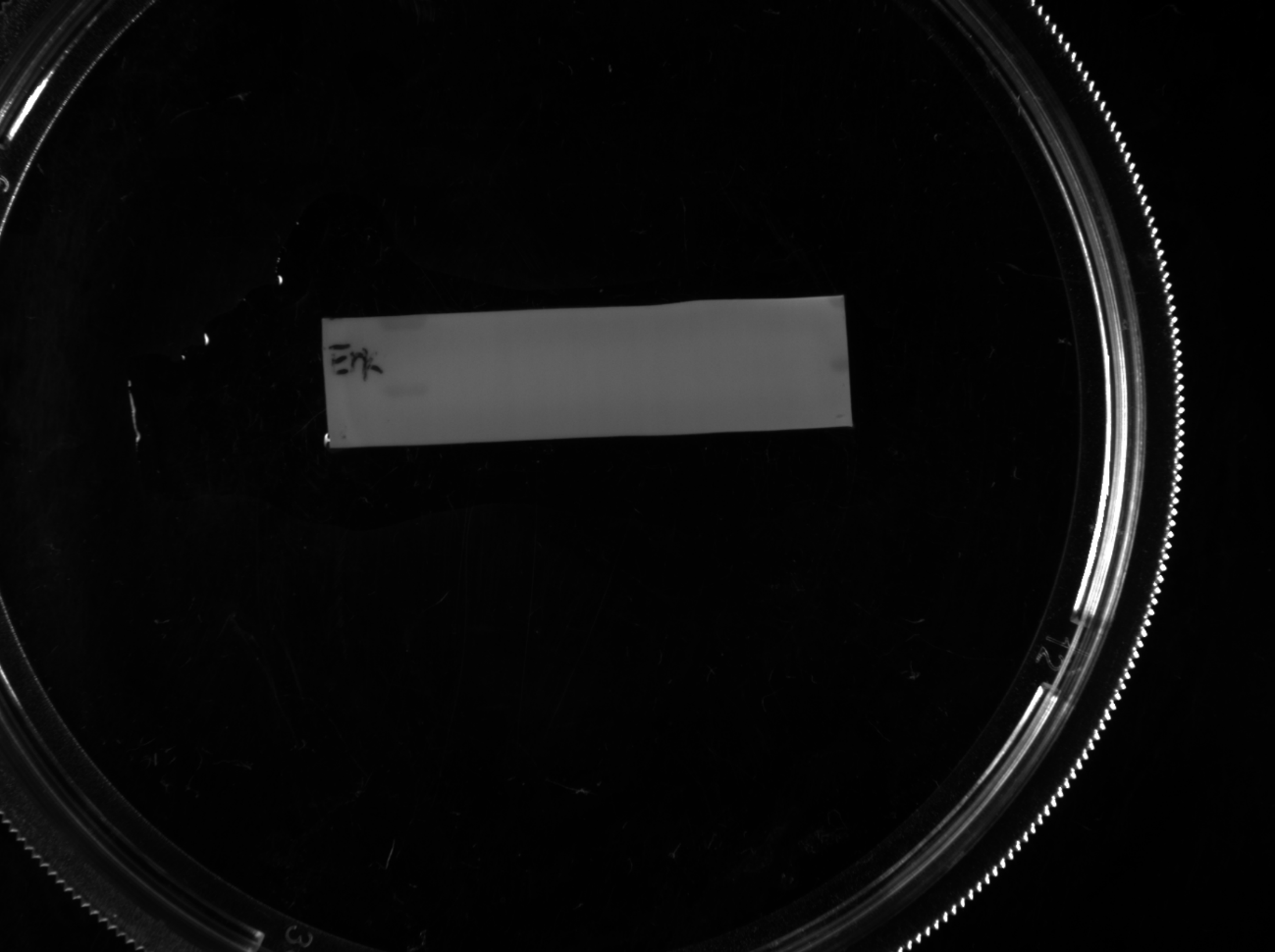


ERK

(Colorimetric)


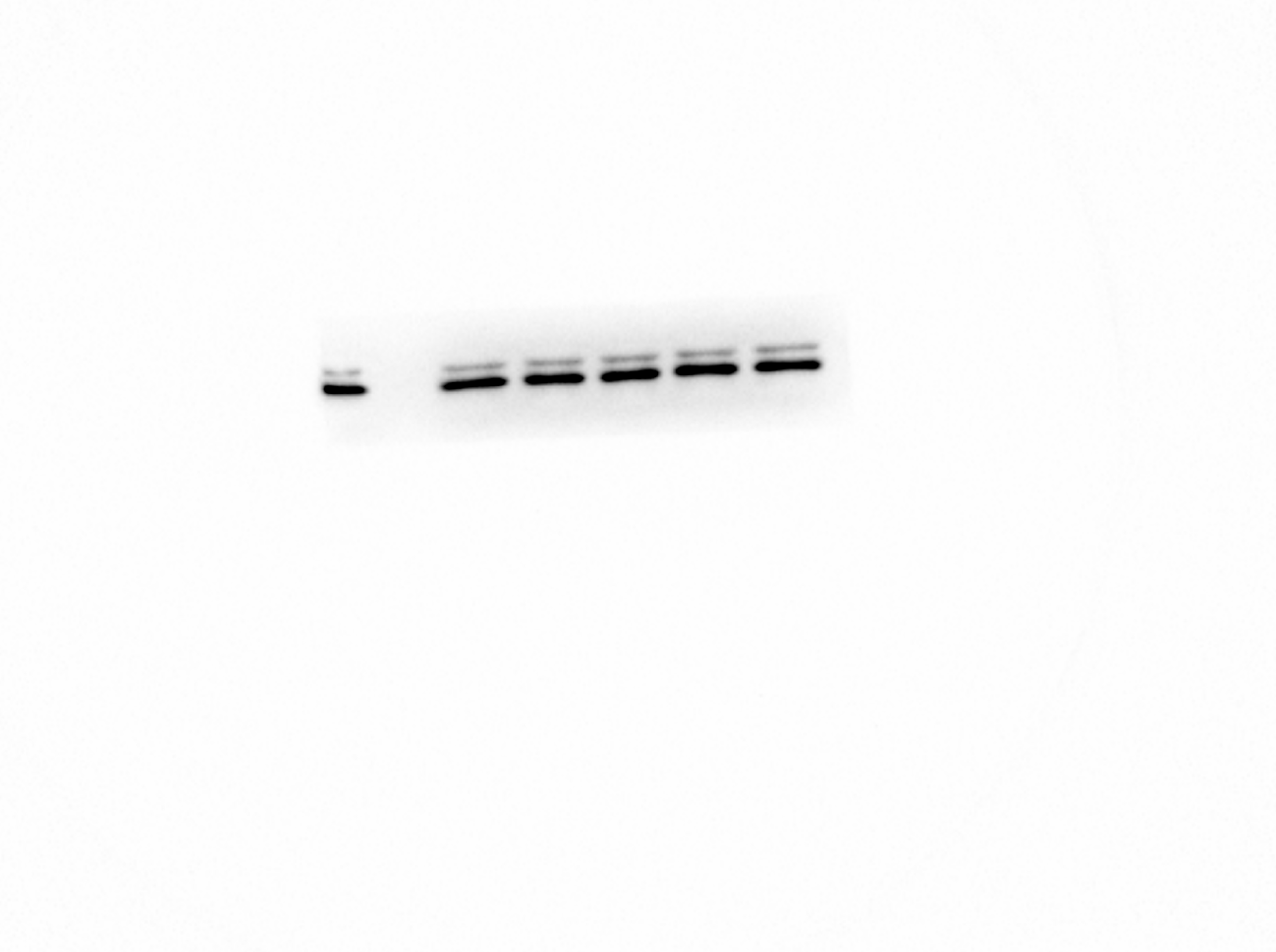


ERK

(Chemi)


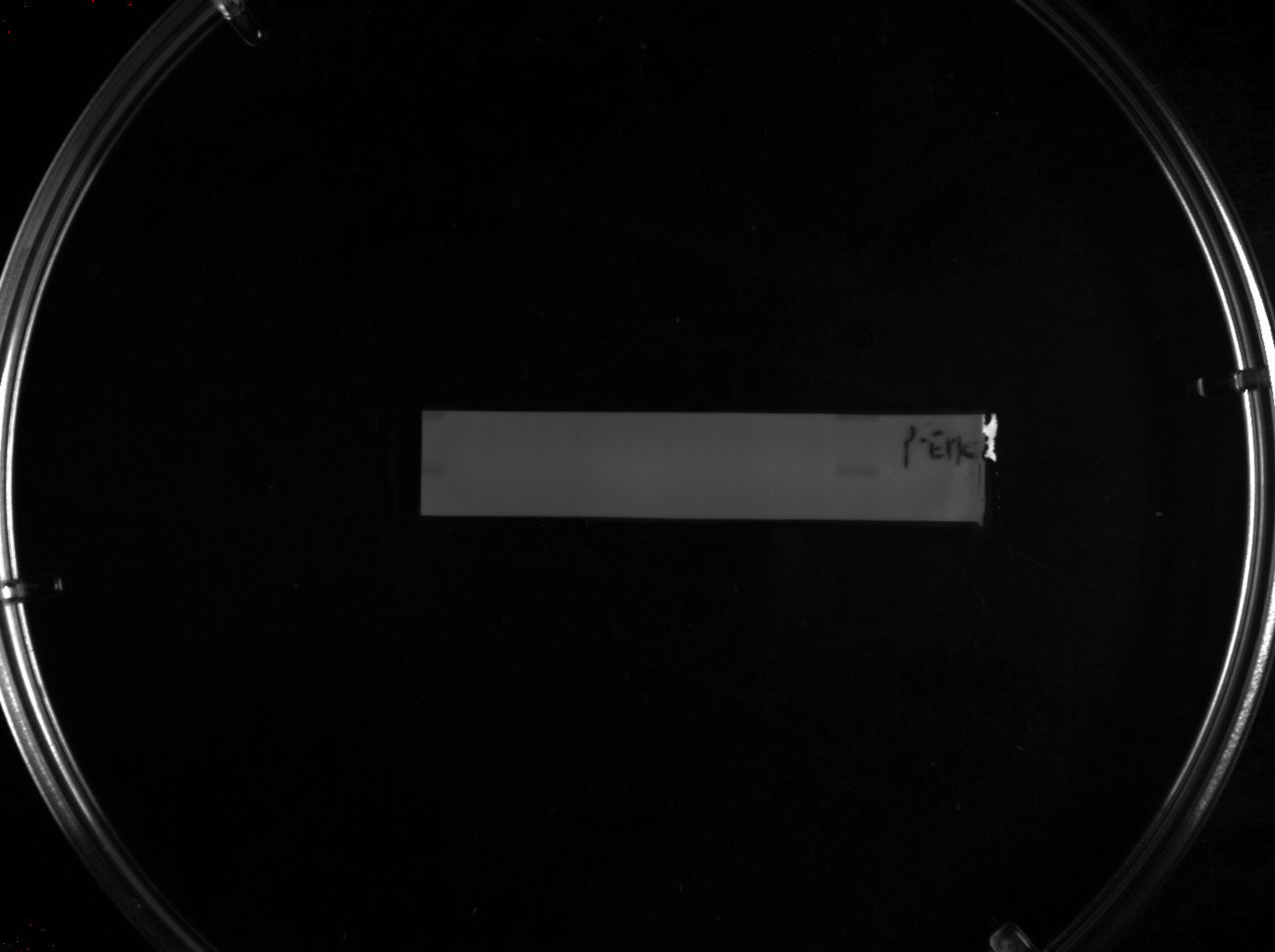


P-ERK

(Colorimetric)


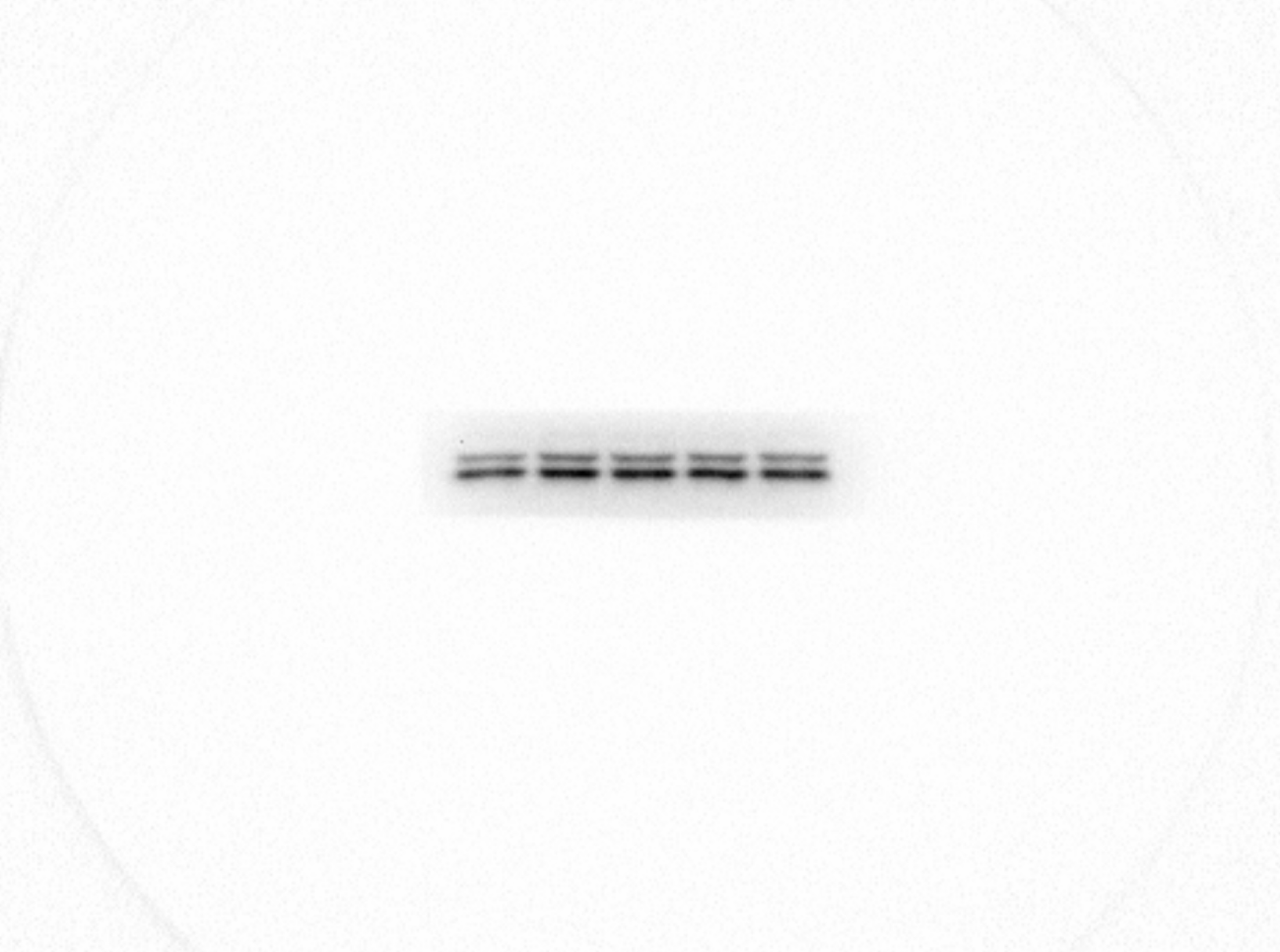


P-ERK

(Chemi)


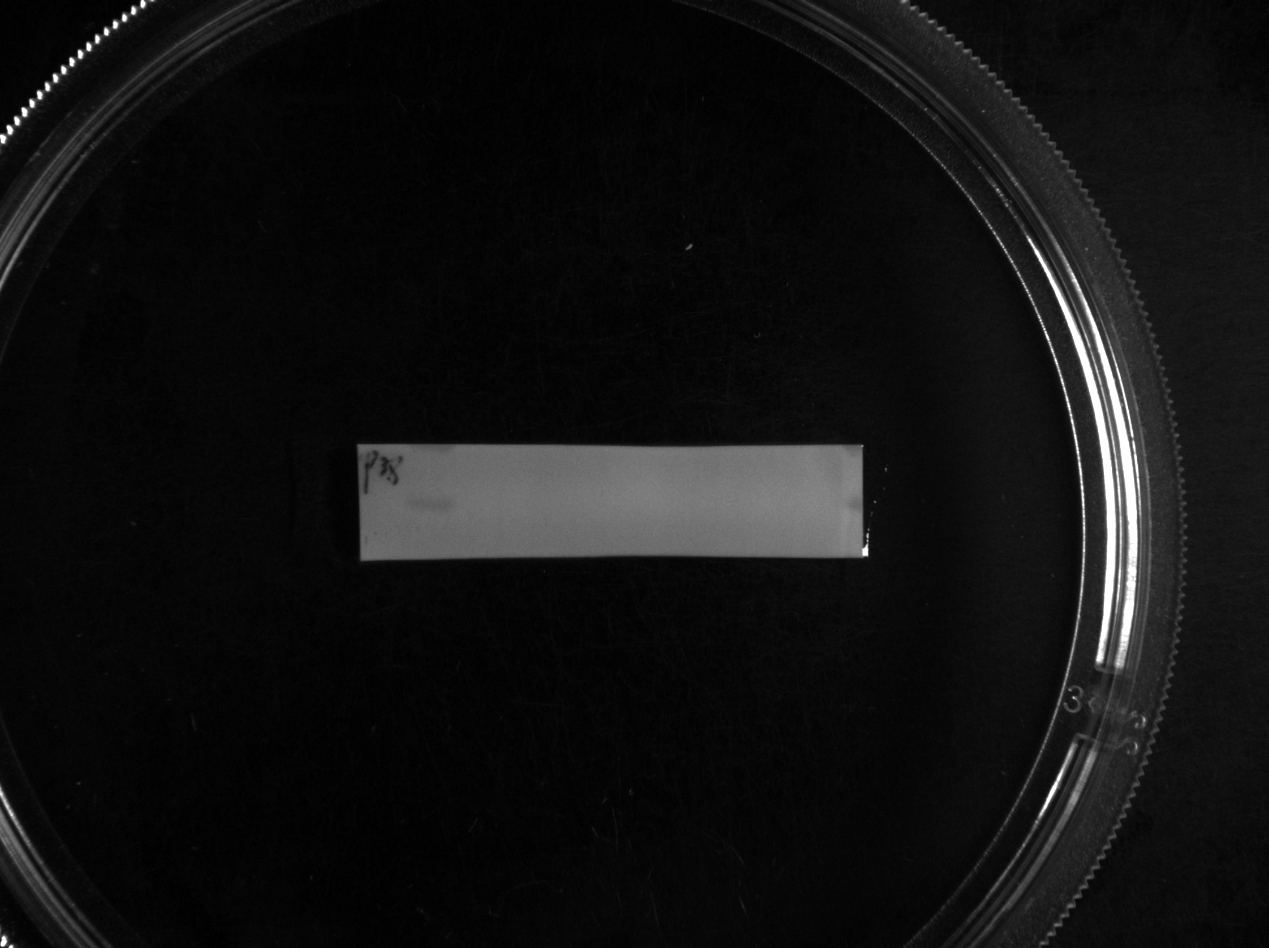


P38

(Colorimetric)


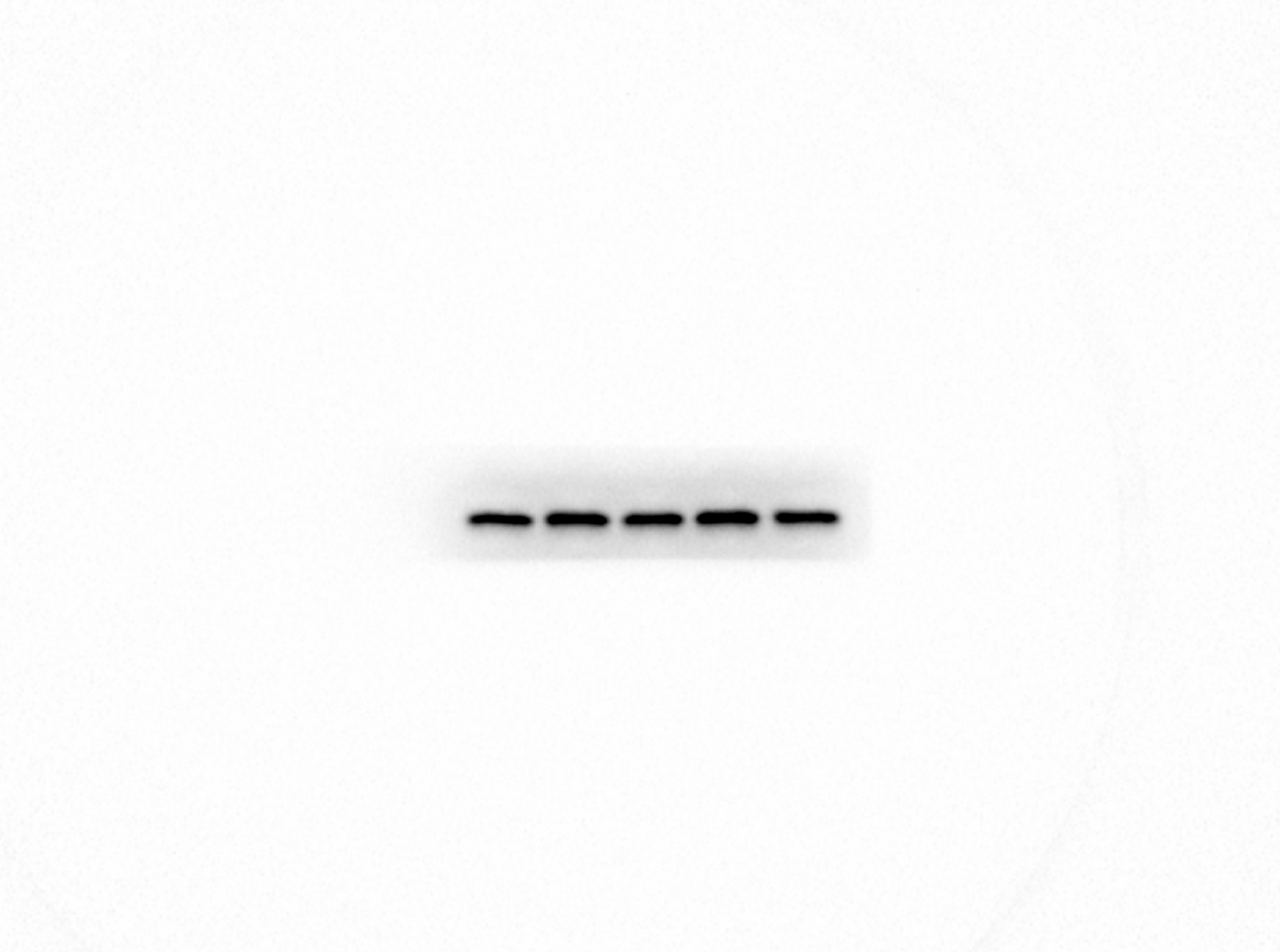


P38

(Chemi)


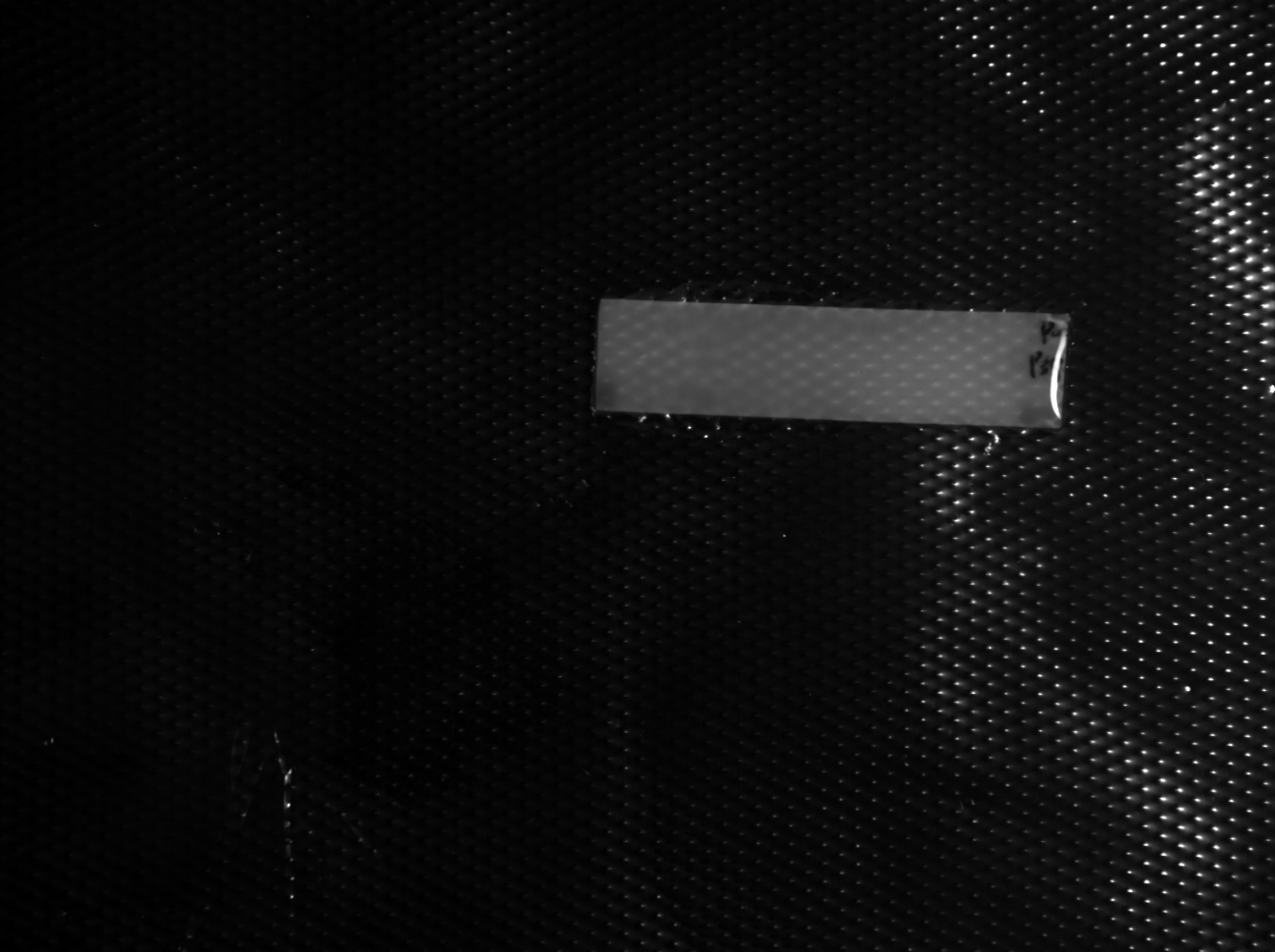


P-P38

(Colorimetric)


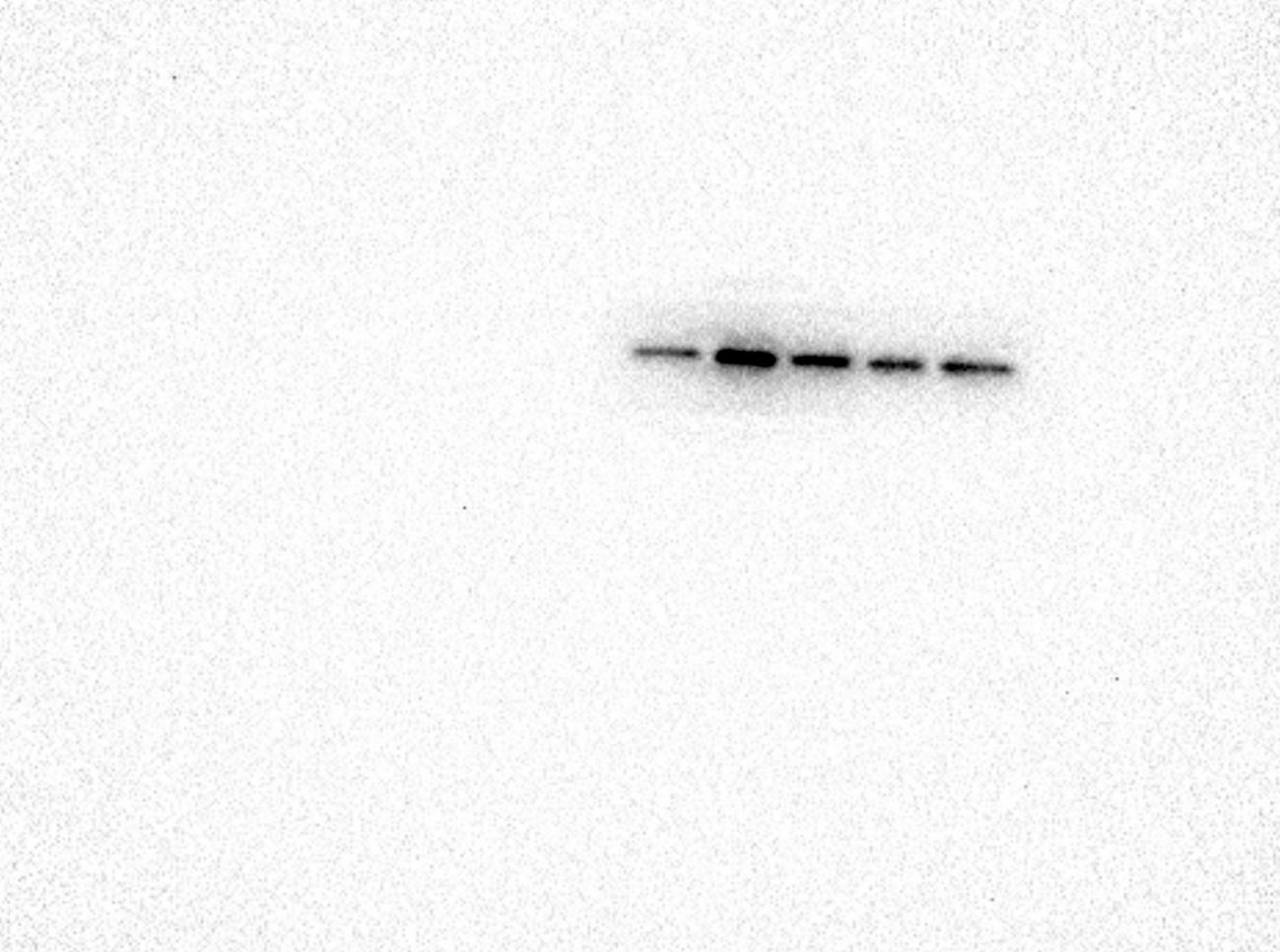


P-P38

(Chemi)

**Figure 6**


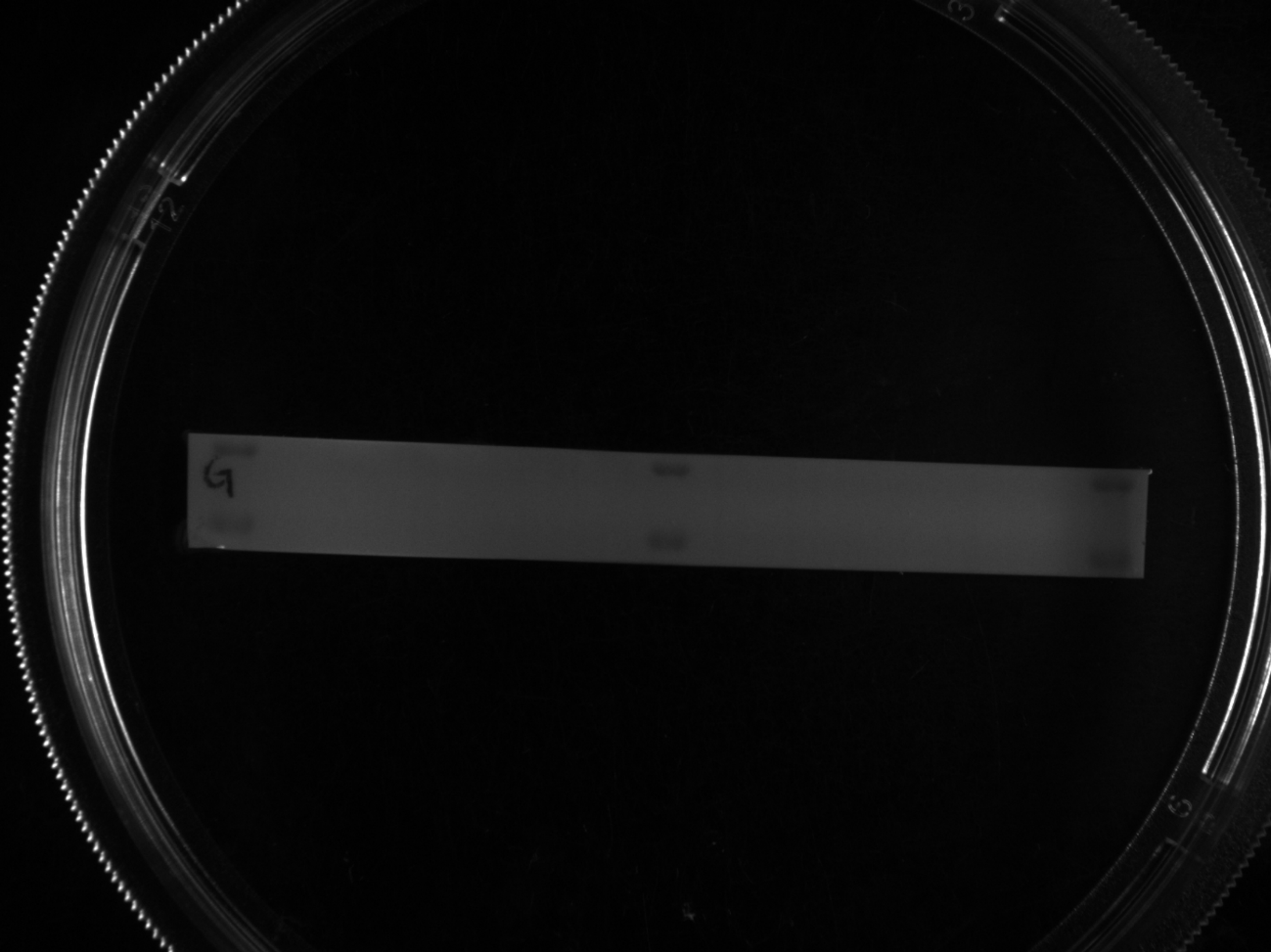


GAPDH-left

(Colorimetric)


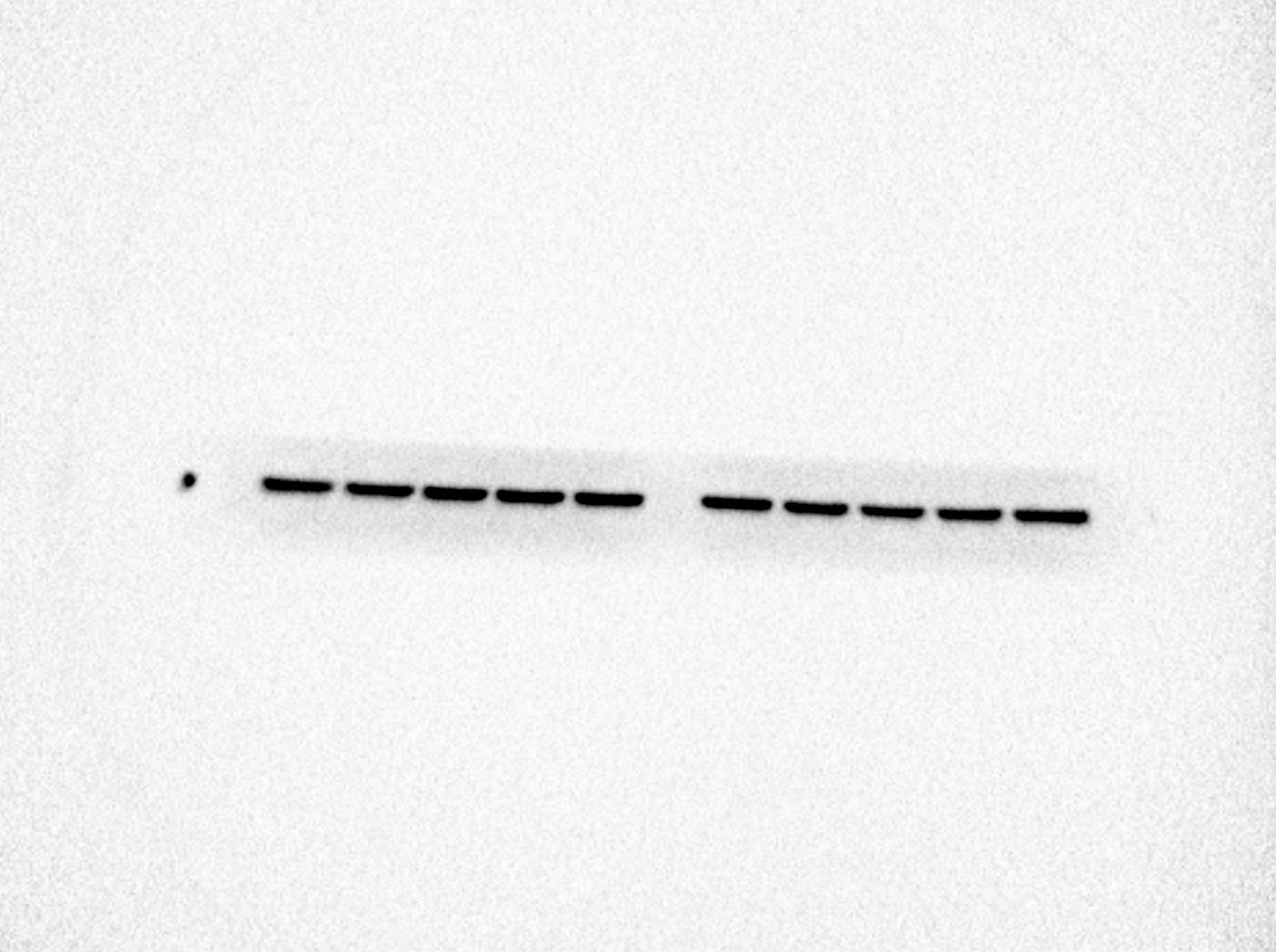


GAPDH-left

(Chemi)


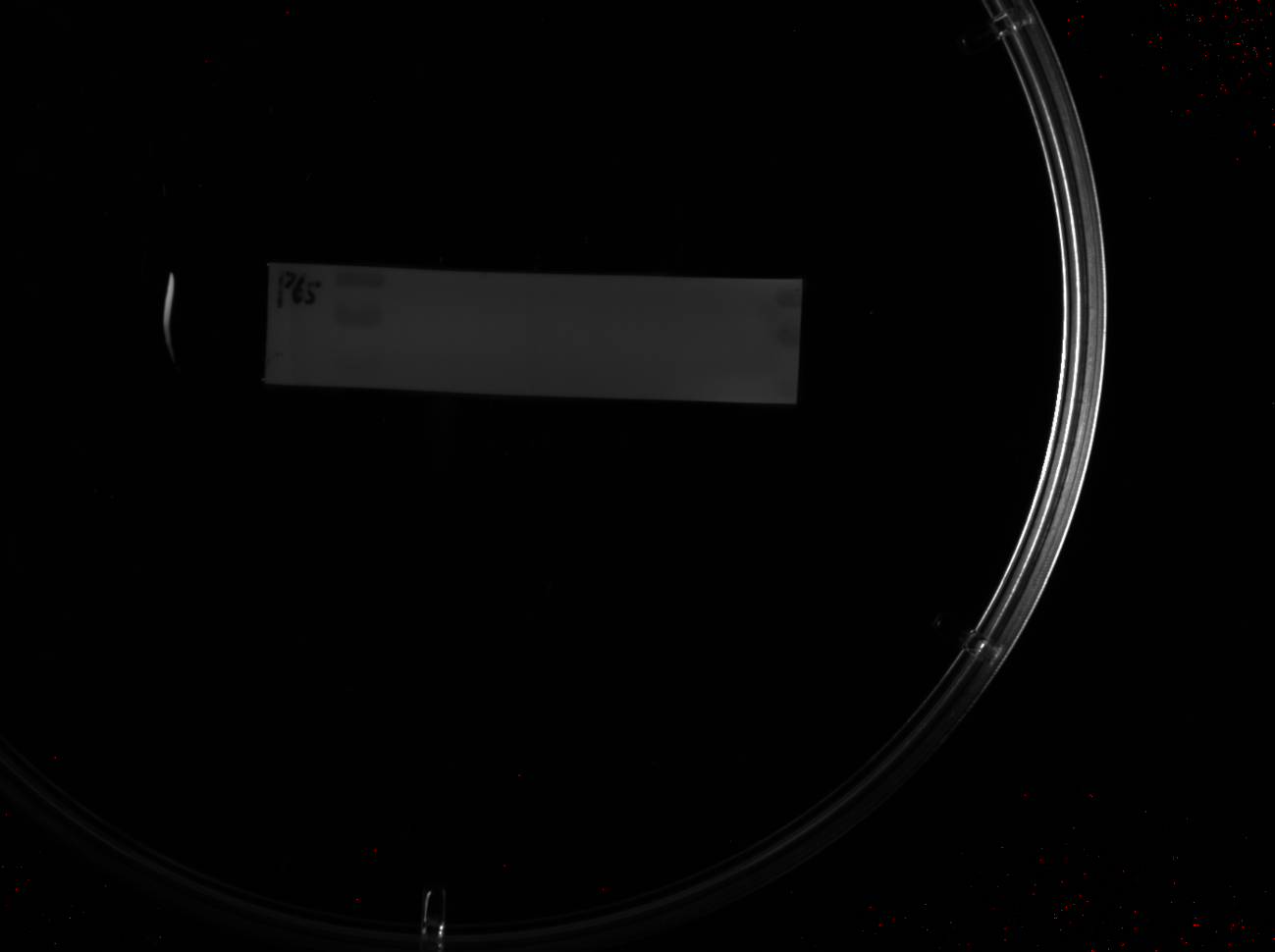


P65

(Colorimetric)


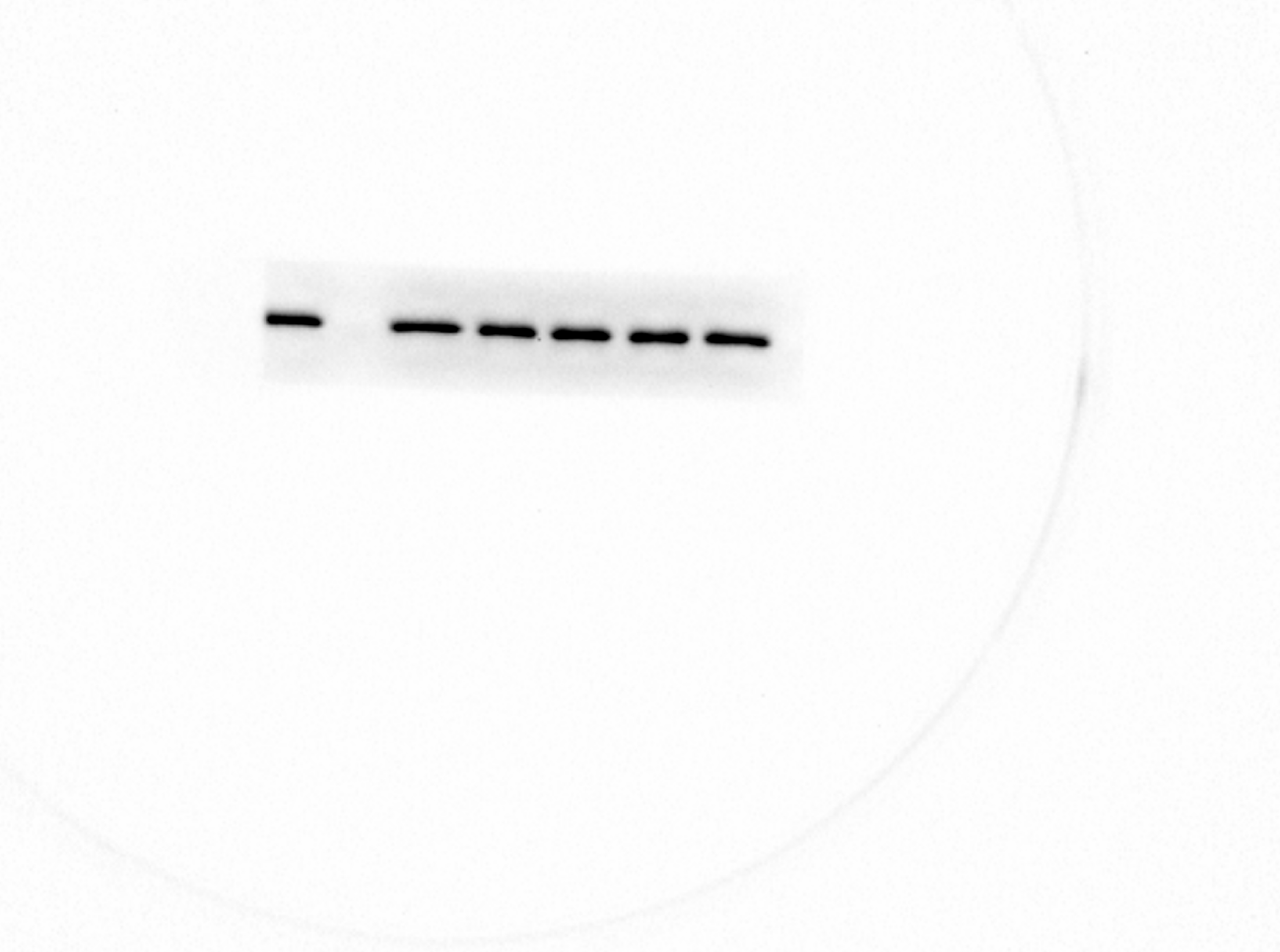


P65

(Chemi)


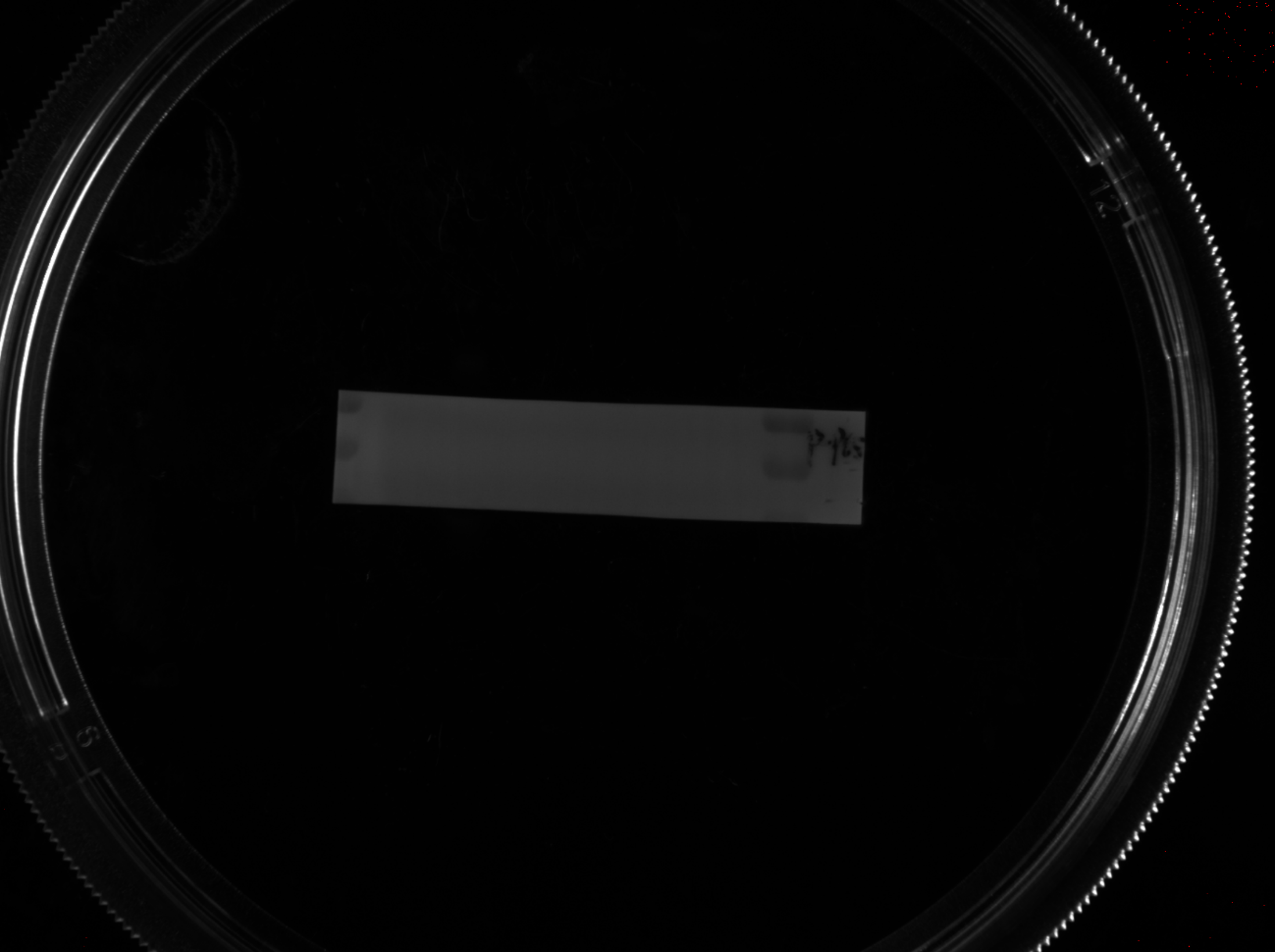


P-P65

(Colorimetric)


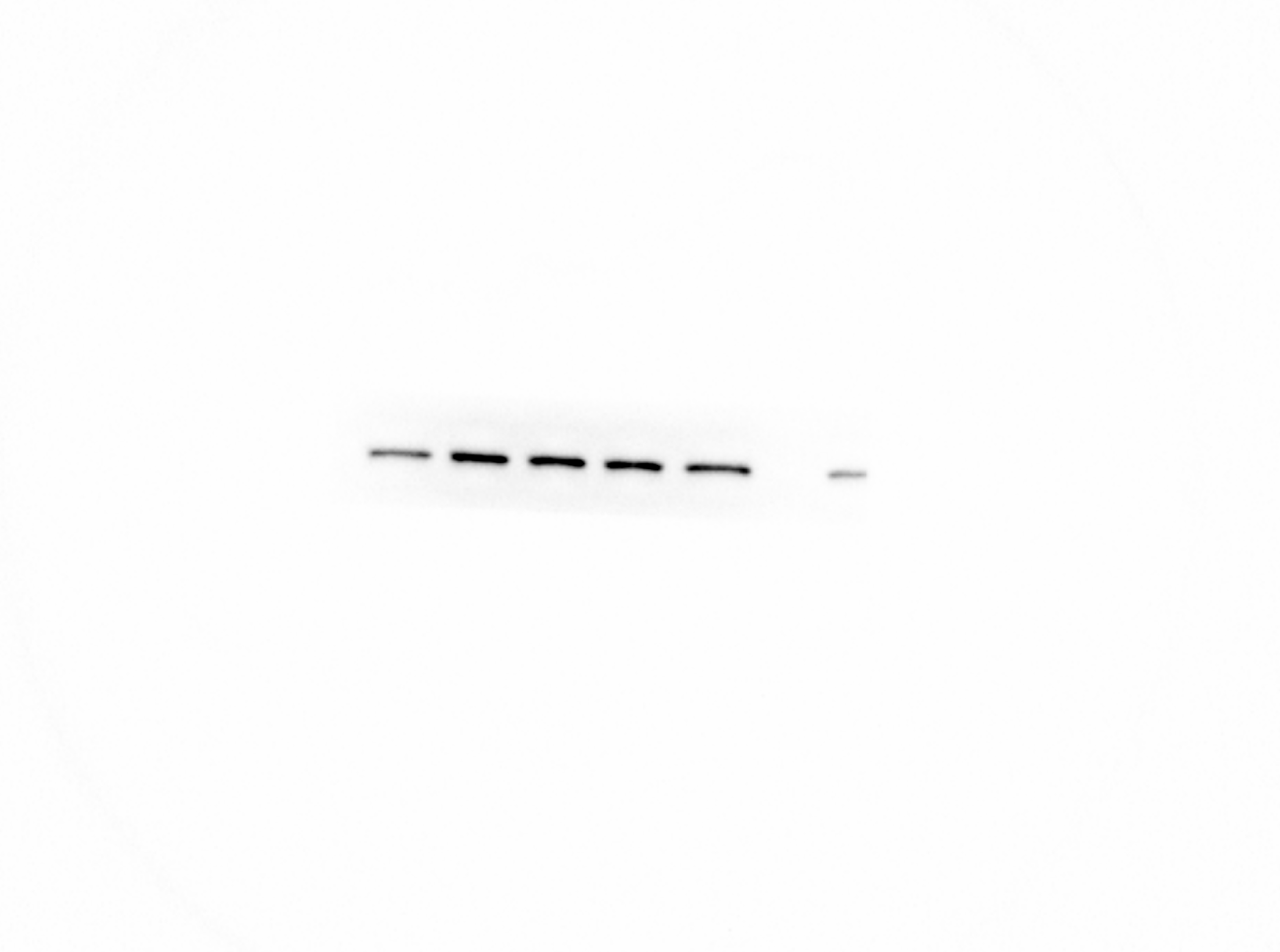


P-P65

(Chemi)


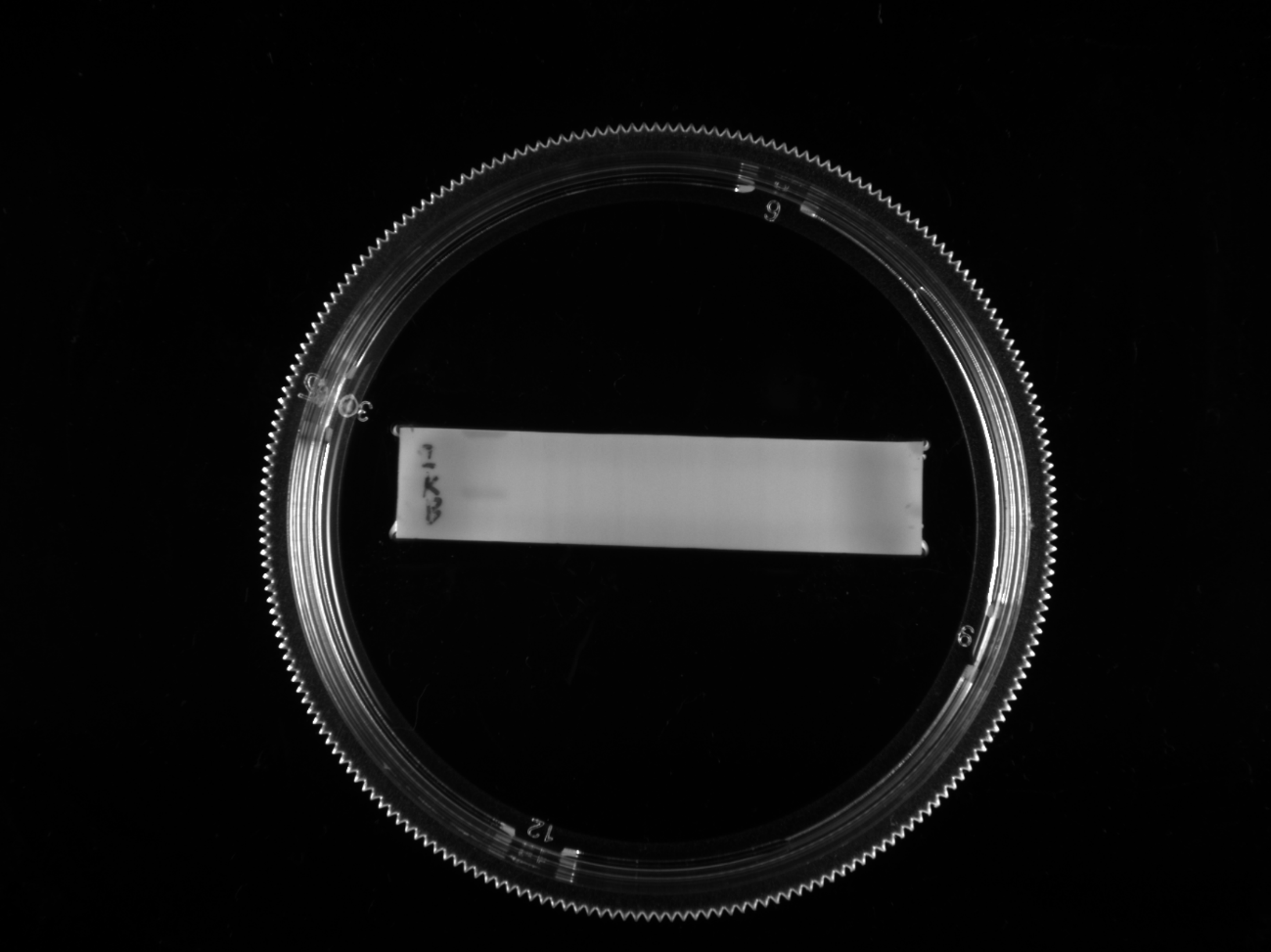


IKB

(Colorimetric)


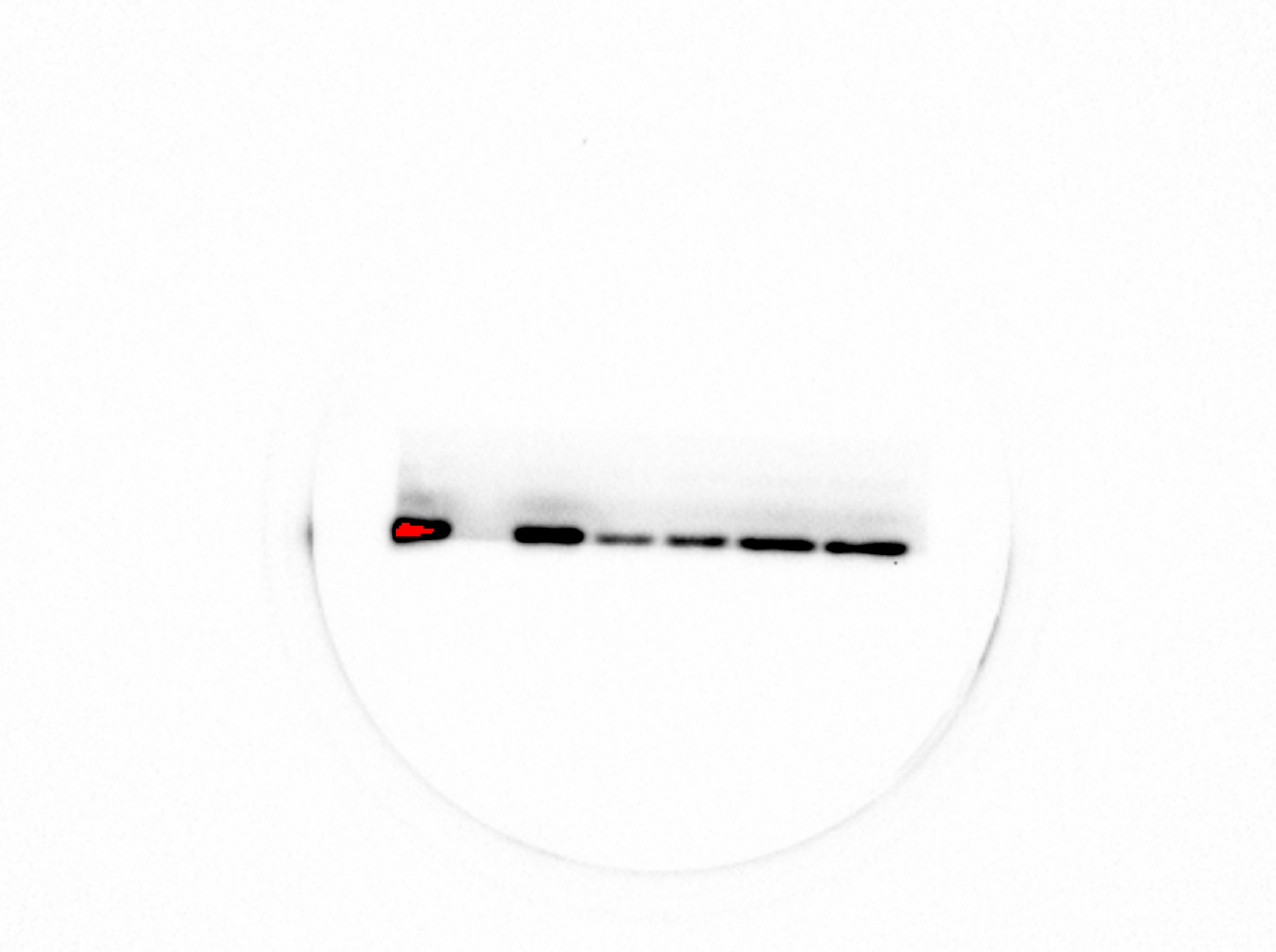


IKB

(Chemi)


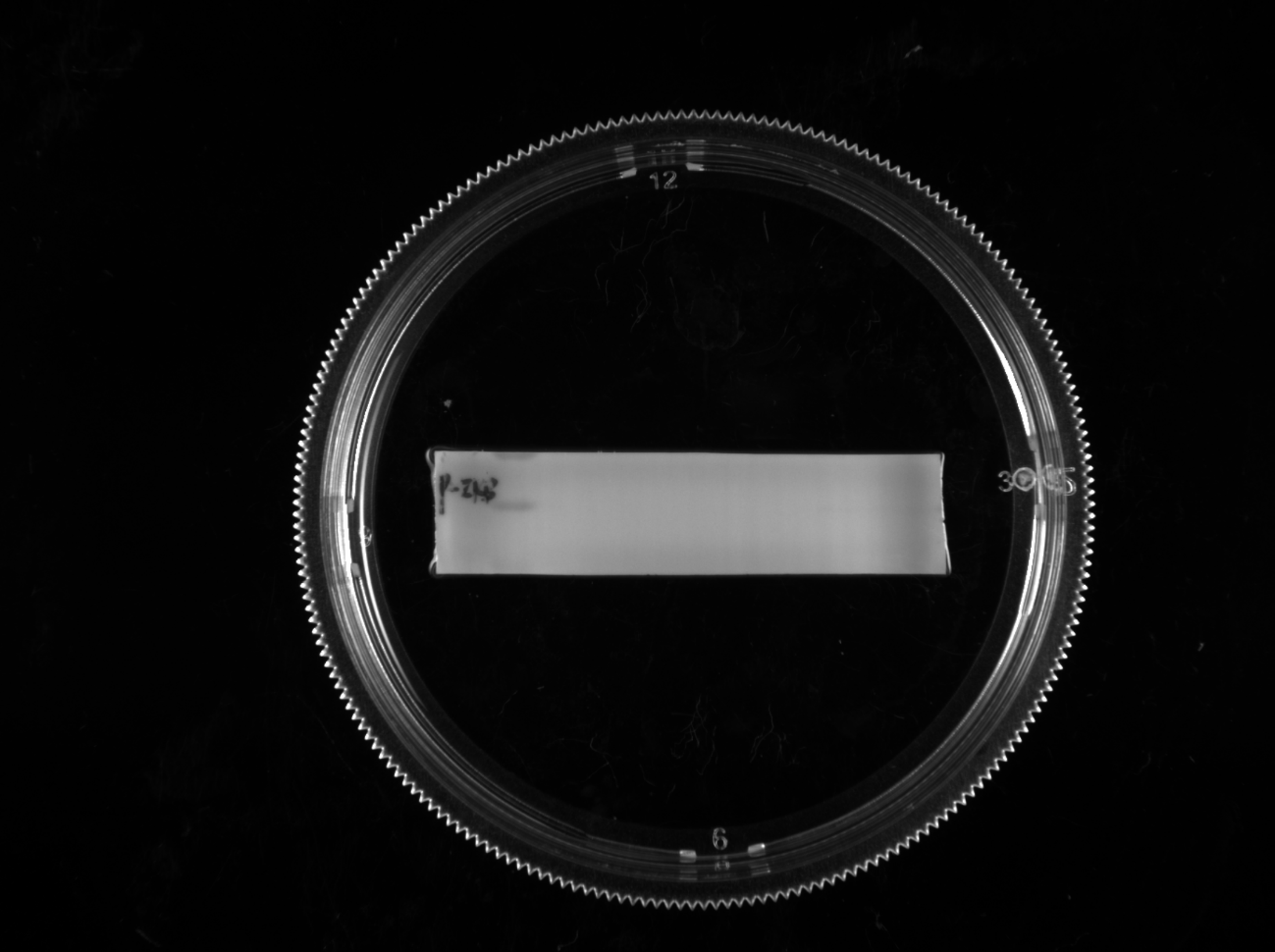


P-IKB

(Colorimetric)


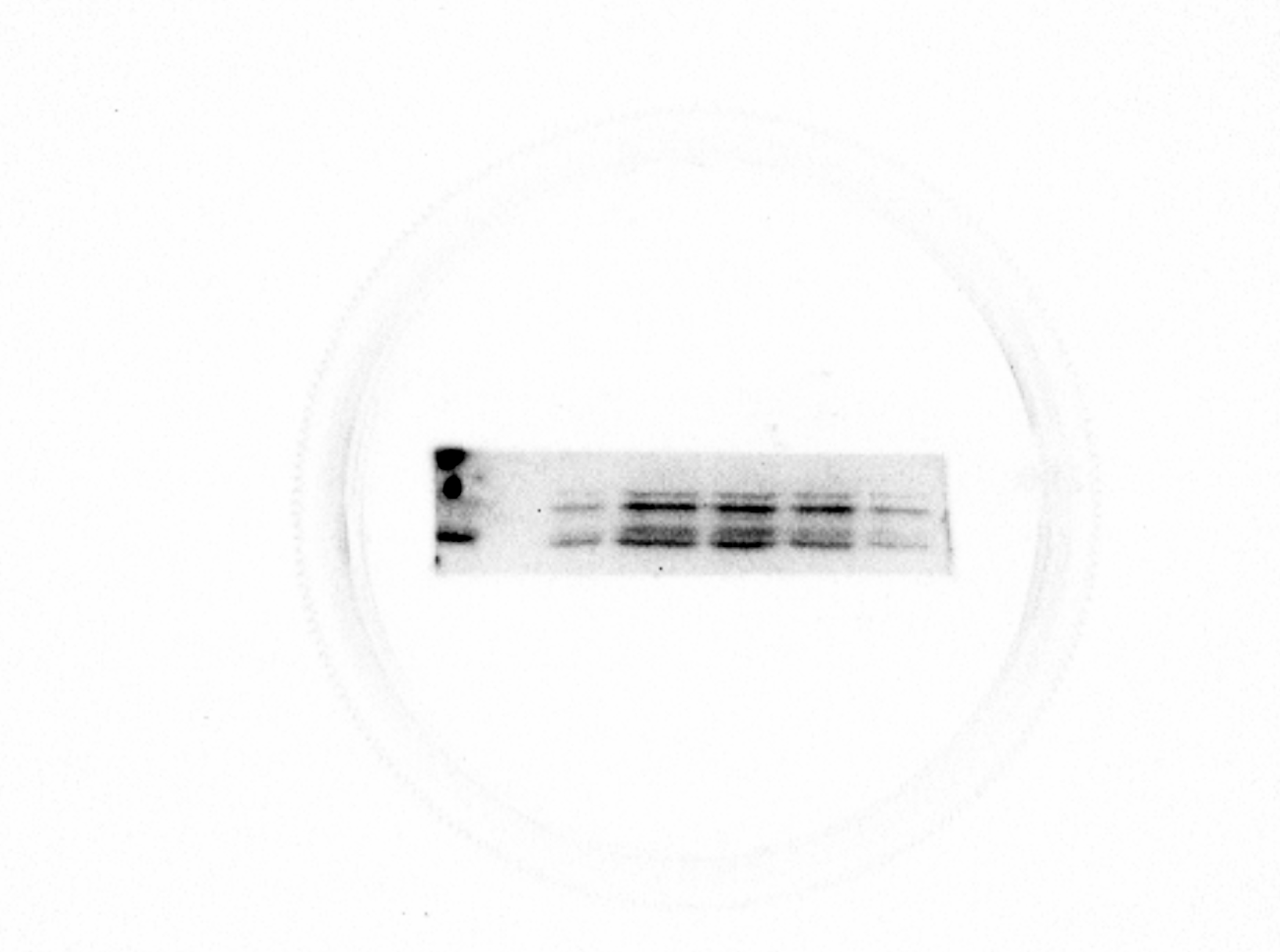


P-IKB

(Chemi)


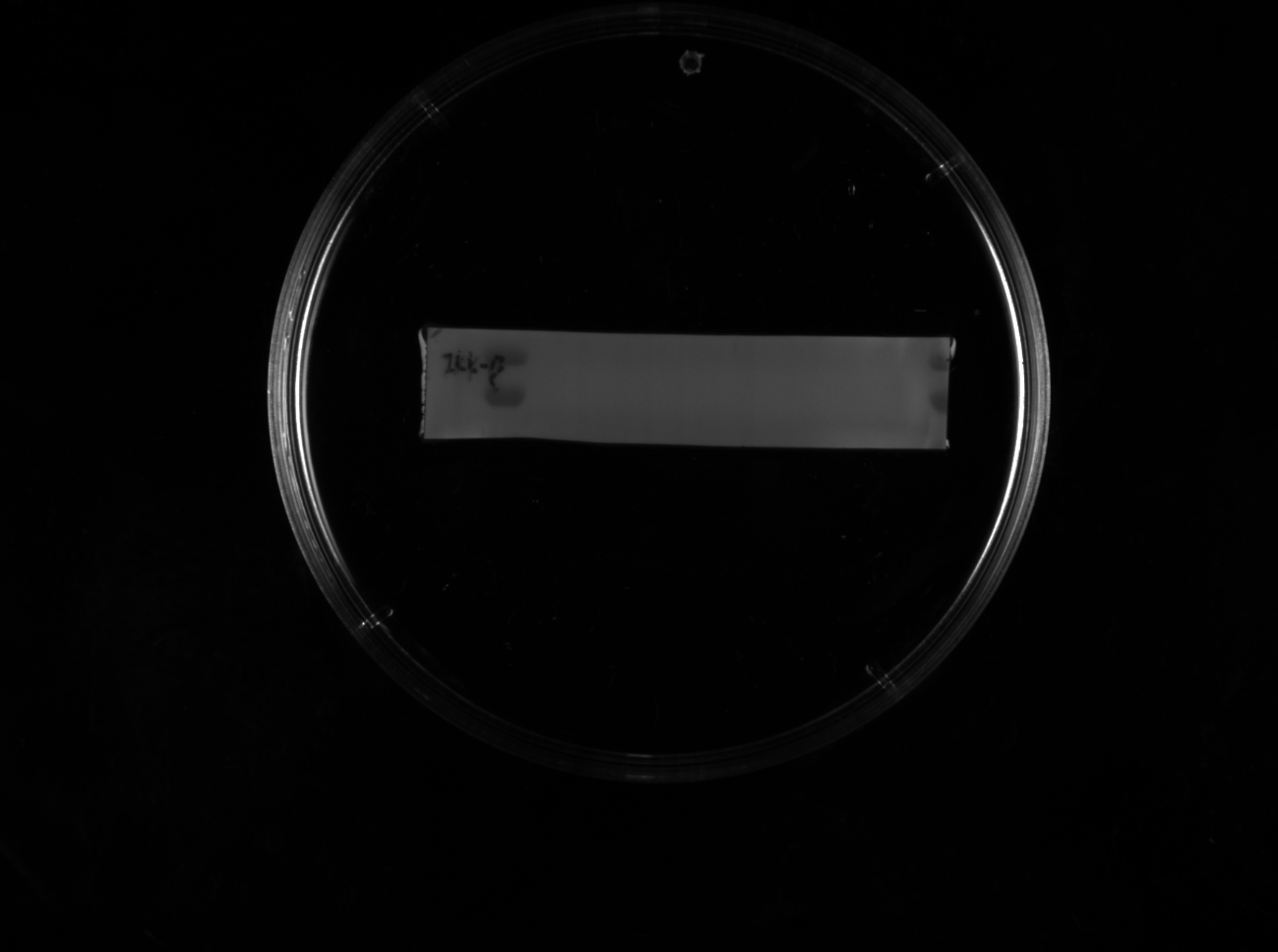


IKK-β

(Colorimetric)


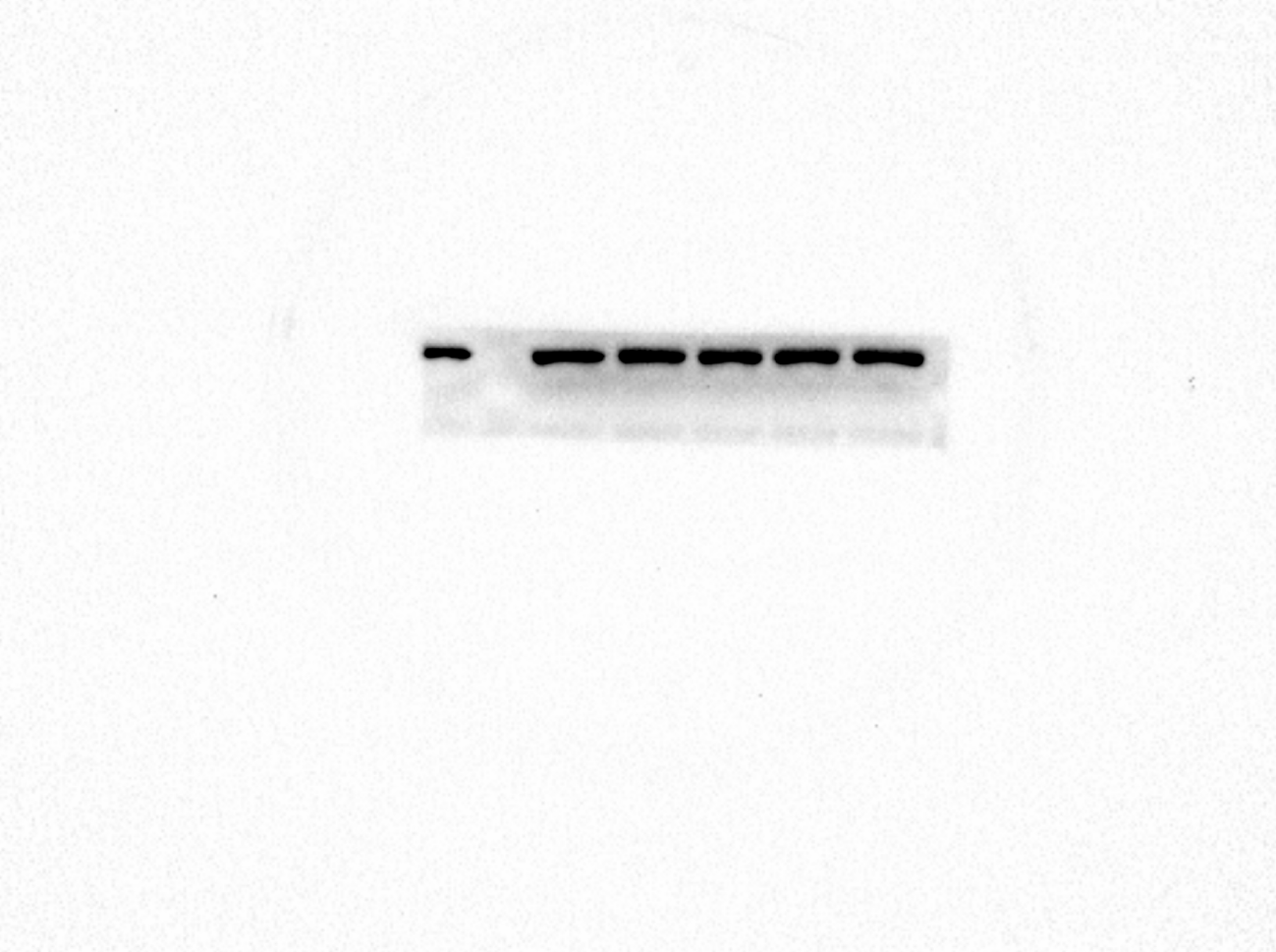


IKK-β

(Chemi)


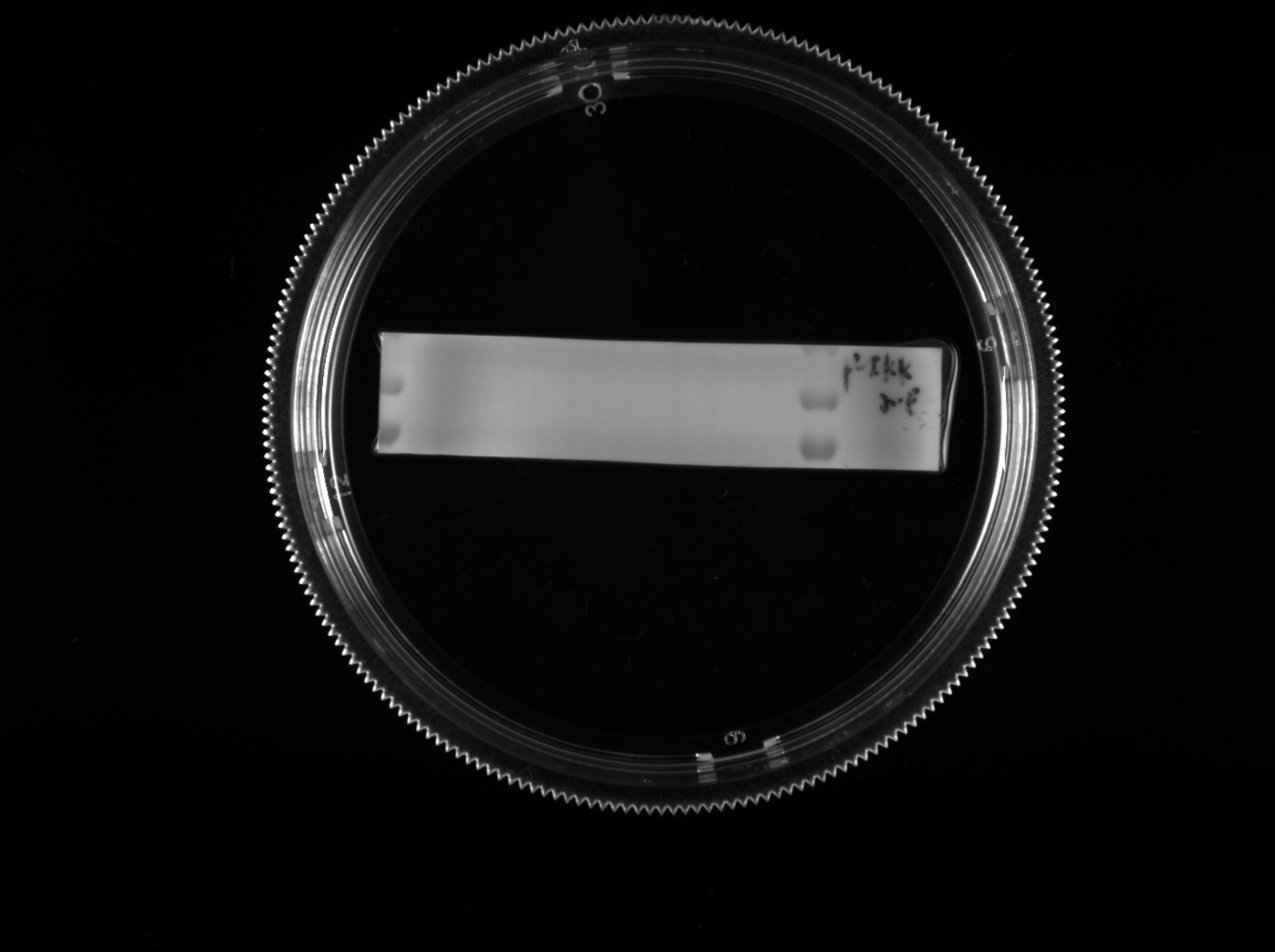


P-IKKαβ

(Colorimetric)


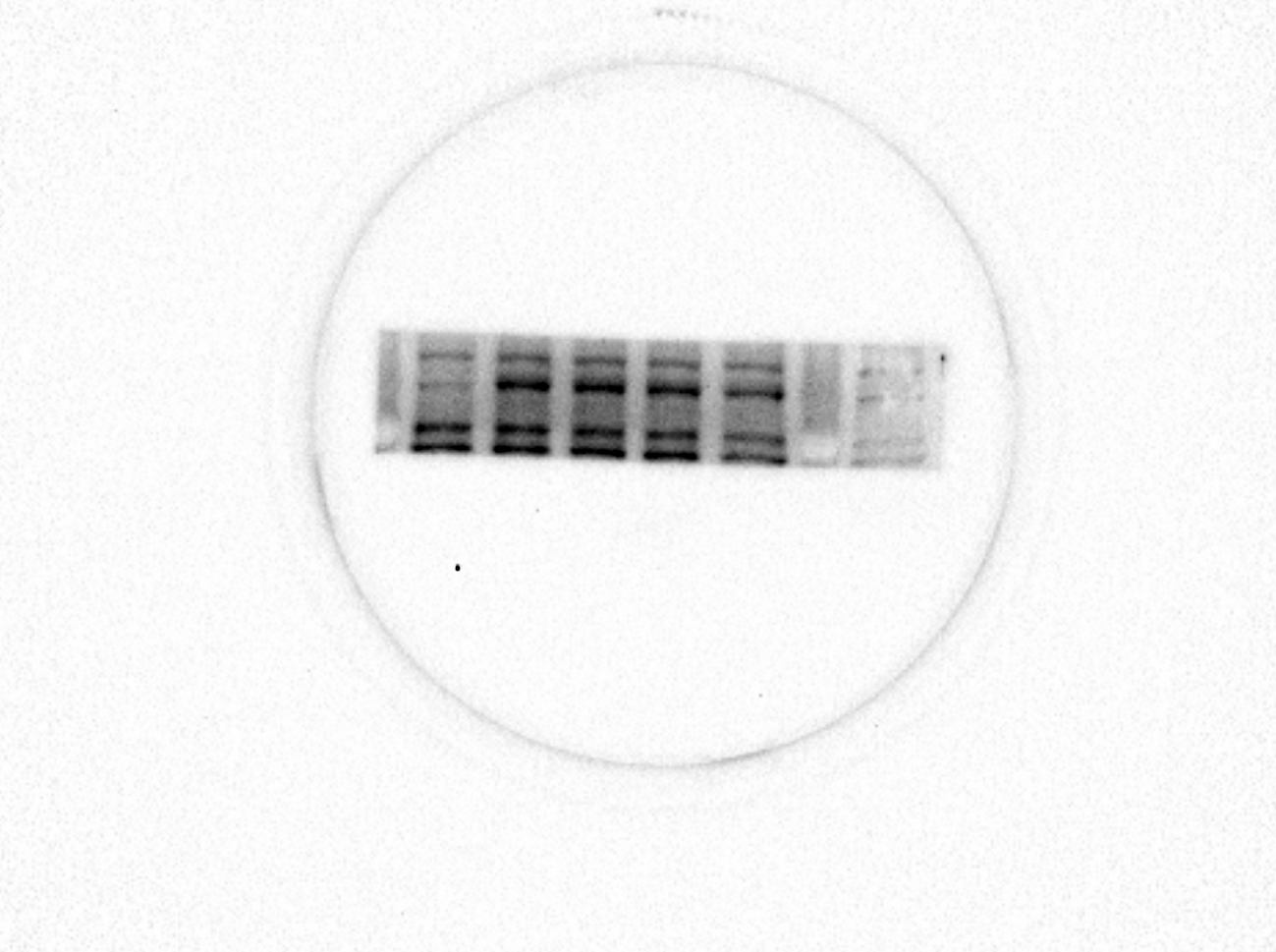


P-IKKαβ

(Chemi)
